# New Sesterterpenoids from *Salvia mirzayanii* Rech.f. and Esfand. Stereochemical Characterization by Computational Electronic Circular Dichroism

**DOI:** 10.3389/fchem.2021.783292

**Published:** 2022-01-20

**Authors:** Foroogh Mirzania, Mahdi Moridi Farimani, Yaghoub Sarrafi, Samad Nejad Ebrahimi, Jakob Troppmair, Marcel Kwiatkowski, Hermann Stuppner, Mostafa Alilou

**Affiliations:** ^1^ Department of Phytochemistry, Medicinal Plants and Drugs Research Institute, Shahid Beheshti University, Tehran, Iran; ^2^ Department of Organic Chemistry, Faculty of Chemistry, University of Mazandaran, Babolsar, Iran; ^3^ Daniel-Swarovski Research Laboratory, Department of Visceral, Transplant and Thoracic Surgery, Innsbruck Medical University, Innsbruck, Austria; ^4^ Functional Proteo-Metabolomics, Department of Biochemistry, University of Innsbruck, Innsbruck, Austria; ^5^ Department of Pharmacognosy, Institute of Pharmacy, Center for Molecular Biosciences (CMBI), University of Innsbruck, Innsbruck, Austria

**Keywords:** sesterterpenoid, *salvia mirzayanii*, absolute configuration, cytotoxic activity, diterpenoid

## Abstract

Phytochemical investigation on the acetone extract of *Salvia mirzayanii* Rech. f. and Esfand. aﬀorded seven new isoprenoids including six new sesterterpenoids salvimirzacolide A-F (**1–6**), and one new nor-diterpenoid (**7**). Their structures were established by comprehensive spectroscopic and spectrometric data analysis (1D and 2D NMR, HRMS) and DP4+ NMR chemical shift probability calculation technique. Moreover, the absolute conﬁguration of compounds was determined by using electronic circular dichroism spectroscopy. Evaluation of antiproliferative properties of compounds isolated against four human melanoma cancer cells displayed no cytotoxic activity at the concentration range used.

## 1 Introduction

Sesterterpenes are a small group of terpenoids, obtained from wide-spreading sources having been isolated from terrestrial origin, lichens, marine sponges, algae, higher plants, insects, and diverse marine organisms (Liu et al., 2007; Máximo and Lourenço, 2018). Compared with di- and triterpenoids, sesterterpenoids are scarce in nature. Sesterterpenes show many interesting pharmacological properties, including cytotoxicity, anti-microbial, anti-angiogenic activities, antibioﬁlm, and platelet aggregation inhibition (Rustaiyan et al., 2007; Cabanillas et al., 2018). The genus *Salvia* is one of the few genera in the Lamiaceae family that produces sesterterpenes (Qingwen et al., 2021). Many of these *Salvia* species are medicinal plants included in some pharmacopoeias, and are also used for alimentary and cosmetic purposes. Among *Salvia* species, these rare and attractive sesterterpens were mainly isolated and identiﬁed from Iranian *Salvia* species encouraging us to undertake a systematic phytochemical investigation of some of these Iranian species (Moghaddam et al., 2010; Farimani et al., 2016; Tabefam et al., 2018).

*Salvia mirzayanii* is one of the important species of the Hormozgan region that is used for diarrhea, stomachache, infectious and inﬂammatory diseases, headache, wounds, and high blood cholesterol, and have been used from ancient times by native peoples (Soltanipour, 2007). In a previous phytochemical study, the sesterterpene lactone *salvimirzacolide* and eupatorin were reported from the aerial parts of *Salvia mirzayanii* (Moghaddam et al., 1998). Moreover, our previous studies on the secondary metabolites of *Salvia mirzayanii* led to the isolation of five new manoyloxide sesterterpenoids (Ebrahimi et al., 2014).

**FIGURE 1:**
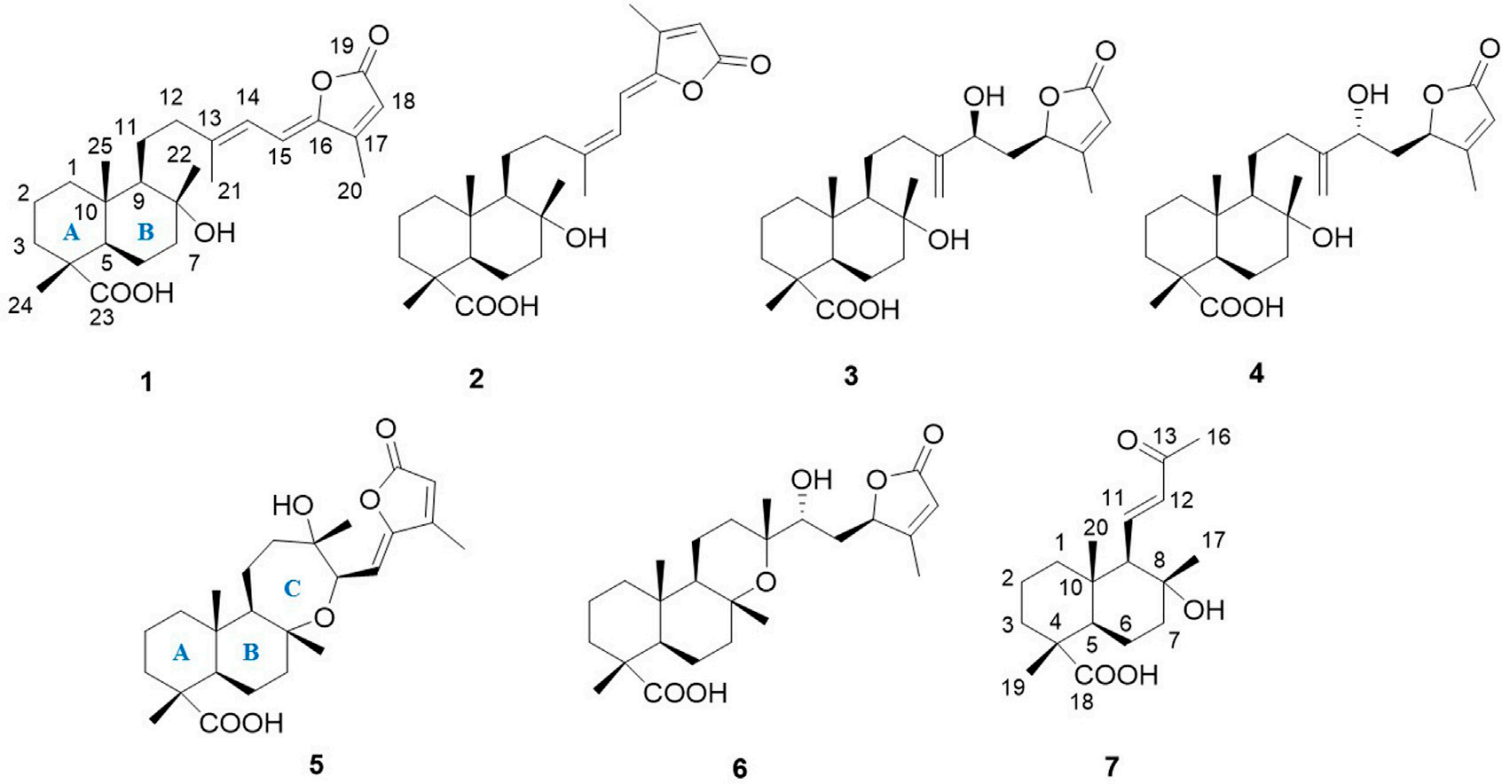
Chemical structures of compounds **1-7**.

In the course of this work, we have undertaken a further phytochemical investigation into the aerial parts of *S. mirzayanii* which resulted in the isolation and structural elucidation of six new sesterterpenes, salvimirzacolide A-F (**1–6**), along with one nor-diterpene (**7**) for the first time (Figure 1).

## 2 Results and Discussion

The structure elucidation was performed using extensive NMR spectroscopy and HRMS (Orbitrap), and the absolute configuration was established by comparison of calculated and experimental electronic circular dichroism (ECD) spectra.

**FIGURE 2:**
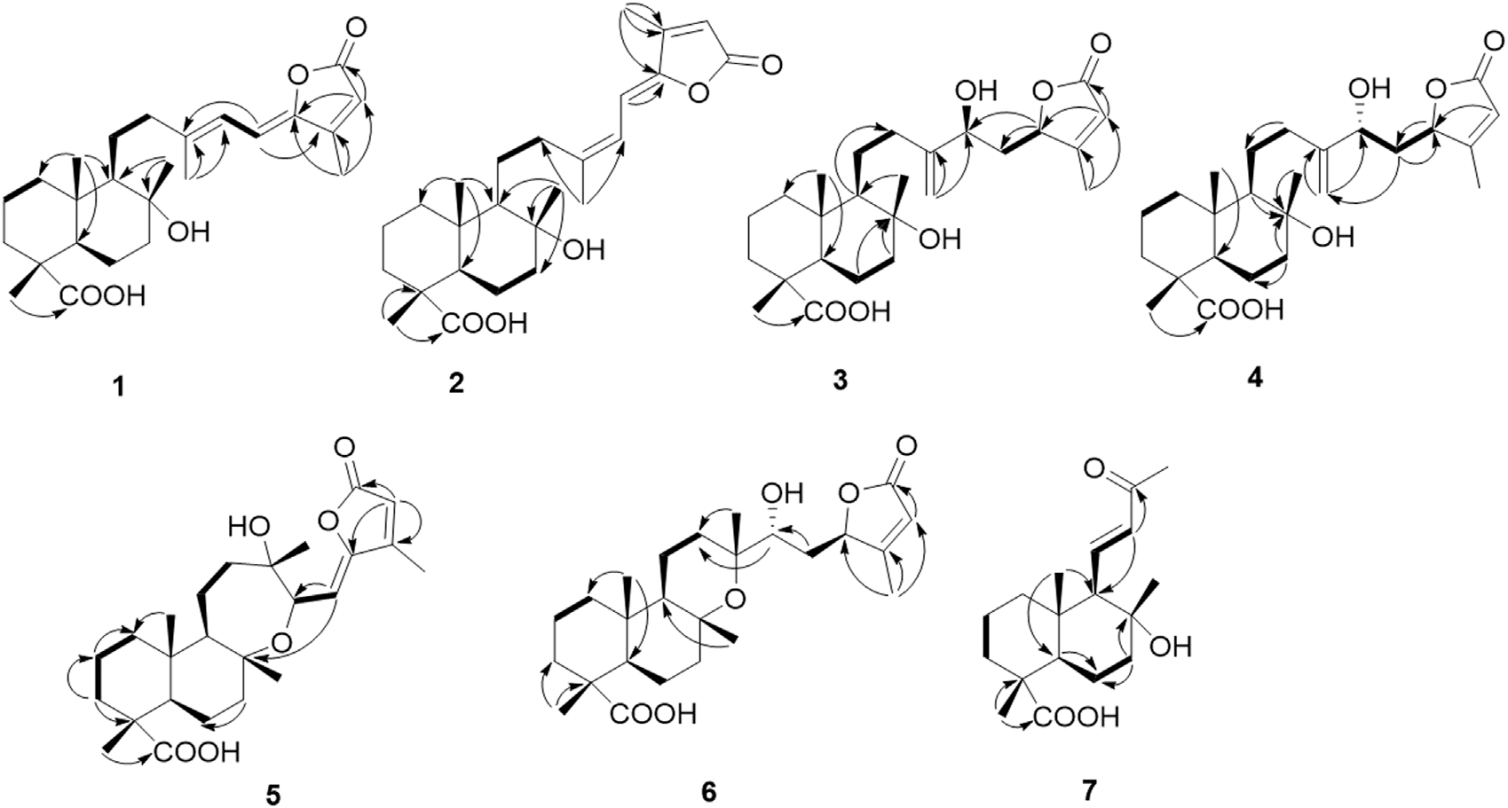
Key ^1^H–^1^H COSY (bold) and HMBC (H→C) correlations of **1-7**.

**FIGURE 3:**
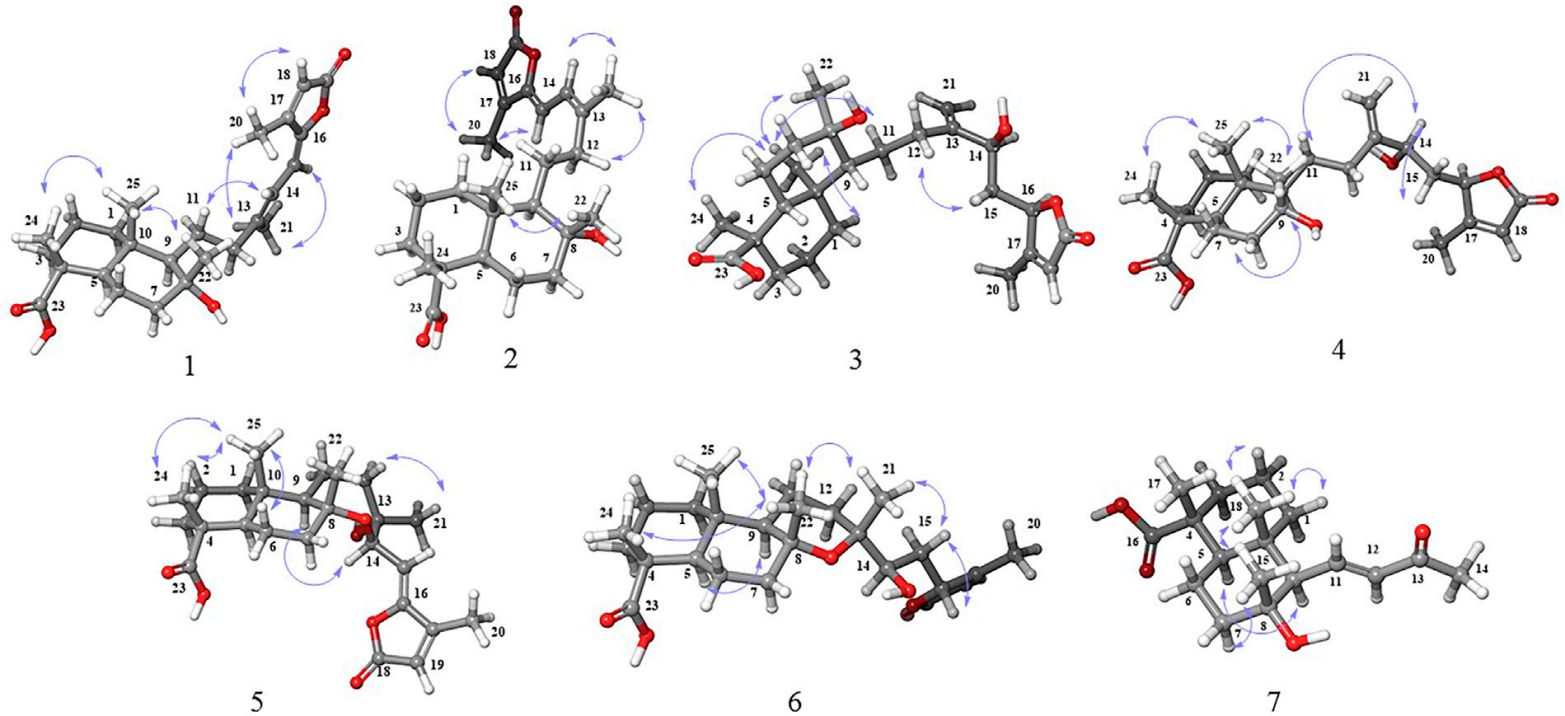
NOESY (H↔H) correlations of compounds **1-7**.

**FIGURE 4:**
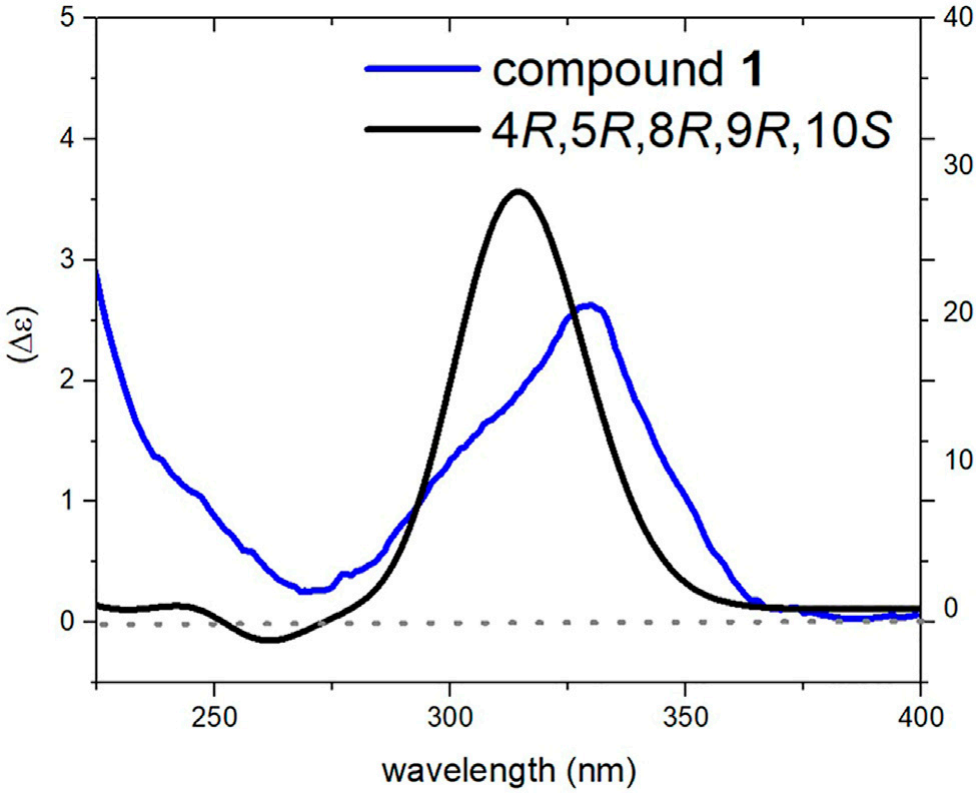
Comparison of experimental and calculated ECD spectra of compound **1** at TD-DFT/cam-B3LYP/6-31G(d,p) level of theory in MeOH.

Compound **1** was isolated as a colorless gum. The HRESIMS of **1** showed a molecular ion at *m/z* 831.5056 [2M–H]^−^ (calcd 831.5053), indicating a molecular formula of C_25_H_36_O_5_ and 8 indices of hydrogen deficiency. The DEPTq spectrum (Table 1) showed exactly 25 carbon resonances, which were assigned with the aid of HSQC spectra as 5 methyls, 7 methylenes, 5 methines, and 8 quaternary carbons. The DEPTq spectrum showed resonances for three double bonds (δ_C_ 149.2, 116.4), (δ_C_ 107.3, 148.0), and (*δ*_C_ 154.5, 114.8), two carbonyl groups (*δ*_C_ 169.8) and (*δ*_C_ 180.0), and one oxygenated carbon (*δ*_C_ 73.0). The ^1^H NMR spectrum (Table 1) showed resonances of five methyl singlets at *δ*_H_ 0.89 (H-25), *δ*_H_ 1.14 (H-24), *δ*_H_ 1.15 (H-22), *δ*_H_ 1.97 (H-21), and *δ*_H_ 2.24 (H-20). The structure of **1** was solved by the HMBC correlations initiated from the methyl groups. HMBC correlations were observed from H-24 (*δ*_H_ 1.14) to C-23 (*δ*_C_ 180.0), C-3 (*δ*_C_ 36.6), C-4 (*δ*_C_ 45.3), and C-5 (*δ*_C_ 50.1), from H_3_-25 (*δ*_H_ 0.89) to C-1 (*δ*_C_ 38.9), C-5 (*δ*_C_ 50.1), and C-9 (*δ*_C_ 61.3), from H_3_-22 (*δ*_H_ 1.15) to C-7 (*δ*_C_ 43.3), C-8 (*δ*_C_ 73.0), and C-9 (*δ*_C_ 61.3), from H_3_-21 (*δ*_H_ 1.97) to C-12 (*δ*_C_ 42.5), C-13 (*δ*_C_ 149.2), and C-14 (*δ*_C_ 116.4), and from H_3_-20 (*δ*_H_ 2.24) to C-16 (*δ*_C_ 148.0), C-17 (*δ*_C_ 154.5), and C-18 (*δ*_C_ 114.8) and confirmed the gross structure of **1** as a bicyclic sesterterpenoid (Figures 1, 2). The relative conﬁguration was derived from coupling constants (^3^ *J*_H–H_) and diagnostic NOESY correlations. NOESY cross peaks between H-24 (*δ*_H_ 1.14), H_3_-25 (*δ*_H_ 0.89), and H-22 (*δ*_H_ 1.15) confirmed their cofacial orientation (Figure 3). A coupling constant of 11.8 Hz was observed between H-14 and H-15 which is consistent with an anti-orientation between them. In addition, diagnostic NOESY correlations were observed from H-14 to H-12*α* and H-12*β* suggesting the *cis* relationship of H-14 with C-12. Thus, C-15 carbon should be in the *trans* position relative to C-12. NOESY correlation between H-15 and H-21 confirmed this geometry. Finally, the Z-geometry of the C-15-C-16 double bond was elucidated via the NOESY cross-peak between H-15 and H_3_-20. The ECD spectrum of **1** showed a positive Cotton eﬀect (CE) at 312 nm. The calculated ECD spectrum for the 4*R*, 5*R*, 8*R*, 9*R*, 10*S* stereoisomer showed excellent ﬁt with the experimental data, with a positive CE around 330 nm (π→ π* transition) of the α, *β*-unsaturated moiety (Figure 4). Therefore, the structure of compound **1** was elucidated to be (4*R*, 5*R*, 8*R*, 9*R*, 10*S*)-8α-hydroxylabd-13,15,17-trien-19,16; 23-diolide and named as salvimirzacolide A.

**TABLE 1:** ^1^H and ^13^C NMR spectroscopic data of compounds **1**-**4** (500 MHz for **1** and **2** and 600 MHz for **3** and **4**, *δ* in ppm, *J* in Hz)

Position	1		2		3		4	
	δ_H_	δ_C_	δ_H_	δ_C_	δ_H_	δ_C_	δ_H_	δ_C_
1α	1.16, m	38.8	1.18, m	38.8	1.10, m	38.8	1.07, m	38.9
1β	1.75, m	—	1.78, m	—	1.70, m	—	1.68, m	—
2α	1.69, m	16.9	1.67, m	17.2	1.60, m	17.3	1.59, m	17.5
2β	1.58, m	—	1.56, m	—	1.57, m	—	1.55, m	—
3α	1.56, m	36.2	1.57, m	36.6	1.60, m	36.8	1.59, m	37.0
3β	1.82, m	—	1.80, m	—	1.76, m	—	1.75, m	—
4	—	45.8	—	48.1	—	47.1	—	47.3
5	1.81, m	50.1	1.82, m	50.5	1.79, m	50.1	1.77, m	50.4
6α	1.09, m	22.3	1.28, m	22.5	1.28, m	23.8	1.45, m	24.1
6β	1.28, m	—	1.47, m	—	1.40, m	—	1.67, m	—
7α	1.81, m	42.7	1.81, m	43.3	1.81, m	43.6	1.80, m	44.1
7β	1.55, m	—	1.55, m	—	1.49, m	—	1.49, m	—
8	—	73.8	—	73.0	—	74.5	—	75.0
9	1.23, m	61.3	1.25, m	61.2	1.25, m	60.6	1.25, m	60.3
10	—	36.5	—	36.9	—	40.7	—	38.5
11a	1.69, m	23.1	1.59, m	24.5	1.60, m	23.1	1.38, m	23.4
11b	1.45, m		1.41, m		1.50, m		1.28, m	—
12a	2.26, m	43.3	2.28, m	36.2	2.16, m	35.3	2.09, m	34.7
12b	2.42, m	—	2.57, m	—	2.19, m	—	2.27, m	—
13	—	149.2	—	149.5	—	151.5	—	151.7
14	6.37, d (11.7)	117.3	6.29, d (11.9)	117.2	4.33, t (6.18)	71.1	4.46, m	72.0
15a	6.27, d (11.7)	107.9	6.35, d (11.8)	107.5	1.80, m	38.3	1.47, m	39.5
15b	—	—	—	—	2.12, m	—	2.04, m	—
16	—	148.0	—	148.0	4.83, m	82.5	5.12, m	82.2
17	—	154.6	—	157.4	—	169.3	—	169.7
18	5.94, s	114.3	5.94, s	113.9	5.79, s	116.5	5.78, s	116.7
19	—	169.8	—	169.7	—	173.1	—	173.5
20	2.23, s	10.0	2.21, s	10.3	2.08, s	14.1	2.05, s	14.1
21	1.95, s	15.5	1.98, s	23.6	4.98, s-5.15, s	111.9	4.89, s-5.08, s	111.6
22	1.16, s	22.4	1.15, s	22.6	1.15, s	24.1	1.13, s	24.1
23	—	181.5	—	181.9	—	183.1	—	183.7
24	1.14, s	15.5	1.14, s	15.5	1.14, s	16.1	1.11, s	16.4
25	0.89, s	14.7	0.88, s	14.4	0.84, s	15.8	0.81, s	16.0

**FIGURE 5:**
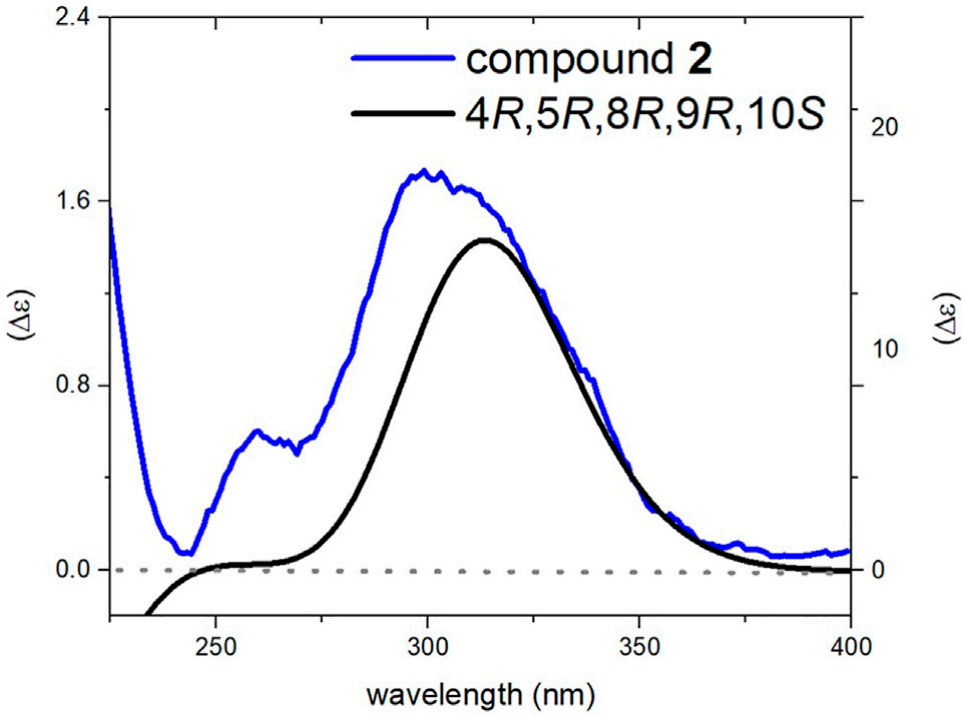
Comparison of experimental and calculated ECD spectra of compound **2** at TD-DFT/cam-B3LYP/6-31G(d,p) theory in MeOH.

Compound **2** had a molecular formula of C_25_H_36_O_5_, as deduced from the HRESIMS *m/z* 831.5054 [2M–H]^−^ (calcd 831.5053), corresponding to 8 indices of hydrogen deficiency. Its NMR features (Table 1) were closely related to those of **1**. The marked differences in the ^13^C NMR data of **2** in comparison to those of **1** were consistent with the diamagnetically shifted signal of C-12 (*δ*_C_ 36.0) with Δ*δ* = −6.5 ppm, and paramagnetically shifted signal of C-21 (*δ*_C_ 23.4) with Δ*δ* = +8.1 ppm (Table 1). Careful inspection of the HMBC and ^1^H–^1^H COSY correlations revealed that compound **2** possessed the same flat structure of **1**. According to the changes mentioned above in the chemical shifts, it was assumed that **2** should be the stereoisomer of **1** in C-14, something that the NOESY spectrum confirmed (Figure 3). As compound **1**, the relative conﬁguration of the rigid rings was solved by observing diagnostic cross-peaks between H_3_-24 (*δ*_H_ 1.14), H_3_-25 (*δ*_H_ 0.88), and H_3_-22 (*δ*_H_ 1.15). In the side chain a coupling constant of 11.8 Hz was observed between H-14 and H-15 similar to that of **1**, and a Z-geometry was also distinguished for the C-15-C-16 double bond based on the NOESY cross-peak between H-15 and H_3_-20. But in contrast to **1**, strong NOESY correlations were observed between H-14 and H_3_-21 as well as between H-15 and H-12α, and H-12β suggesting the *trans* relationship between H-14 and C-12. The above-mentioned up-field shift of C-12 also corresponds to the fact that C-12 and C-15 should be on the same side of the C-13-C-14 double bond, effectively in a *syn* geometry (Hammann et al., 1991; Suzuki et al., 2009; Jacobsen, 2017). The experimental ECD spectrum of **2** was similar to that of **1**, and the configuration of stereogenic centers was thus established as 4*R*, 5*R*, 8*R*, 9*R*, 10*S*. The simulated ECD spectrum for proposed stereochemistry showed good fit with experimental data (Figure 5). So, the structure of compound **2** was elucidated to be (4*R*, 5*R*, 8*R*, 9*R*, 10*S*)-8α-Hydroxylabd-13,15,17-trien-19,16; 23-diolide and named as salvimirzacolide B.

**FIGURE 6:**
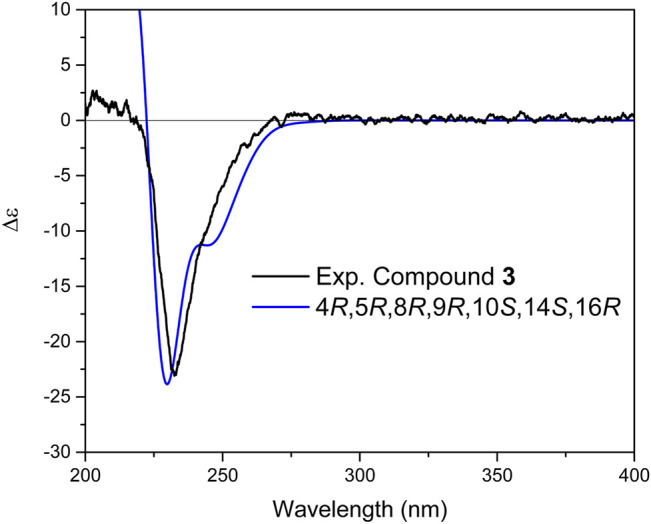
Comparison of experimental and calculated ECD spectra of compound **3** at TD-DFT/B3LYP/6-31+G(d,p) basis set and level of theory in acetonitrile.

Compound **3** was isolated as a colorless gum. A molecular formula of C_25_H_38_O_6_ was deduced from HRESIMS data [*m/z* 457.2557 [M+H]^+^; calcd. 457.2560] and using NMR data, accounting for seven indices of hydrogen deficiency. The ^13^C NMR spectrum (Table 1) showed 25 carbon resonances, which were identified by means of HSQC data as four methyl, nine methylenes, five methines, and seven quaternary carbons. The ^13^C NMR data showed signals for a di-substituted double bond (δ_C_ 151.5, 111.9), a trisubstituted double bond (δ_C_ 169.3, 116.5), and two carbonyl carbons (δ_C_ 173.1, 183.1). Three carbon signals at δ_C_ 71.1, 74.5, and 82.5 indicated the presence of oxygen-bearing sp^3^ carbons. According to the degree of unsaturation, the structure of **3** should be tricyclic due to the absence of any other sp or sp^2^ carbon signals. The ^1^H NMR spectrum (Table 1) of **3** showed the presence of four methyl singlets at δ_H_ 0.84, 1.14, 1.15, and 2.08, as well as three vinyl protons at δ_H_ 5.79, 4.98, and 5.15. The NMR data of **3** (Table 1) suggested that its structure resembled that of lachnocalyxolide B, a sesterterpenoid previously isolated from *Salvia lachnocalyx* Hedge by Farimani and Mazarei (2014). Inspection of the NMR data revealed lack of the C-6 oxygenated methine group in compound **3**, and rather the presence of an additional methylene group (*δ*_H-6_ 1.28, 1.40; *δ*_C-6_ 23.8). This suggested the replacement of the lactone moiety by a hydroxy acid in **3**. The relative configuration of the decalin moiety was assigned to be 4*R* ^*^, 5*R* ^*^, 8*R* ^*^, 9*R* ^*^, 10*S* ^*^, based on NOESY correlations between H-25, H-24, and H-22, as well as NOE between H-9 and H-5. To determine the relative configuration of centers C-14 and C-16, DP4+ NMR chemical shift probability calculation was implemented (Grimblat et al., 2015). Generated conformers for two stereoisomers, 14*S*, 16*R* and 14*R*, 16*R*, were subjected to geometrical optimization followed by NMR chemical shift calculation at CPCM/mPW1PW91/6-31+G(d,p)//B3LYP/6-31G(d) basis sets and level of theories. Calculation of DP4+ probability value indicated 14*S*, 16*R* (isomer 2) with probability of 100% to be the correct stereoisomer (Supplementary Figure S62). The results further supported by comparison of calculated total correlation coefficients for both isomers (0.99041 for isomer 1 and 0.99430 for isomer 2; Supplementary Figure S63). Additionally, calculation of ECD spectra for the aforementioned stereoisomers could further confirm both relative and absolute configuration of compound **3** (Figure 6).

It is worth mentioning that due to the flexibility of molecule and lack of NOE between ring A and B with the chiral centers in the side chain, the absolute stereochemistry of a 10-membered ring (Figure 1) in both isomers was assumed as 4*R*, 5*R*, 8*R*, 9*R*, 10*S*, by taking into account the biosynthetic pathway of these compounds, our previous reports, as well as data obtained in the current study for similar molecules (compounds **5** and **6**). Ultimately, the structure of **3** was elucidated as (4*R*, 5*R*, 8*R*, 9*R*, 10*S*, 14*S*, 16*R*)-8,14-Dihydroxylabd-13 (21),17-dien-16,19-olide and named as salvimirzacolide C.

**FIGURE 7:**
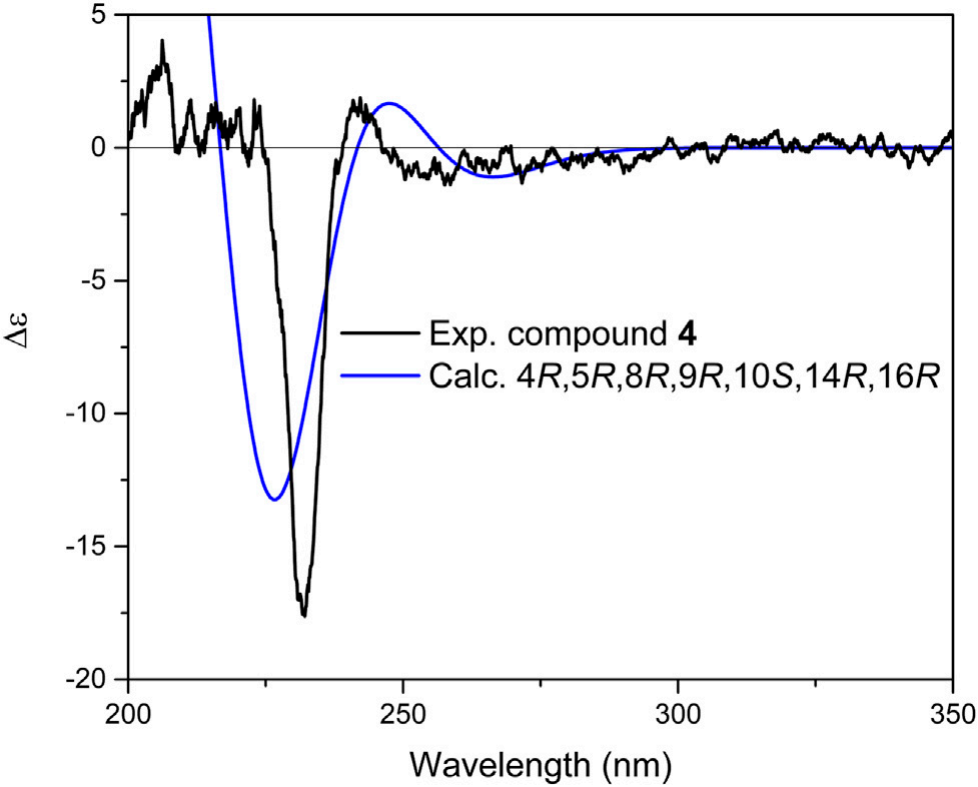
Comparison of experimental and calculated ECD spectra of compound **4** at TD-DFT/B3LYP/6-31+G(d,p) basis set and level of theory in acetonitrile.

Compound **4** was isolated as a colorless gum and indicated the same molecular formula as **3** (C_25_H_38_O_6_) based on its HRESIMS molecular ion peak at *m/z* 457.2557 [M+Na]^+^. Comprehensive analyses of the NMR spectra revealed that **4** possessed the identical planar structure as **3**. The NOSEY experiment was performed to clarify the relative configuration. Compound **4** showed diagnostic NOESY correlations between H-25, H-24, and H-22, as well as NOE between H-9 and H-5, suggesting a relative conﬁguration of 4*R* ^*^,5*R* ^*^,8*R* ^*^,9*R* ^*^,10*S* ^*^. Similar to **3**, the relative conﬁguration of **4** at centers C-14 and C-16 was established in a similar approach described for **3**, and only 14*R*, 16*R* stereoisomer (isomer 1) could be suggested for this compound. The results further supported by comparison of calculated total correlation coefficients for both isomers (0.99371 for isomer 1 and 0.99158 for isomer 2; Supplementary Figure S65). Further simulation of ECD spectrum and its comparison with experimental data displayed a great fit and resulted therefore in determining the absolute configuration of **4** as 4*R*, 5*R*, 8*R*, 9*R*, 10*S*, 14*R*, 16*R* (Figure 7). Thus, the structure of compound **4** was elucidated as (4*R*, 5*R*, 8*R*, 9*R*, 10*S*, 14*R*, 16*R*)-8,14-Dihydroxylabd-13 (21),17-dien-16,19-olide, and named as salvimirzacolide D.

**FIGURE 8:**
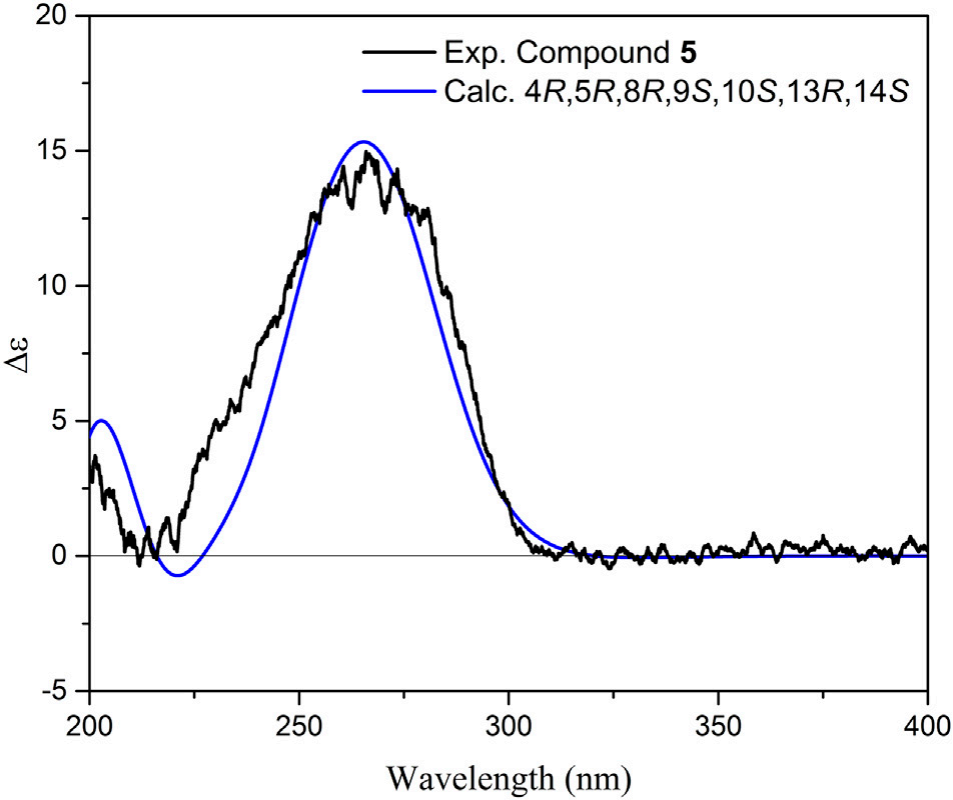
Comparison of experimental and calculated ECD spectra of compound **5** at TD-DFT/B3LYP/6-31+G(d,p) basis set and level of theory in acetonitrile.

Compound **5** was isolated as a colorless gum. Its molecular formula determined as C_25_H_36_O_6_ according to HRMS spectrum (*m/z* 455.2400 [M+Na]^+^, calcd. 455.2404). The molecular formula accounted for eight degrees of unsaturation. Based on ^13^C NMR and HSQC spectrum (Table 2), 25 resonances were assigned to five methyl groups, seven methylenes, five methines, and eight quaternary carbons. The ^13^C NMR data moreover showed signals for two double bonds (δ_C_ 110.9, 150.9 and 155.4, 117.2) and two carbonyl carbons (δ_C_ 169.4, 182.4). The absence of any other sp or sp^2^ carbon signal implied that the structure of **5** contained four rings. Three carbon resonances at δ_C_ 70.2, 75.8, and 79.6 indicated their connection to oxygen. The ^1^H NMR spectrum (Table 2) of **5** illustrated resonances of five methyl singlets at δ_H_ 0.81, 1.10, 1.17, 1.20, and 2.15, as well as two vinyl protons at *δ*_H_ 5.33 (*d*, 8.7 Hz) and 5.95 (*s*). A doublet at *δ*_H_ 4.81 (H-14, *J* = 8.7 Hz) indicated an oxygen-bearing carbon situated near the vinylic methine H-15 (*δ*_H_ 5.33 *d*, 8.7 Hz). This was further confirmed by the COSY correlation between them, and HMBC correlation from H-14 to C-16 and from H-15 to C-17, suggesting their connectivity to the lactone moiety. HMBC correlations between H-14 and C-12, C-13, and C-8 demonstrated the foundation of a seven-membered heterocyclic ring by the connection of C-14 to C-8 through the oxygen bridge. Additionally, HMBC correlations from H-21 to C-12, C-13, and C-14 confirmed its location on C-13 adjacent to a hydroxyl group. The remaining parts of the molecule (rings A and B) were similar to those of the above-mentioned molecules. According to the NOESY spectrum the relative configurations of rings A and B were identical to those in **1–4** (Figure 3). Moreover, the NOESY spectra displayed NOEs between H-9 and H-14 (confirming their co-facial orientation) and among H-21/H-22/H-25 (Figure 3). To decipher the AC of compound **5**, geometrical optimization and minimization, and subsequent ECD spectrum calculation of compound **5** were performed at CPCM/B3LYP/6-31+G(d,p)//6-31G(d) basis sets and level of theories. Overlaying calculated and experimental spectra resulted in establishing the absolute configuration of **5** as 4*R*, 5*R*, 8*R*, 9*R*, 10*S*, 13*S*, 14*R* (Figure 8). The structure of **5** therefore was determined as (4*R*,5*R*,8*R*,9*R*,10*S*,13*S*,14*R*)-8,14-Epoxy-13-hydroxylabd-15,17-dien-15(Z)-16,19-olide, and named as salvimirzacolide E. Compound **5** is the first example of a sesterterpene structure extracted from *Salvia* species bearing a heptagonal C-ring.

**TABLE 2:** ^1^H and ^13^C NMR spectroscopic data of compounds **5**–**7** (600 MHz, *δ* in ppm, *J* in Hz)

Position	5		6		7	
	δ_H_	δ_C_	δ_H_	δ_C_	δ_H_	δ_C_
1α	1.10, m	38.7	0.98, m	38.2	0.99, m	40.1
1β	1.66, m	—	1.67, m	—	1.40, m	—
2α	1.63, m	17.7	1.60, m	17.6	1.60, m	17.5
2β	1.57, m	—	1.60, m	—	1.53, m	—
3α	1.60, m	37.0	1.62, m	37.0	1.62, m	37.3
3β	1.75, m	—	1.77, m	—	1.75, m	—
4	—	47.4	—	47.2	—	47.23
5	1.76, m	50.4	1.77, m	50.6	1.78, m	49.8
6α	1.31, m	18.9	1.48, m	14.7	1.36, m	22.9
6β	1.52, m	—	1.61, m		1.49, m	—
7α	1.79, m	38.7	1.72, m	42.6	1.89, m	42.7
7β	1.55, m	—	1.38, m		1.59, m	—
8	—	79.6	—	75.8	—	72.6
9	1.59, m	58.4	1.16, m	58.4	2.04, d (10.62)	66.0
10	—	37.0	—	36.3	—	36.9
11a	1.40, m	23.4	1.41, m	22.5	6.82, dd (15.0,10.62)	143.6
11b	1.33, m	—	1.29, m	—	—	—
12a	1.39, m	46.3	1.46, m	30.3	6.23, d (15.0)	135.7
12b	1.90, m	—	1.69, m	—	—	—
13	—	75.8	—	75.6	—	197.5
14	4.81, d (8.7)	70.2	3.28, dd (9.54, 1.74)	72.7	—	—
15a	5.33, d (8.7)	110.9	2.06, m	32.4	—	—
15b	—	—	1.75, m		—	—
16	—	150.9	5.02, m	83.0	2.27, s	28.1
17	—	155.4	—	170.0	1.26, s	25.1
18	5.95, s	117.2	5.79, s	116.7	—	182.4
19	—	169.4	—	173.4	1.17, s	16.5
20	2.15, s	12.1	2.15, s	14.6	1.02, s	16.4
21	1.20, s	21.5	1.18, s	23.4	—	—
22	1.17, s	24.3	1.29, s	24.5	—	—
23	—	182.4	—	183.2	—	—
24	1.10, s	16.7	1.13, s	16.1	—	—
25	0.81, s	16.8	0.80, s	15.9	—	—

**FIGURE 9:**
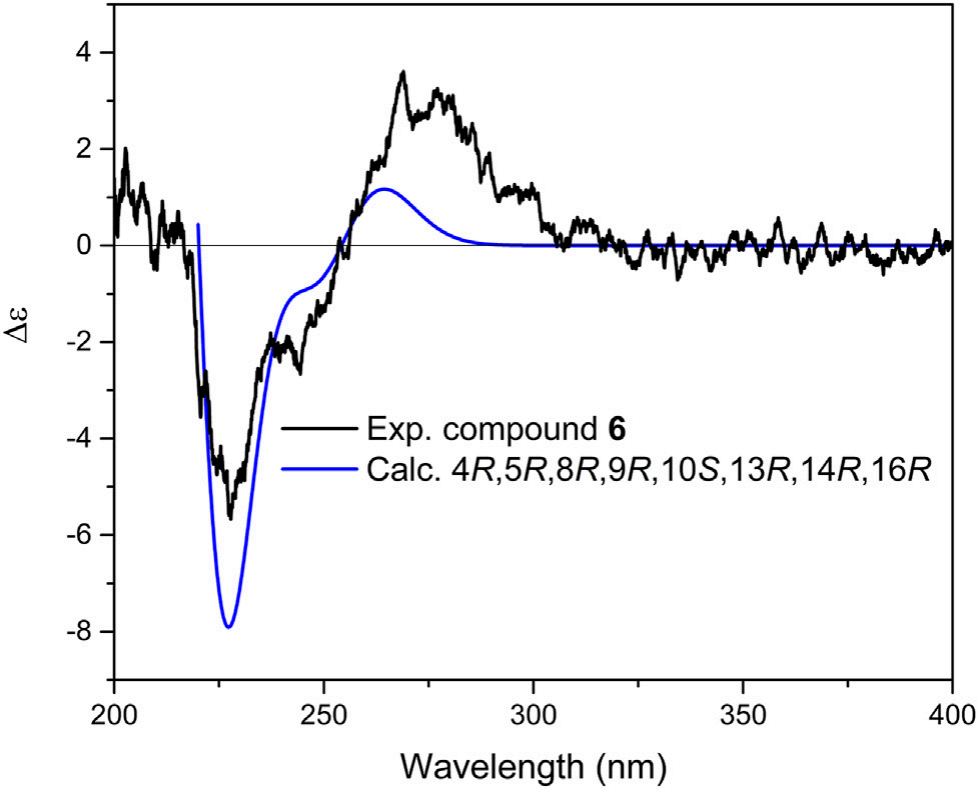
Comparison of experimental and calculated ECD spectra of compound **6** at TD-DFT/B3LYP/6-31+G(d,p) basis set and level of theory in acetonitrile.

Compound **6** was isolated as a whitish gum. Its molecular formula of C_25_H_38_O_6_ was derived from HRMS spectrum, displaying a pseudo-molecular ion peak at *m/z* 457.2579 [M+Na]^+^ (calcd. 457.2560). The molecular formula accounted for seven indices of hydrogen deﬁciency. The NMR data of **6** were similar to those of (4*R*, 5*R*, 8*R*, 9*R*, 10*S*, 13*S*, 16*R*)-14-hydroxymanoyloxide-17-en-16,19-olide, previously isolated by authors from the same plant (Ebrahimi et al., 2014). Notable diﬀerences were observed for resonances attributable to the C-9 and C-15 with the paramagnetically shifted signal of C-9 (*δ*_C_ 58.4) with Δ*δ* = +3.6 and diamagnetically shifted signal of C-15 (*δ*_C_ 32.4) with Δ*δ* = −4.5 (Table 2). Additionally, the resonances of H-9 (*δ*_H_ 1.16), H-14 (*δ*_H_ 3.28), and H-15a (*δ*_H_ 2.06) in ^1^H NMR spectrum were shifted (Δ*δ* = −0.34, −0.47, and +0.48 ppm, respectively). Careful inspection of the HMBC and ^1^H–^1^H COSY correlations revealed that compound **6** possessed the same planar structure as of 14-hydroxymanoyloxide-17-en-16,19-olide. According to these alterations in chemical shifts, it was assumed that both compounds should be stereoisomers. Consequently, since in the previous study the stereochemistry of the hydroxyl group C-14 was not identified, it can be assumed that the difference could be potentially related to the stereochemistry of C-13, C-14, and C-16. The NOESY spectrum displayed correlations between H-25, H-24, and H-22; H-22 and H-21; and between H-5 and H-9, corroborating the linkage of rings A, B, and C. Strong NOESY correlations between H-14 with H-21 and H-12β suggested that the predominate conformation of **6** is that having *gauche* interactions of H-14 with both C-21 and C-12 (Hammann et al., 1991; Suzuki et al., 2009; Jacobsen, 2017). Nevertheless, the final confirmation was simultaneously deduced from DP4+ probability calculation for two possible isomers, 14*R*, 16*R* and 14*S*, 16*R* (Supplementary Figure S66). The results obtained illustrated that the 14*R*, 16*R* with probability of 100% to be the correct stereoisomer. Further calculation of ECD spectrum led to the determination of the absolute configuration of **6** as 4*R*, 5*R*, 8*R*, 9*R*, 10*S*, 13*R*, 14*R*, 16*R* (Figure 9). In conclusion, the structure of **6** was deﬁned as (4*R*, 5*R*, 8*R*, 9*R*, 10*S*, 13*R*, 14*R*, 16*R*)-14-Hydroxymanoyloxide-17-en-16,19-olide, and named as salvimirzacolide F.

Compound **7** (Table 2) was isolated as a colorless gum. The HRESIMS of **7** showed a molecular ion at *m/z* 307.1914 [M+H]^+^ (calcd 307.1915), indicating a molecular formula of C_18_H_28_O_4_ and five indices of hydrogen deficiency. The ^13^C NMR spectrum showed 18 carbon resonances, which were assigned with the aid of the HSQC spectra as four methyl, five methylenes, four methines, and five quaternary carbons. The ^1^H NMR spectrum (Table 2) displayed the characteristic signals of four methyl singlets at *δ*_H_ 1.02, 1.17, 1.26, and 2.27, and two olefinic protons at *δ*_H_ 6.23 (*d*, 15.0 Hz) and 6.82 (*dd*, 15.0, 10.6 Hz). These data were reminiscent of a nor-diterpenoid scaffold. The ^1^H–^1^H COSY spectrum showed three discrete spin systems, including H_2_-1/H_2_-2/H_2_-3, H-5/H_2_-6/H_2_-7, and H-9/H-11/H-12. HMBC correlations originated from methyl groups, H-17, H-18, and H-15, confirmed the substructure of the rings A and B as those in the aforementioned sesterterpenoid systems. Finally, HMBC correlations from H-12 to C-9, and from H-11 and H-14 to C-13 completed the substructure of the side chain as *α*,*β*-unsaturated methyl ketone and its connection to C-9. NOESY correlations of H-18 (*δ*_H_ 1.02) with H-17 (*δ*_H_ 1.17) and H-15 (*δ*_H_ 1.26) indicated their co-facial orientation (Figure 3). Equally, an NOE cross peak between H-5 and H-9 corroborated the side chain to be, β -oriented. Hence, the structure of **7** was established as 8α-Hydroxy-11(E)-en-13-oxo-14,15-dinorlabdan-18-oic. Establishing the absolute configuration of this compound failed due to the lack of solubility of this compound in acetonitrile or methanol, and its minor quantity.

### 2.1 Investigation of Cytotoxic Activity of the Compounds 1-7

The antiproliferative activity of compounds **1-7** was evaluated on three human melanoma cancer cells: A375, WM164, and 541Lu. No significant inhibitory effects were observed at the concentration range used (up to 100 µM).

Salvia is one of the few genera in the Lamiaceae family that produces sesterterpenoids (Qingwen et al., 2021). To date, more than 130 species of *Salvia* have been phytochemically studied. Among them, sesterterpenes are reported only from 10 species including *S. hypoleuca*, *S. mirzayanii*, *S. Lachnocalyx*, *S. sahendica*, *S. limbata*, *S. dominica*, *S. yosgadensis*, *S. palaestina*, *S. syriaca,* and *S. aethiopis* (Rustaiyan and Sadjadi 1987; Gonzalez et al., 1989; Moghaddam et al., 1995; Linden et al., 1996; Topcu et al., 1996; Ulubelen et al., 1996; Cioffi et al., 2008; Dal Piaz et al., 2010; Hasan et al., 2016). It is noteworthy that all these species grow in the Middle East and eight of them are in flora of Iran. The reported sesterterpenoids are mainly labdane-type or their manoyloxide derivatives with a lactone moiety at the end of the chain. As a conclusion, six new sesterterpenoids together with one new nor-diterpenoid were isolated from aerial parts of *S. mirzayanii* and their absolute configuration was established by means of electronic circular dichroism spectroscopy. Assessing their biological activity revealed no potential cytotoxic activity of them.

## 3 Materials and Methods

### 3.1 General Experimental Procedures

Optical rotations were measured with a Jasco P-2000 polarimeter (Japan) using a 10.0 cm tube and the suitable solvent for each compound (MeOH or CHCl_3_). IR spectra were recorded on a Bruker ALPHA FT-IR apparatus equipped with a Platinum ATR module, and ECD experiments conducted on a J-1500 spectrophotometer (JASCO, Japan). One- and two-dimensional NMR experiments (for compounds **3–**7**)** were recorded on a Bruker Avance II 600 spectrometer (Bruker) operating at 600.19 MHz (^1^H) and 150.91 MHz (^13^C) at 300 K (chemical shifts δ in ppm, coupling constants *J* in Hz), with deuterated chloroform (chloroform-*d*) as the solvent, containing TMS 0.03%. The solvent was purchased from Euriso-top SAS (Saint-Aubin Cedex, France). NMR experiments for **1** and **2** were conducted on Bruker Avance III 500 spectrometer.

High-resolution mass spectra were measured with a Q-Exactive HF-X Orbitrap mass spectrometer (Thermo, United States) for compounds **3**-**7** and on micrOTOF-Q II (Bruker) for compounds **1** and **2**. Silica gel (70–230 and 230–400 mesh, Merck) was used for column chromatography. Preparative TLC was performed on silica gel 60 GF254 (Merck). Spots were detected on TLC under UV or by heating after spraying with 5% phosphomolibdic acid in ethanol. HPLC separations were performed on a Knauer HPLC system consisting of a mixing pump with degasser module, PDA detector, and an autosampler. Knauer Eurospher П 100–5 RP C18 (5 μm, 4.6 × 250 mm i. d.) and SunFire Prep C18 ODB (5 μm, 19 × 50 mm i. d.) columns were used for analytical and semi-preparative separations, respectively. Solvents used for extraction and column chromatography were of technical grade and were distilled before use. HPLC grade solvents (Merck) were used for HPLC.

### 3.2 Plant Material

The aerial parts of *S. mirzayanii* were collected at the flowering stage in March 2015 from Geno mountain (E 56°.09′.66″, N 27°.23′.01″) Hormozgan, Iran, and identiﬁed by Dr. M. A. Soltanipoor. A voucher specimen (No. 5322) was deposited at the herbarium of the Hormozgan Agricultural and Natural Resource Research Center, Bandarabbas, Iran. Plant materials were shade-dried and stored properly until extraction.

### 3.3 Extraction and Isolation

The air-dried and powdered aerial parts of *S. mirzayanii* (5.0 kg) were extracted successively with *n*-hexane (3 × 20 L) and acetone (5 × 20 L) by maceration at room temperature. Extracts were concentrated in *vacuo* to afford dark gummy residues of *n*-hexane (120 g) and acetone (110 g) extracts. The acetone extract was subjected to chromatography on a silica gel column (230–400 mesh, 900 g) and eluted with a gradient of *n*-hexane−EtOAc (100:0 to 0:100, v/v) followed by increasing concentrations of MeOH (up to 25%). Fractions of 250 mL were collected and after screening by TLC, fractions with similar compositions were pooled to yield 28 combined fractions.

Fractions 17 and 18 were combined [3.8 g, eluted with *n*-hexane−EtOAc (50:50)] and subjected to silica gel column chromatography (70–230 mesh, 230 g), eluted with a gradient of CHCl_3_−acetone (90:10), to afford four fractions (18b, 18h, 18i, and 18k). Fraction 18k (25.0 mg) was dissolved in 0.5 mL MeOH and separated by preparative RP-HPLC (SunFire C18, 5 μm, 10 × 150.0 mm i. d.; Waters) with a step gradient consisting of H_2_O and 0.1% Formic acid (solvent A) and MeCN (solvent B) as follows: 40% B for (0–5 min), 40–50% B (5–12 min), 50% B (12–18 min), 50–60% B (18–30 min). The flow rate was 20 mL/min. Fraction 18k aﬀorded **1** [1.5 mg, *t*_R_ = 14.0 min, >95% purity (^1^H NMR)] and **2** [0.5 mg, *t*_R_ = 15.1 min, >95% purity (^1^H NMR)]. Fraction 21 (200 mg) was puriﬁed by semipreparative RP-HPLC [H_2_O (A), MeOH (B); 55% B (0–6 min), 55–97% B (6–60 min); ﬂow rate 4 mL/min; sample concentration 100 mg/mL in DMSO; injection volume 100 µL] to yield compounds **5** (1.5 mg, *t*_R_ = 40.3 min), **6** (1 mg, *t*_R_ = 42.1 min). Fraction 23 (4.0 g) was further puriﬁed by silica gel column chromatography (44 × 3.0 cm, 70–230 mesh) with CHCl_3_−MeOH (95:5 to 30:70, v/v) to aﬀord five subfractions (F_23_a to F_23_e). F_23_e (120 mg) was further puriﬁed by semipreparative RP-HPLC [H_2_O (A), MeCN (B); 30% B (0–5 min), 30–65% B (5–60 min); ﬂow rate 4 mL/min; sample concentration 120 mg/mL in DMSO; injection volume 100 μL] to aﬀord compound **7** (1.5 mg, *t*_R_ = 13.1 min). Fraction 24 (5.35 g) was puriﬁed by silica gel column chromatography (61 × 3.5 cm, 70–230 mesh) with CHCl_3_−Acetone (98:2 to 30:70, v/v) to aﬀord seven subfractions (F_24_l to F_24_r). F_24_r (200 mg) was puriﬁed by semipreparative RP-HPLC [H_2_O (A), MeCN (B); 30% B (0–5 min), 30–60% B (5–60 min); ﬂow rate 4 mL/min; sample concentration 200 mg/mL in DMSO; injection volume 100 µL] to yield compounds **3** (1 mg, *t*_R_ = 21.6 min) and **4** (2 mg, *t*_R_ = 23.4 min).

### 3.4 Compounds Characterization

Salvimirzacolide A (compound **1**): C_25_H_36_O_5;_ colorless gum; ${\left[\text{α}\right]}_{\text{D}}^{20}$ –20.15 (*c* 0.022, CHCl_3_); ECD (*c* 100 × 10^–6^ M, ACN) λ_max_ (Δε) 320 (+2.5); for ^1^H NMR data (500 MHz, Methanol-*d*_4_) and ^13^C (125 MHz, Methanol-*d*_4_) spectroscopic data see Table 1; HRESIMS *m/z* 831.5056 [calcd for C_50_H_71_O_10_, [2M−H]^−^, *m/z* 831.5053].

Salvimirzacolide B (compound **2**): C_25_H_36_O_5;_ colorless gum; ${\left[\text{α}\right]}_{\text{D}}^{20}$ –17.5 (*c* 0.018, MeOH); ECD (*c* 100 × 10^–6^ M, ACN) λ_max_ (Δε) 300 (+1.6); for ^1^H NMR data (500 MHz, Methanol-*d*_4_) and ^13^C (125 MHz, Methanol-*d*_4_) spectroscopic data see Table 1; HRESIMS *m/z* 831.5056 [calcd for C_50_H_71_O_10_ [2M−H]^−^, *m/z* 831.5053].

Salvimirzacolide C (compound **3**): C_25_H_38_O_6;_ colorless gum; ${\left[\text{α}\right]}_{\text{D}}^{20}$ + 21.32 (*c* 0.017, CHCl_3_); UV_max_ (ACN) λ_max_ (log ε) 270 (2.1), 218 (2.95) nm; ECD (*c* 100 × 10^–6^ M, ACN) λ_max_ (Δε) 232 (−22.58); IR ν_max_ 2,932, 1,727, 1,643 cm^−1^; for 1H NMR data (600 MHz, Chloroform-*d*_1_) and 13C (150 MHz, Chloroform-*d*_1_) spectroscopic data see Table 1; HRESIMS *m/z* 457.2557 [calcd for C_25_H_39_O_6_ [M+H]^+^, *m/z* 457.2560].

Salvimirzacolide D (compound **4**): C_25_H_38_O_6;_ colorless gum; ${\left[\text{α}\right]}_{\text{D}}^{20}$ + 43.9 (*c* 0.012, CHCl_3_); UV_max_ (ACN) λ_max_ (2.15), 218 (2.90) nm; ECD (*c* 100 × 10^–6^ M, ACN) λ_max_ (Δε) 243 (+16.61), 231 (−17); IR *ν*_max_ 2,929, 1,728, 1,642 cm^−1^; for ^1^H NMR data (600 MHz, Chloroform-*d*_1_) and ^13^C (150 MHz, Chloroform-*d*_1_) spectroscopic data see Table 1; HRESIMS *m/z* 457.2557 (calcd for C_25_H_38_O_6_Na [M+Na]^+^, *m/z* 457.2560).

Salvimirzacolide E (compound **5**): C_25_H_36_O_6;_ colorless gum; ${\left[\text{α}\right]}_{\text{D}}^{20}$ + 26.51 (*c* 0.033, CHCl_3_); UV_max_ (ACN) λ_max_ (log ε) 270 (1.8), 200 (2.85) nm; ECD (*c* 100 × 10^–6^ M, ACN) λ_max_ (Δε) 268 (+14.13); IR *ν*_max_ 2,930, 1,719 cm^−1^; for ^1^H NMR data (600 MHz, Chloroform-*d*_1_) and ^13^C (150 MHz, Chloroform-*d*_1_) spectroscopic data see Table 2; HRESIMS: *m/z* 455.2400 [calcd. for C_25_H_36_O_6_Na [M+Na]^+^, *m/z* 455.2404].

Salvimirzacolide F (compound **6**): C_25_H_38_O_6;_ colorless gum; ${\left[\text{α}\right]}_{\text{D}}^{20}$ + 41.18 (*c* 0.016, CHCl_3_); UV_max_ (ACN) λ_max_ (log ε) 270 (2.0), 210 (2.78) nm; ECD (*c* 100 × 10^–6^ M, ACN) λ_max_ (Δε) 277 (+3.22), 245 (−2.097), 228 (−5.35); IR ν_max_ 2,931, 1,728 cm^−1^; for ^1^H NMR data (600 MHz, Chloroform-*d*) and ^13^C (150 MHz, Chloroform-*d*_1_) spectroscopic data see Table 2; HRESIMS *m/z* 457.2579 [calcd for C_25_H_38_O_6_Na [M+Na]^+^, *m/z* 457.2560].

Salvimirzacolide G (compound **7**): C_18_H_28_O_4;_ colorless gum; ${\left[\text{α}\right]}_{\text{D}}^{20}$ –12.67 (*c* 0.021, CHCl_3_); UV_max_ (ACN) λ_max_ not available; ECD (ACN) λ_max_ (Δε) not available; IR ν_max_ 2,929, 1,698, 1,249 cm^−1^; for ^1^H NMR data (600 MHz, Chloroform-*d*_1_) and ^13^C (150 MHz, Chloroform-*d*_1_) spectroscopic data see Table 2; HRESIMS *m/z* 307.1914 [calcd for C_25_H_40_O_6_ [M+H]^+^, *m/z* 307.1915].

### 3.5 Computational Methods

The 3D structure of selected compounds was subjected to Schrödinger MacroModel 9.1 (Schrödinger. LLC, United States) to perform conformational analysis using the OPLS-3 force ﬁeld in the gas phase and mixed tortional/low-mode sampling method. The number of steps was considered high enough to include all important conformers. Conformers occurring in the energy window of 5 kcal.mol^−1^ were subjected to geometrical optimization and frequency calculation using DFT/B3LYP/6-31G(d) in the gas phase with Gaussian 16 (Frisch et al., 2016). No imaginary frequencies were observed. To perform DP4+ probability calculation, the optimized conformers with population of more than 1% were subjected to NMR shielding tensors calculation (^13^C and ^1^H) using Gauge-Independent Atomic Orbital (GIAO) method at mPW1PW91/6–31+G(d,p)/CPCM basis set and level of theory in chloroform. Similar calculation was performed for TMS. The values obtained were converted to unscaled chemical shifts using *unscaled chemical shift* = σ_TMS_–σ_x_; where σ_x_ is the calculated shielding tensor for atom x. DP4+ probability calculation was ultimately conducted by using the excel sheet provided by Grimbalt et al. (2015) using only the atoms from side chains which are the most atoms configurational changes. Calculation of excitation energy (nm), rotatory strength, dipole velocity (R_*vel*_), and dipole length (R_*len*_) were performed by TD-DFT/B3LYP/6-31G(d,p)/CPCM/acetonitrile for compounds **1** and **2**, and were performed by TD-DFT/B3LYP/6-31+G(d,p)/CPCM/acetonitrile for compounds **3**–**7**. All calculated spectra were Boltzmann-averaged and ECD curves were extracted by SpecDis v.1.7 software (Bruhn et al., 2017) with a half-band of 0.2–0.3 eV. UV shift of ±10 nm was applied prior to comparison to experimental ECD spectra.

### 3.6 Cell Culture and Cell Cytotoxicity Assay

#### 3.6.1 Culture Conditions

The human melanoma cell lines A375, WM164, and 541Lu were grown in Dulbecco’s modified Eagle medium (DMEM) supplemented with 5% FBS, 1% PS, and 1% L-glutamine. All cells were passaged routinely by trypsinization until they attained confluence and were maintained in a humidified 5% CO_2_ atmosphere at 37°C.

#### 3.6.2 Cytotoxicity Assay

The cytotoxicity assay was performed as described before (Alilou, et al., 2020) using cell counting kit-8 (WST-8, ABCAM). Briefly, cells were seeded in 96-well plates at a density of 1 × 10^4^ per well and incubated in fresh medium at 37°C for 24 h. After 24 h incubation, compounds **1**-**7** were added to cells in five different concentrations (100, 75, 50, 25, and 5 µM). After 24 h of incubation, media were removed from wells and subsequently 100 µL of 10% WST-8 solution in DMEM was added and the plate was incubated for further 3 h. Then, the absorbance of the wells was measured at 450 nm using an Elisa Plate Reader (Tecan Infinite F200) and the activity calculated as percentage cell viability.

## Data Availability

The original contributions presented in the study are included in the article/Supplementary Material. Further inquiries can be directed to the corresponding author.

## References

[B1] Bruhn T. Schaumlöffel A. Hemberger Y. Pecitelli G. (2017). SpecDis Version 1.71. Berlin, Germany. Availableat: https:/specdis-software.jimdo.com .

[B2] Cabanillas A. H. Tena Pérez V. Maderuelo Corral S. Rosero Valencia D. F. Martel Quintana A. Ortega Doménech M. (2018). Cybastacines A and B: Antibiotic Sesterterpenes from a Nostoc Sp. Cyanobacterium. J. Nat. Prod. 81 (2), 410–413. 10.1021/acs.jnatprod.7b00638 29432010

[B3] Chen Q. Li J. Ma Y. Yuan W. Zhang P. Wang G. (2021). Occurrence and Biosynthesis of Plant Sesterterpenes (C25), a New Addition to Terpene Diversity. Plant Commun. 2 (5), 100184. 10.1016/j.xplc.2021.100184 34746758 PMC8553974

[B4] Cioffi G. Bader A. Malafronte A. Dal Piaz F. De Tommasi N. (2008). Secondary Metabolites from the Aerial Parts of Salvia Palaestina Bentham. Phytochemistry 69 (4), 1005–1012. 10.1016/j.phytochem.2007.11.002 18191162

[B5] Dal Piaz F. Imparato S. Lepore L. Bader A. De Tommasi N. (2010). A Fast and Efficient LC-MS/MS Method for Detection, Identification and Quantitative Analysis of Bioactive Sesterterpenes in Salvia dominica Crude Extracts. J. Pharm. Biomed. Anal. 51 (1), 70–77. 10.1016/j.jpba.2009.08.006 19720491

[B6] Ebrahimi S. N. Farimani M. M. Mirzania F. Soltanipoor M. A. De Mieri M. Hamburger M. (2014). Manoyloxide Sesterterpenoids from Salvia Mirzayanii. J. Nat. Prod. 77 (4), 848–854. 10.1021/np400948n 24689905

[B20] Farimani M. M. Mazarei Z. (2014). Sesterterpenoids and Other Constituents from Salvia Lachnocalyx Hedge. Fitoterapia 98, 234–240. 10.1016/j.fitote.2014.08.009 25128428

[B7] Farimani M. M. Taleghani A. Aliabadi A. Aliahmadi A. Esmaeili M. Namazi Sarvestani N. (2016). Labdane Diterpenoids from Salvia Leriifolia: Absolute Configuration, Antimicrobial and Cytotoxic Activities. Planta Med. 82 (14), 1279–1285. 10.1055/s-0042-107798 27280932

[B8] Frisch M. J. Trucks G. W. Schlegel H. B. Scuseria G. E. Robb M. A. Cheeseman J. R. (2016). Gaussian 09, Revision D.01. Wallingford CT: Gaussian, Inc.

[B9] Gonzalez M. S. San Segundo J. M. Grande M. C. Medarde M. Bellido I. S. (1989). Sesterterpene Lactones from Salvia Aethiopis. Salviaethiopisolide and 13-Epi-Salviaethiopisolide. Tetrahedron 45 (11), 3575–3582. 10.1016/S0040-4020(01)81036-X

[B10] Grimblat N. Zanardi M. M. Sarotti A. M. (2015). Beyond DP4: An Improved Probability for the Stereochemical Assignment of Isomeric Compounds Using Quantum Chemical Calculations of NMR Shifts. J. Org. Chem. 80 (24), 12526–12534. 10.1021/acs.joc.5b02396 26580165

[B11] Hammann P. E. Kluge H. Habermehl G. G. (1991). γ-Gauche Effects in the ^1^H and ^13^C NMR Spectra of Steroids. II. Magn. Reson. Chem. 29 (2), 133–136. 10.1002/mrc.1260290207

[B12] Hasan M. R. Al-Jaber H. I. Al-Qudah M. A. Abu Zarga M. H. (2016). New Sesterterpenoids and Other Constituents from Salvia dominica Growing Wild in Jordan. Phytochemistry Lett. 16, 12–17. 10.1016/j.phytol.2016.02.009

[B13] Jacobsen N. E. (2017). NMR Data Interpretation Explained; Understanding 1D and 2D NMR Spectra of Organic Compounds and Natural Products. Wiley & Sons.

[B14] Linden A. Juch M. Matloubi Moghaddam F. Zaynizadeh B. Rüedi P. (1996). The Absolute Configuration of Salvileucolide Methyl Ester, a Sesterterpene from Iranian Salvia Species. Phytochemistry 41 (2), 589–590. 10.1016/0031-9422(95)00655-9

[B15] Liu Y. Wang L. Jung J. H. Zhang S. (2007). Sesterterpenoids. Nat. Prod. Rep. 24 (6), 1401–1429. 10.1039/B617259H 18033586

[B16] Máximoa P. Lourenço A. (2018). Marine Sesterterpenes: An Overview. Coc 22 (24), 2381–2393. 10.2174/1385272822666181029101212

[B17] Moghaddam F. M. Amiri R. Alam M. Hossain M. B. van der Helm D. (1998). Structure and Absolute Stereochemistry of Salvimirzacolide, a New Sesterterpene from Salvia Mirzayanii. J. Nat. Prod. 61 (2), 279–281. 10.1021/np970378j 9514012

[B18] Moghaddam F. M. Farimani M. M. Seirafi M. Taheri S. Khavasi H. R. Sendker J. (2010). Sesterterpenoids and Other Constituents of Salvia Sahendica. J. Nat. Prod. 73 (9), 1601–1605. 10.1021/np1002516 20735065

[B19] Moghaddam F. M. Zaynizadeh B. Rüedi P. (1995). Salvileucolide Methylester, a Sesterterpene from Salvia Sahendica. Phytochemistry 39 (3), 715–716. 10.1016/0031-9422(95)00027-5

[B21] Rustaiyan A. Masoudi S. Tabatabaei-Anaraki M. (2007). Terpenoids from Iranian Salvia Species. Nat. Prod.Commun. 2 (10), 1934578X0700201012. 10.1177/1934578x0700201012

[B22] Rustaiyan A. Sadjadi A. (1987). Salvisyriacolide, a Sesterterpene from Salvia Syriaca. Phytochemistry 26 (11), 3078–3079. 10.1016/S0031-9422(00)84601-4

[B23] Soltanipour M. (2007). Investigation on Relationship between Ecological Factors and Natural Distribiution and Density of *Salvia Mirzayanii* Medicinal Species in Hormozgan Province. Iran. J. Med. Aroma. Plants. 23, 218–225.

[B24] Suzuki S. Horii F. Kurosu H. (2009). Theoretical Investigations of the γ-gauche Effect on the 13C Chemical Shifts Produced by Oxygen Atoms at the γ Position by Quantum Chemistry Calculations. J. Mol. Struct. 919 (1–3), 290–294. 10.1016/j.molstruc.2008.09.019

[B25] Tabefam M. Moridi Farimani M. Danton O. Ramseyer J. Nejad Ebrahimi S. Neuburger M. (2018). Antiprotozoal Isoprenoids from Salvia Hydrangea. J. Nat. Prod. 81, 2682–2691. 10.1021/acs.jnatprod.8b00498 30565934

[B26] Topcu G. Ulubelen A. Tam T. C.-M. Chun Tao-Che C. (1996). Sesterterpenes and Other Constituents of Salvia Yosgadensis. Phytochemistry 42 (4), 1089–1092. 10.1016/0031-9422(96)00041-6

[B27] Ulubelen A. Topcu G. Sönmez U. Eriş C. Özgen U. (1996). Norsesterterpenes and Diterpenes from the Aerial Parts of Salvia Limbata. Phytochemistry 43 (2), 431–434. 10.1016/0031-9422(96)00248-8

