# Supplementary material for: New Sesterterpenoids from Salvia mirzayanii Rech.f. and Esfand. Stereochemical Characterization by Computational Electronic Circular Dichroism
Source: Front Chem. 2022 Jan 20;9:783292. doi: 10.3389/fchem.2021.783292 (PMC8810545; doi:10.3389/fchem.2021.783292)
Supplement: Supplementary file 1 [file DataSheet1.pdf]

Supplementary Material

Table of Contents

Figure S1. ^{1}H NMR spectrum of compound 1 (500MHz, $CDCl_3$ + CD_3OD)..4
Figure S2. Expand ^{1}H NMR spectrum of compound 1 (500 MHz, $CDCl_3$ + CD_3OD).........................4
Figure S3. ^{13}C NMR (DEPTq) spectrum of compound 1 (500 MHz, $CDCl_3$ + CD_3OD)......................5
Figure S4. COSY spectrum of compound 1 (500 MHz, $CDCl_3$ + CD_3OD) 5
Figure S5. HSQC-DEPT spectrum of compound 1 (500 MHz, $CDCl_3$ + CD_3OD) 6
Figure S6. Expand of HSQC-DEPT spectrum of compound 1 (500 MHz, $CDCl_3$ + CD_3OD).............6
Figure S7. HMBC spectrum of compound 1 (500 MHz, $CDCl_3$ + CD_3OD) 7
Figure S8. Expand of HMBC spectrum of compound 1 (500 MHz, $CDCl_3$ + CD_3OD)........................7
Figure S9. NOESY spectrum of compound 1 (500 MHz, $CDCl_3$ + CD_3OD).......................................8
Figure S10. HRESIMS chromatogram of compound 1..8
Figure S11. ^{1}H NMR spectrum of compound 2 (500 MHz, $CDCl_3$ + CD_3OD).....................................9
Figure S12. Expand of ^{1}H NMR spectrum of compound 2 (500 MHz, $CDCl_3$ + CD_3OD) 9
Figure S13. ^{13}C NMR (DEPTq) spectrum of compound 2 (500 MHz, $CDCl_3$)..................................10
Figure S14. COSY spectrum of compound 2 (500 MHz, $CDCl_3$ + CD_3OD) 10
Figure S15. Expand of COSY spectrum of compound 2 (500 MHz, $CDCl_3$ + CD_3OD).....................11
Figure S16. HSQC-DEPT spectrum of compound 2 (500 MHz, $CDCl_3$ + CD_3OD) 11
Figure S17. HMBC spectrum of compound 2 (500 MHz, $CDCl_3$ + CD_3OD) 12
Figure S18. NOESY spectrum of compound 2 (500 MHz, $CDCl_3$ + CD_3OD)....................................12
Figure S19. HRESIMS chromatogram of compound 2...13
Figure S20. ^{1}H NMR spectrum of compound 3 (600 MHz, $CDCl_3$)..13
Figure S21. ^{13}C NMR spectrum of compound 3 (600 MHz, $CDCl_3$)...14
Figure S22. NOESY spectrum of compound 3 (600 MHz, $CDCl_3$)..14
Figure S23. HSQC spectrum of compound 3 (600 MHz, $CDCl_3$) 15
Figure S24. HMBC spectrum of compound 3 (600 MHz, $CDCl_3$) 15
Figure S25. NOESY spectrum of compound 3 (600 MHz, $CDCl_3$)..16
Figure S26. IR spectrum of compound 3...16
Figure S27. HRESIMS spectrum of compound 3 17
Figure S28. ^{1}H NMR spectrum of compound 4 (600 MHz, $CDCl_3$)..17
Figure S29. ^{13}C NMR spectrum of compound 4 (600 MHz, $CDCl_3$)...18
Figure S30. COSY spectrum of compound 4 (600 MHz, $CDCl_3$) 18
Figure S31. HSQC spectrum of compound 4 (600 MHz, $CDCl_3$) 19
Figure S32. HMBC spectrum of compound 4 (600 MHz, $CDCl_3$) 19

Figure S33. NOESY spectrum of compound 4 (600 MHz, $CDCl_3$)..20
Figure S34. IR spectrum of compound 4..20
Figure S35. HRESIMS chromatogram of compound 4..21
Figure S36. ^{1}H NMR spectrum of compound 5 (600 MHz, $CDCl_3$)....................................21
Figure S37. ^{13}C NMR spectrum of compound 5 (600 MHz, $CDCl_3$)...................................22
Figure S38. COSY spectrum of compound 5 (600 MHz, $CDCl_3$) 22
Figure S39. HSQC spectrum of compound 5 (600 MHz, $CDCl_3$) 23
Figure S40. HMBC spectrum of compound 5 (600 MHz, $CDCl_3$) 23
Figure S41. NOESY spectrum of compound 5 (600 MHz, $CDCl_3$)......................................24
Figure S42. IR spectrum of compound 5..24
Figure S43. HRESIMS chromatogram of compound 5..25
Figure S44. ^{1}H NMR spectrum of compound 6 (600 MHz, $CDCl_3$)....................................25
Figure S45. ^{13}C NMR spectrum of compound 6 (600 MHz, $CDCl_3$)...................................26
Figure S46. COSY spectrum of compound 6 (600 MHz, $CDCl_3$) 26
Figure S47. HSQC spectrum of compound 6 (600 MHz, $CDCl_3$) 27
Figure S48. HMBC spectrum of compound 6 (600 MHz, $CDCl_3$) 27
Figure S49. NOESY spectrum of compound 6 (600 MHz, $CDCl_3$)......................................28
Figure S50. IR spectrum of compound 6..28
Figure S51. HRESIMS chromatogram of compound 6..29
Figure S52. ^{1}H NMR spectrum of compound 7 (600 MHz, $CDCl_3$)....................................29
Figure S53. Expand of ^{1}H NMR spectrum of compound 7 (600 MHz, $CDCl_3$) 30
Figure S54. ^{13}C NMR spectrum of compound 7 (600 MHz, $CDCl_3$)...................................30
Figure S55. COSY spectrum of compound 7 (600 MHz, $CDCl_3$) 31
Figure S56. HSQC-DEPT spectrum of compound 7 (600 MHz, $CDCl_3$) 31
Figure S57. HMBC spectrum of compound 7 (600 MHz, $CDCl_3$) 32
Figure S58. NOESY spectrum of compound 7 (600 MHz, $CDCl_3$)......................................32
Figure S59. Expand of NOESY spectrum of compound 7 (600 MHz, $CDCl_3$) 33
Figure S60. IR spectrum of compound 7..33
Figure S61. HRESIMS chromatogram of compound 7..34
Table 1. Cartesian coordinates for the low-energy optimized conformer of 1 at B3LYP/6-31G (d,p) level of theory in MeOH..34
Table 2. Cartesian coordinates for the low-energy optimized conformer of 2 at B3LYP/6-31G (d, p) level of theory in MeOH..36
Table 3. Cartesian coordinates for the low-energy optimized conformer of 3 at B3LYP/6-31G (d) level of theory in gas phase 37
Table 4. Cartesian coordinates for the low-energy optimized conformer of 4 at B3LYP/6-31G (d) level of theory in gas phase 39

Table 5. Cartesian coordinates for the low-energy optimized conformer of 5 at B3LYP/6-31G (d) level of theory in gas phase 41

Table 6. Cartesian coordinates for the low-energy optimized conformer of 6 at B3LYP/6-31G (d) level of theory in gas phase 42

Figure S62. Result of DP4+ NMR chemical shift probability calculation for compound 3. Isomer 1 is: 4*R*,5*R*,8*R*,9*R*,10*S*,14*R*,16*R* Isomer 2 is: 4*R*,5*R*,8*R*,9*R*,10*S*,14*S*,16*R* 44

Figure S63. Graphs of calculated chemical shifts versus experimental chemicals shifts and ^{1}H, ^{13}C, and total correlation coefficients for two possible isomers of compound 3. 45

Figure S64. Result of DP4+ NMR chemical shift probability calculation for compound 4. Isomer 1 is: 4*R*,5*R*,8*R*,9*R*,10*S*,13*R*,14*R*,16*R* Isomer 2 is: 4*R*,5*R*,8*R*,9*R*,10*S*,13*R*,14*S*,16*R*.....................................45

Figure S65. Graphs of calculated chemical shifts versus experimental chemicals shifts and ^{1}H, ^{13}C, and total correlation coefficients for two possible isomers of compound 4. 46

Figure S66. Result of DP4+ NMR chemical shift probability calculation for compound 6. Isomer 1 is: 4*R*,5*R*,8*R*,9*R*,10*S*,13*R*,14*S*,16*R* Isomer 2 is: 4*R*,5*R*,8*R*,9*R*,10*S*,13*R*,14*R*,16*R*.....................................46

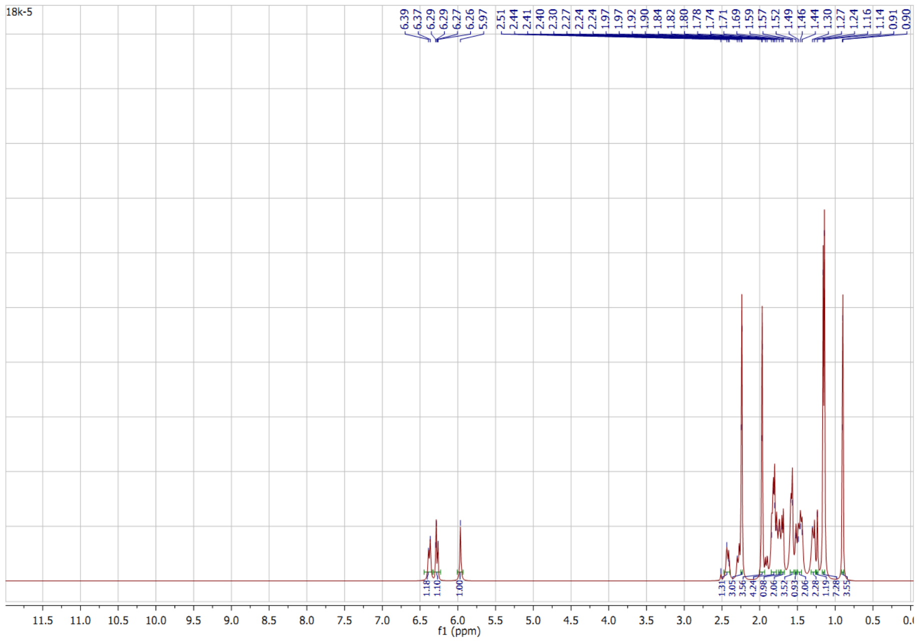

Figure S1. ^{1}H NMR spectrum of compound **1** (500MHz, $CDCl_3$ + CD_3OD)

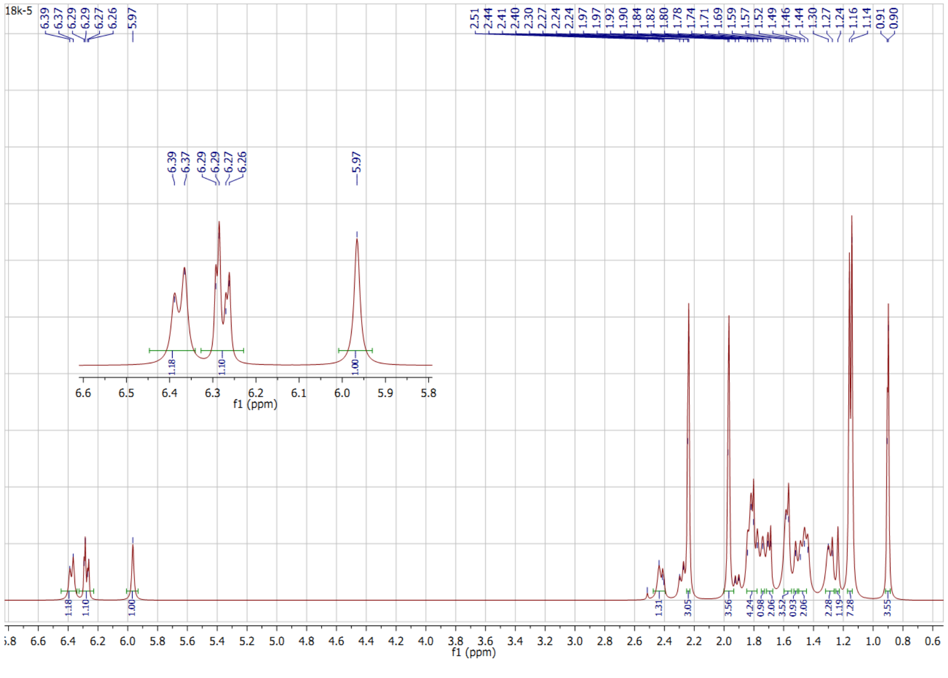

Figure S2. Expand ^{1}H NMR spectrum of compound **1** (500 MHz, $CDCl_3$ + CD_3OD)

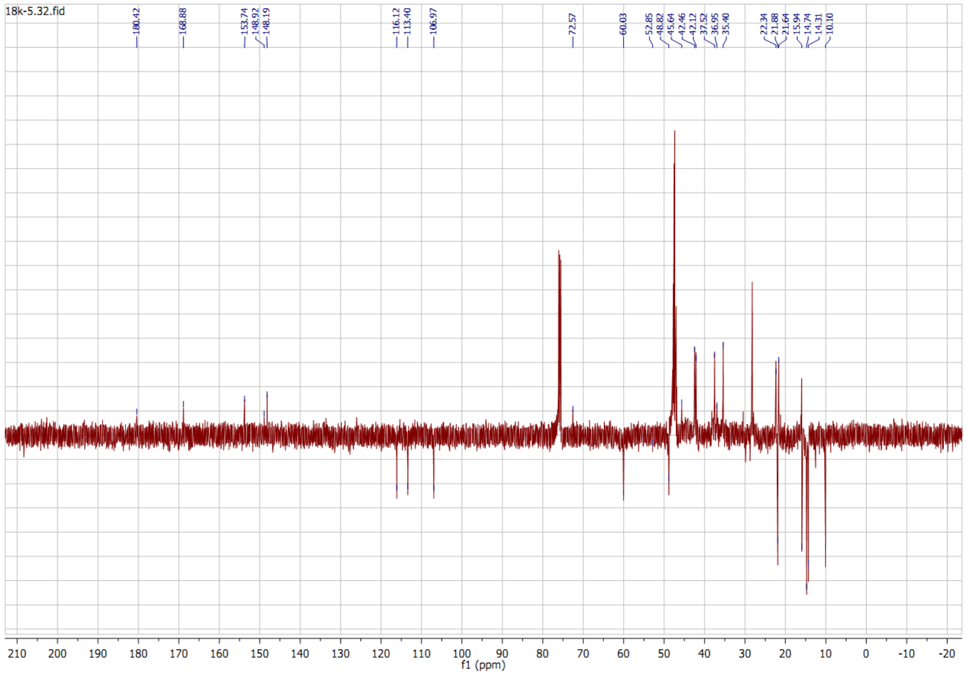

Figure S3. ^{13}C NMR (DEPTq) spectrum of compound **1** (500 MHz, $CDCl_3$ + CD_3OD)

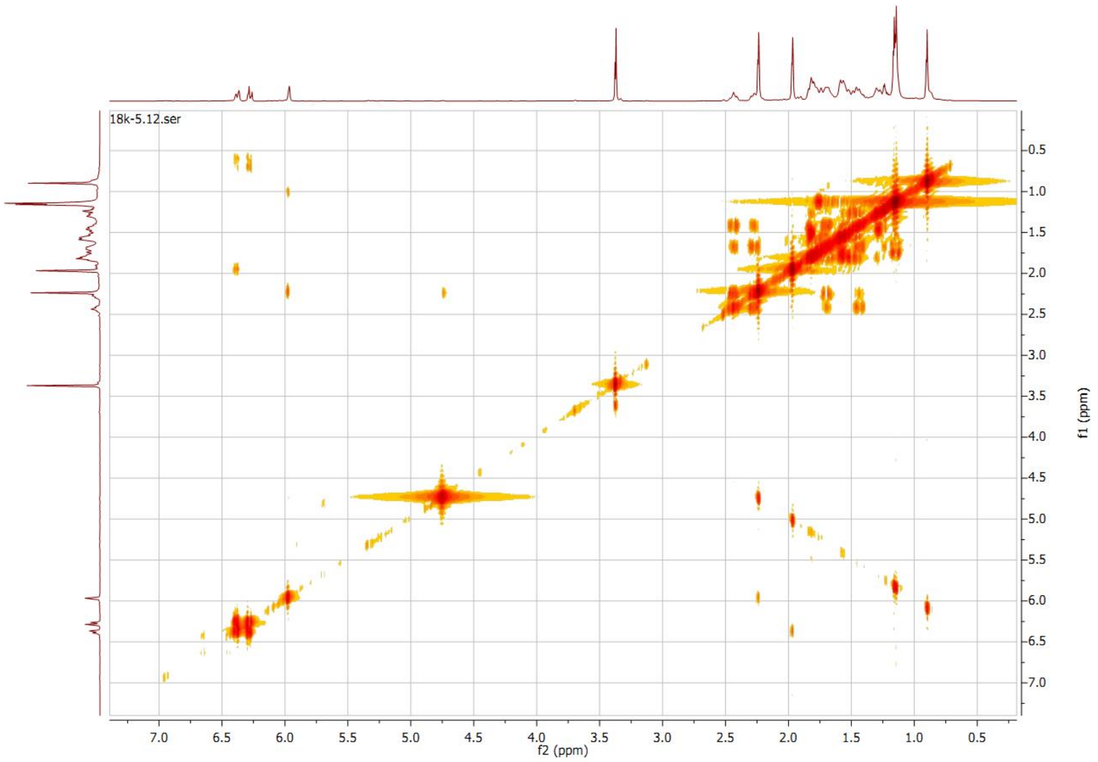

Figure S4. COSY spectrum of compound **1** (500 MHz, $CDCl_3$ + CD_3OD)

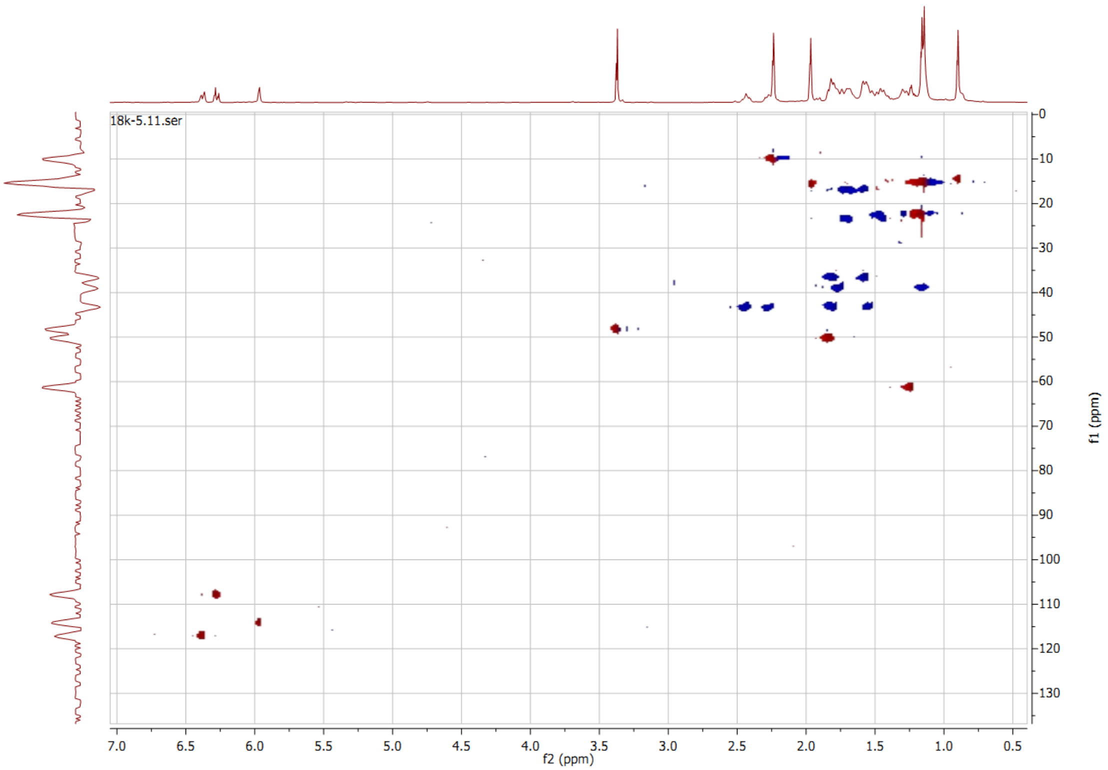

Figure S5. HSQC-DEPT spectrum of compound **1** (500 MHz, $CDCl_3$ + CD_3OD)

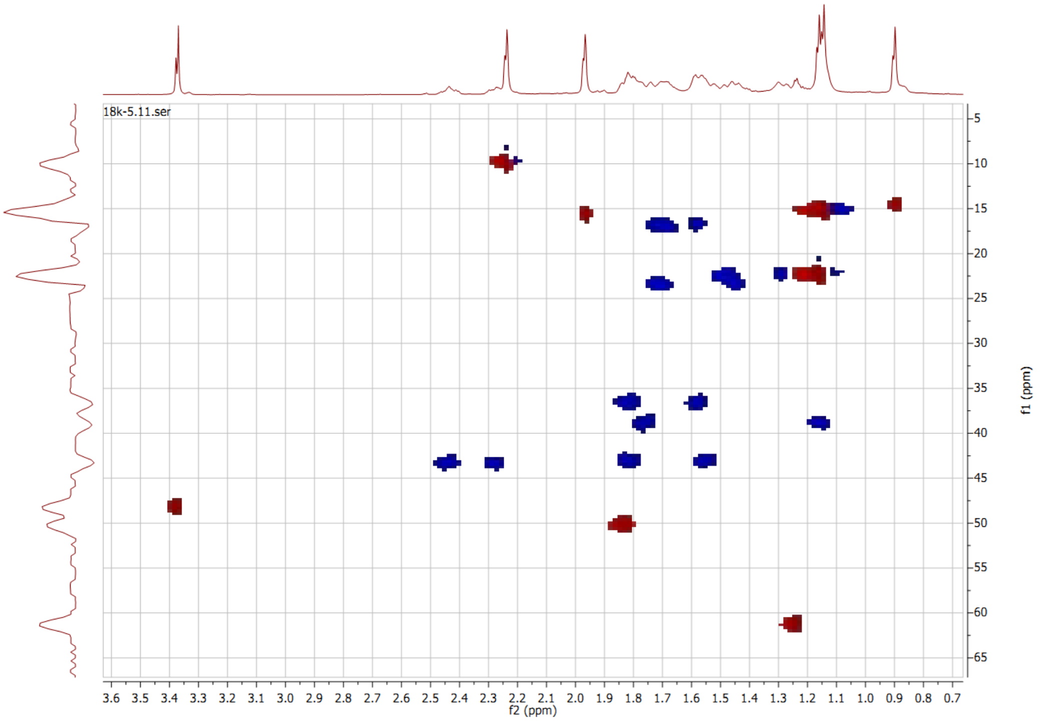

Figure S6. Expand of HSQC-DEPT spectrum of compound **1** (500 MHz, $CDCl_3$ + CD_3OD)

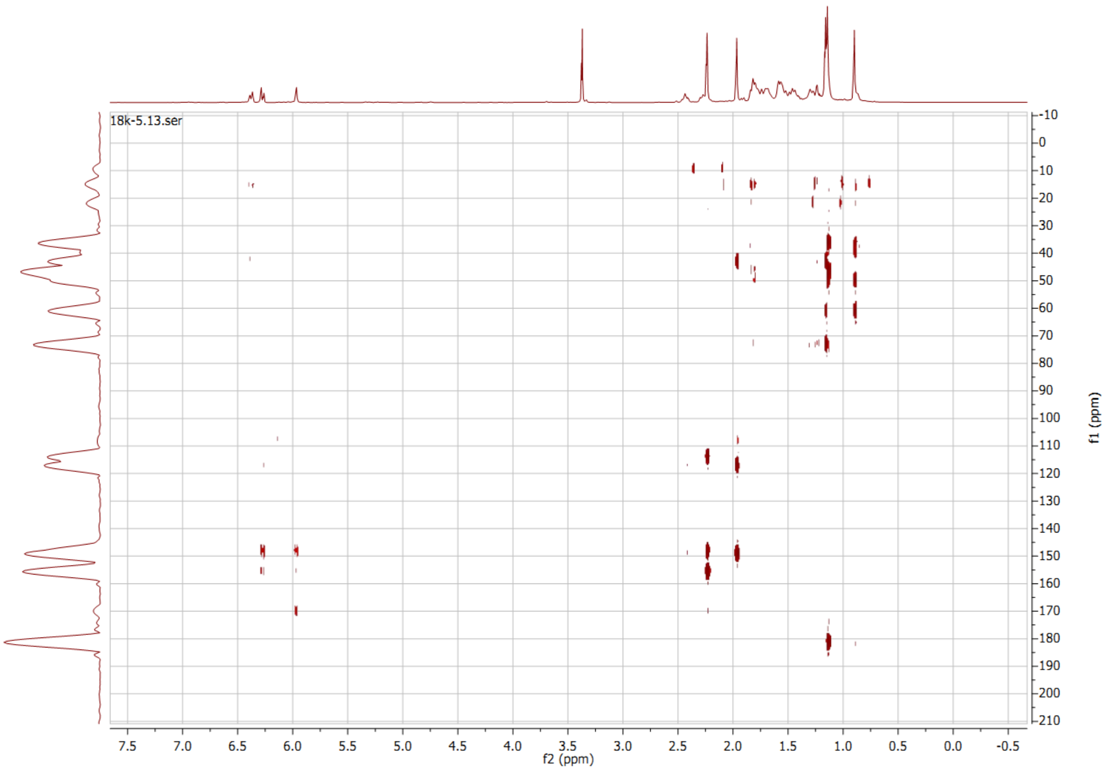

Figure S7. HMBC spectrum of compound **1** (500 MHz, $CDCl_3$ + CD_3OD)

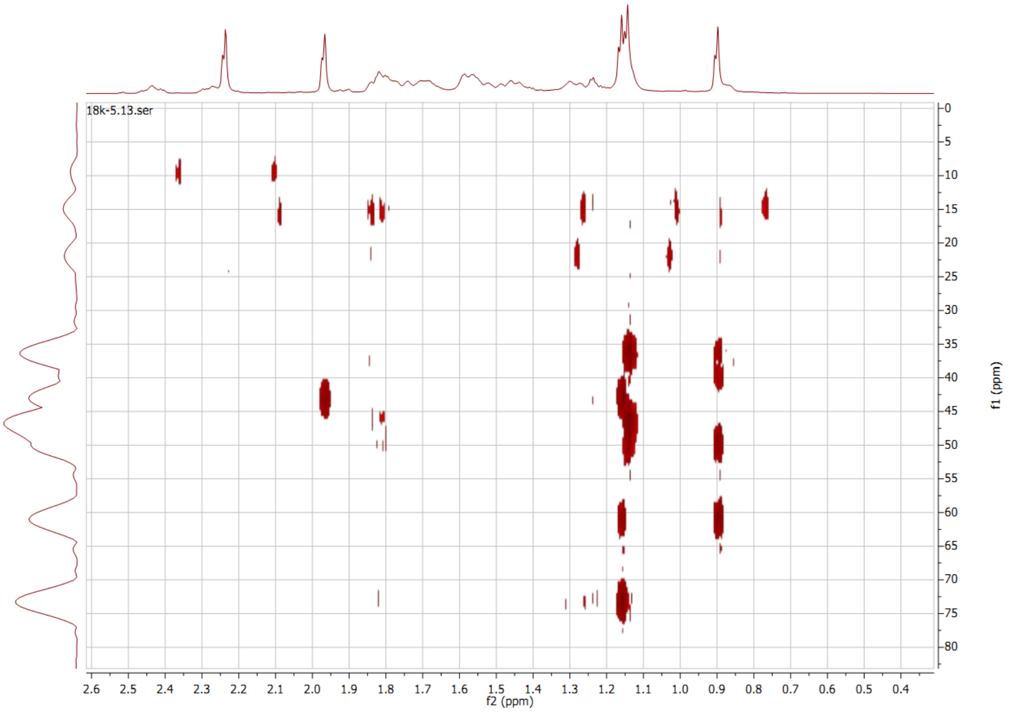

Figure S8. Expand of HMBC spectrum of compound **1** (500 MHz, $CDCl_3$ + CD_3OD)

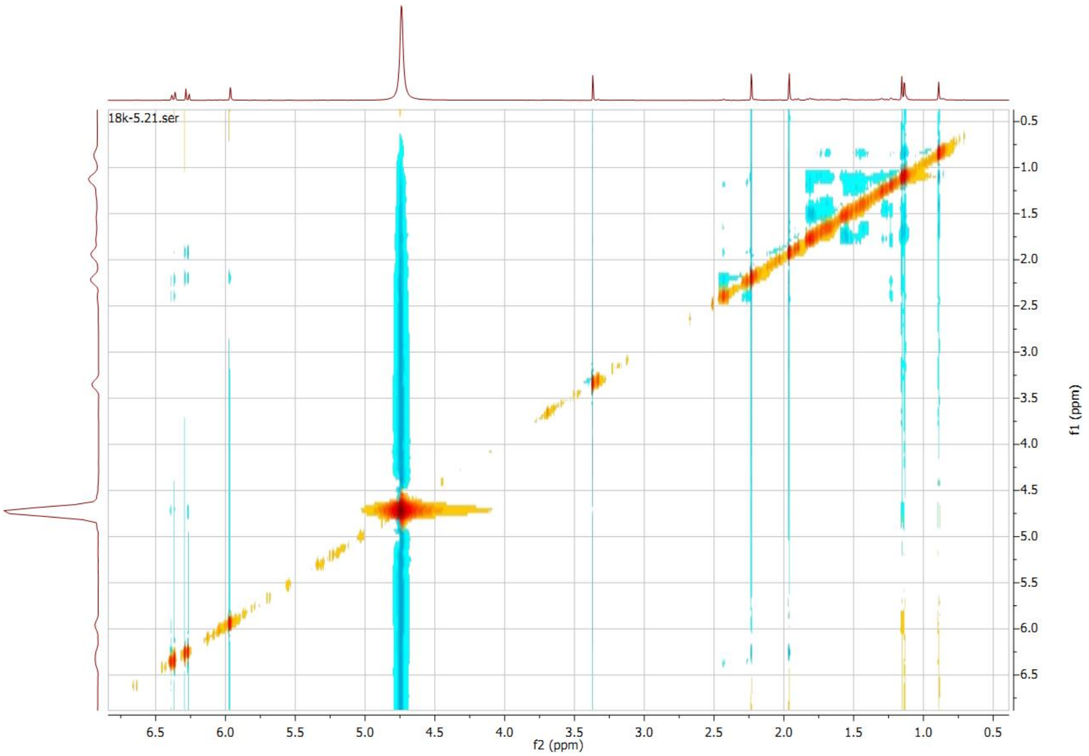

Figure S9. NOESY spectrum of compound **1** (500 MHz, $CDCl_3$ + CD_3OD)

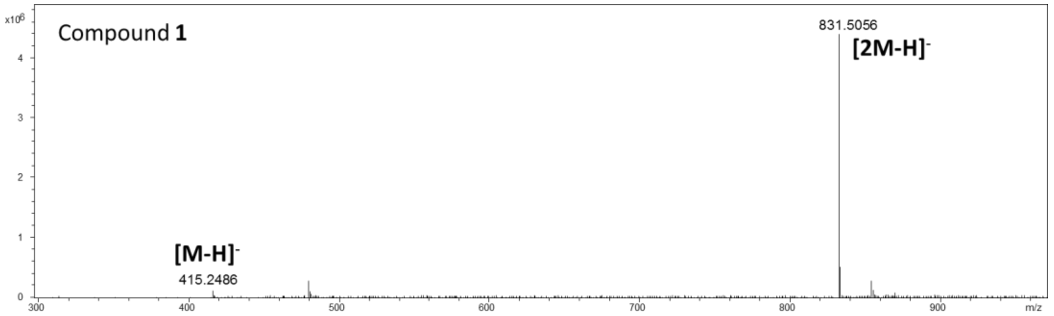

Figure S10. HRESIMS chromatogram of compound **1**

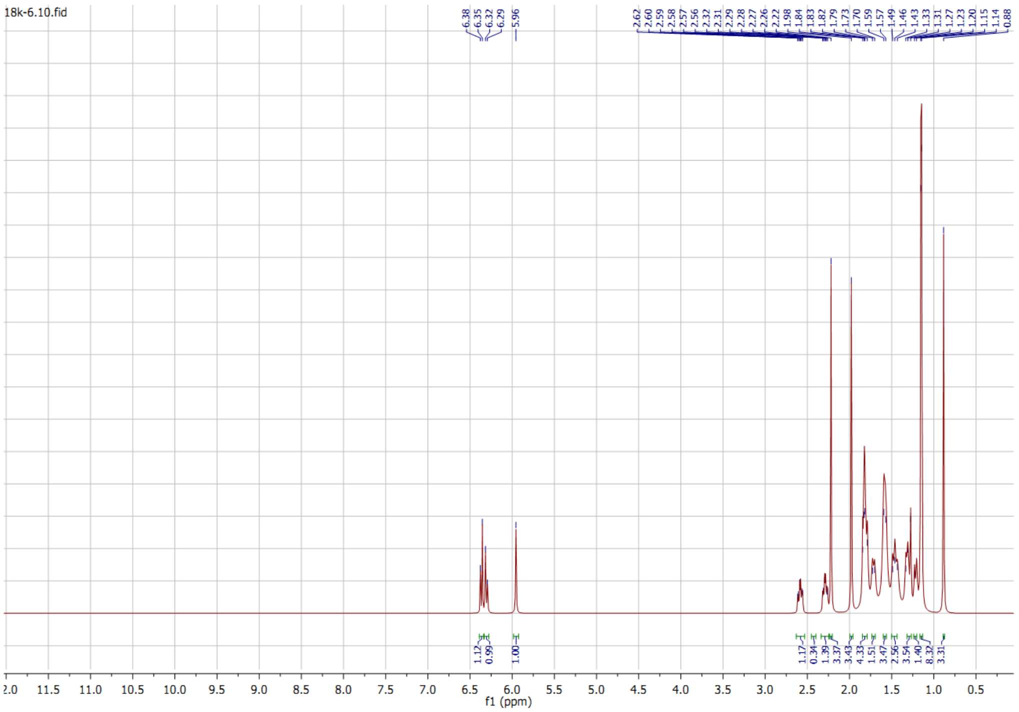

Figure S11. ^{1}H NMR spectrum of compound **2** (500 MHz, $CDCl_3$ + CD_3OD)

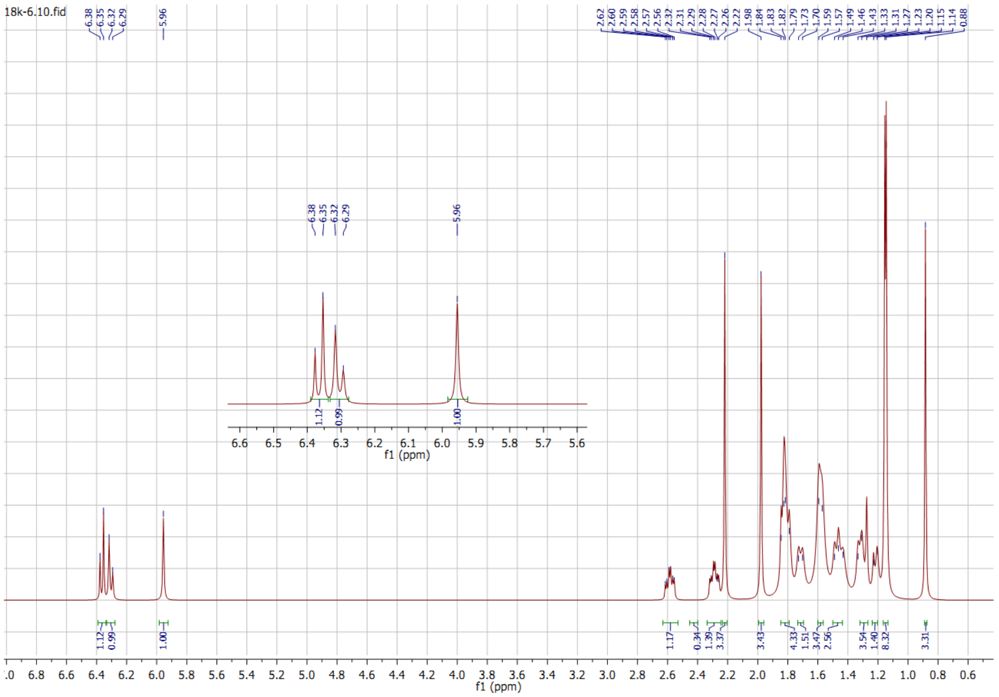

Figure S12. Expand of ^{1}H NMR spectrum of compound **2** (500 MHz, $CDCl_3$ + CD_3OD)

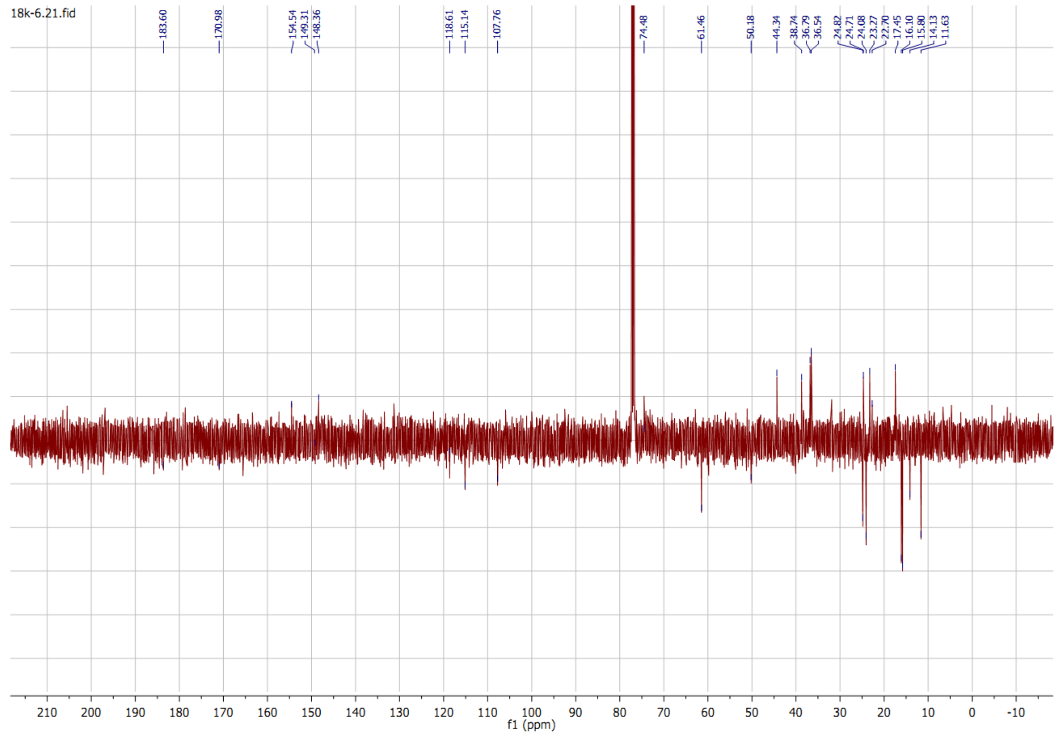

Figure S13. ^{13}C NMR (DEPTq) spectrum of compound **2** (500 MHz, $CDCl_3$)

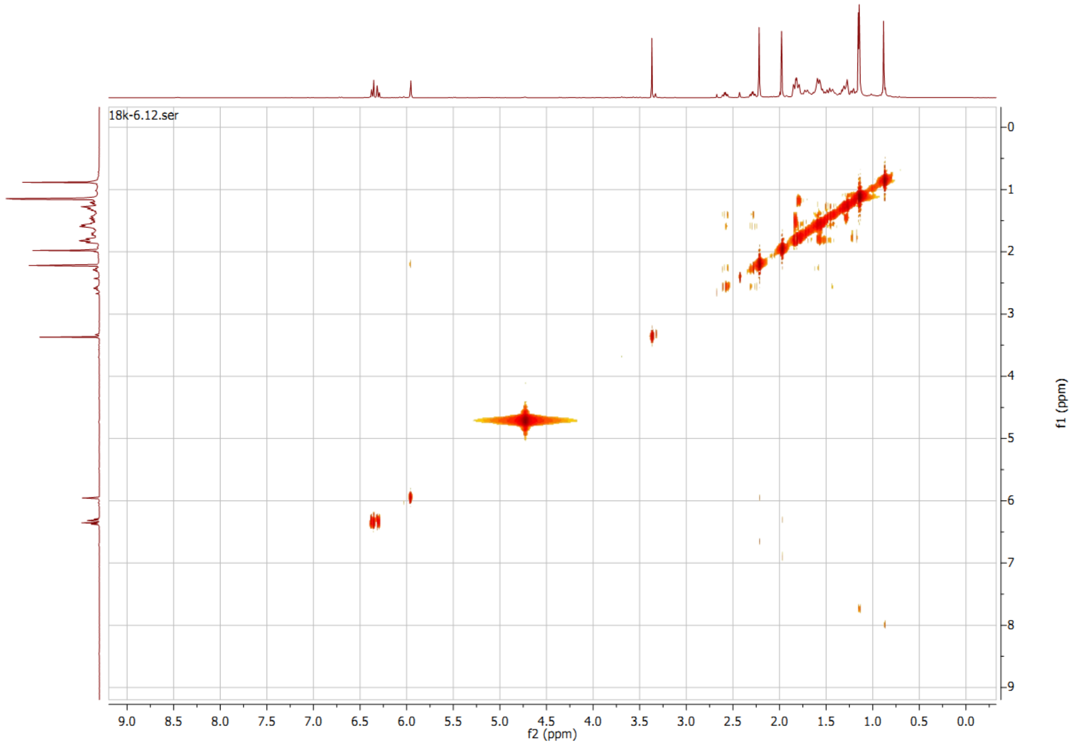

Figure S14. COSY spectrum of compound **2** (500 MHz, $CDCl_3$ + CD_3OD)

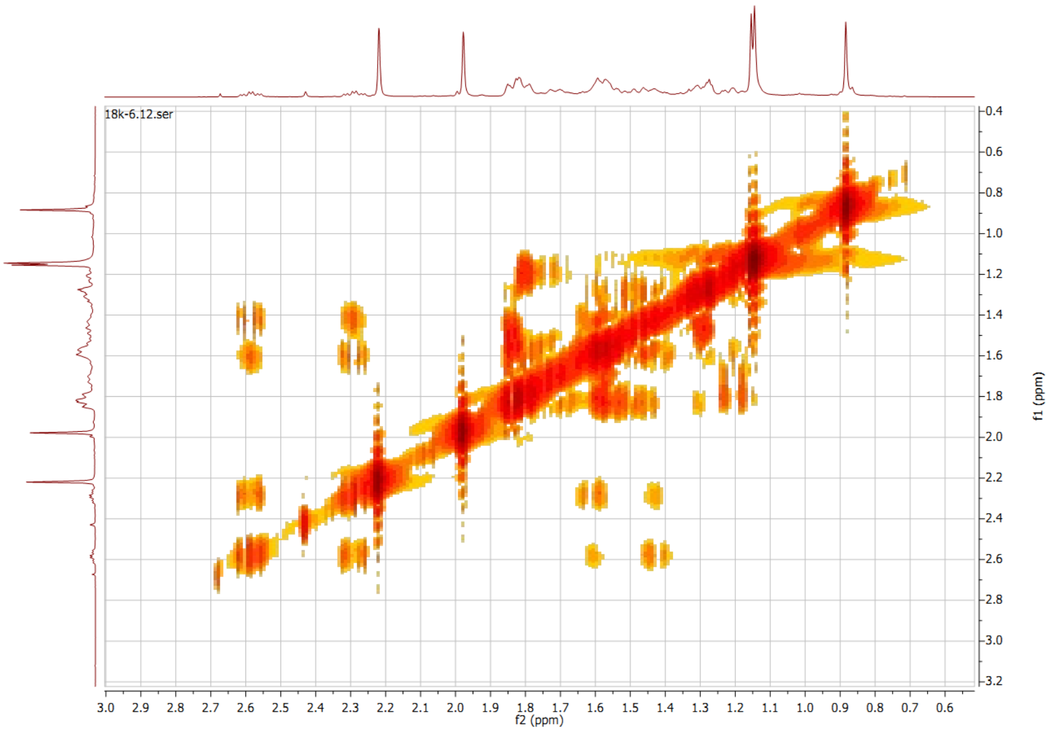

Figure S15. Expand of COSY spectrum of compound **2** (500 MHz, $CDCl_3$ + CD_3OD)

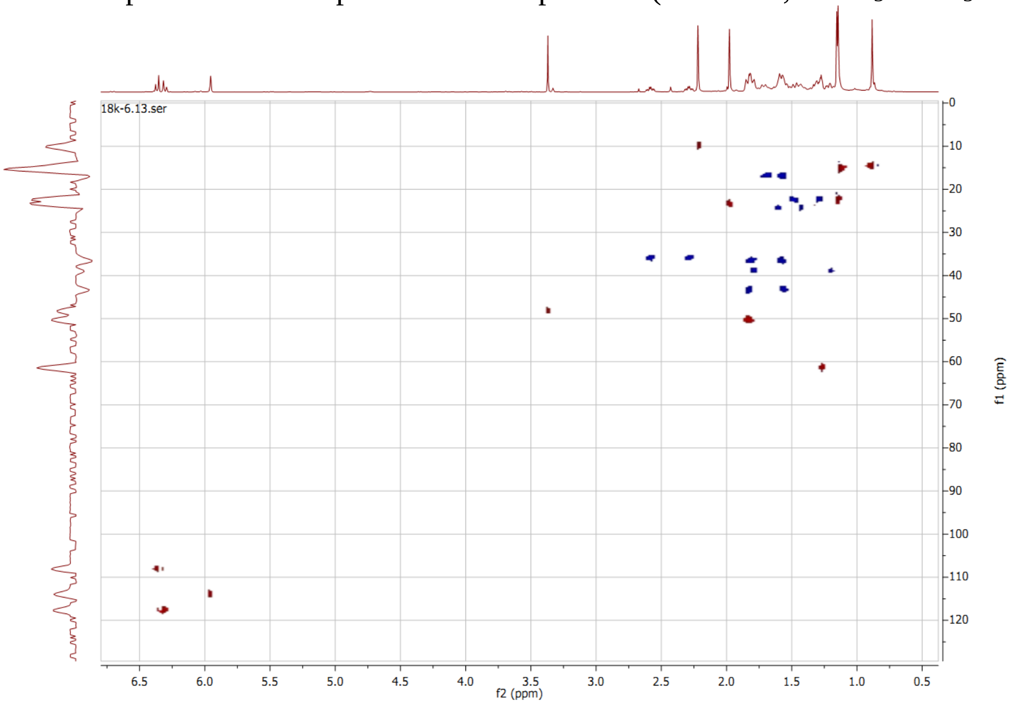

Figure S16. HSQC-DEPT spectrum of compound **2** (500 MHz, $CDCl_3$ + CD_3OD)

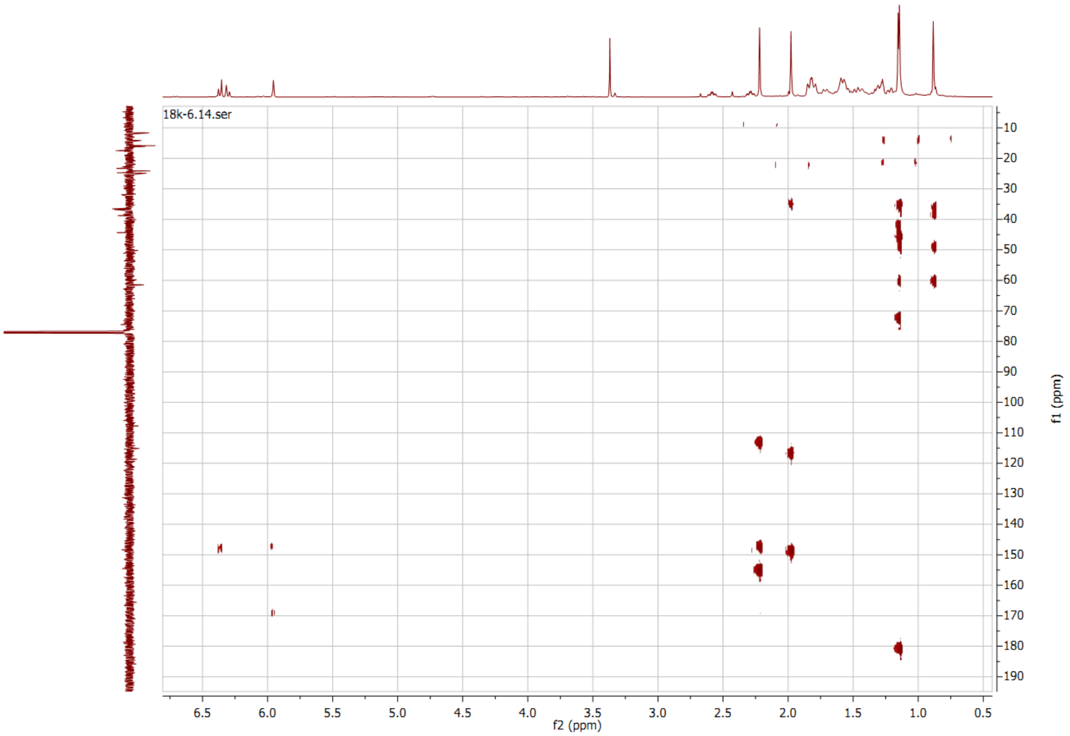

Figure S17. HMBC spectrum of compound **2** (500 MHz, $CDCl_3$ + CD_3OD)

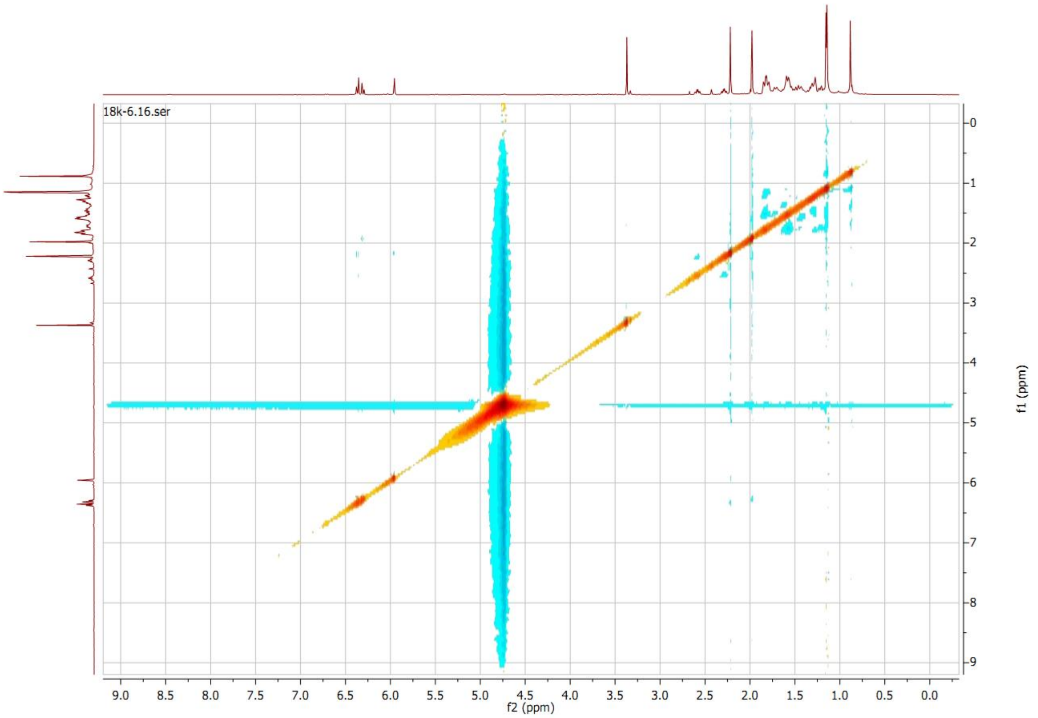

Figure S18. NOESY spectrum of compound **2** (500 MHz, $CDCl_3$ + CD_3OD)

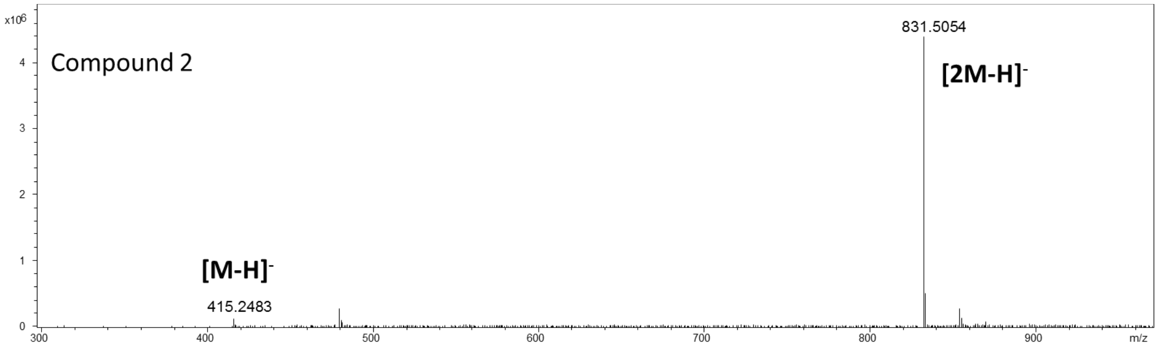

Figure S19. HRESIMS chromatogram of compound **2**

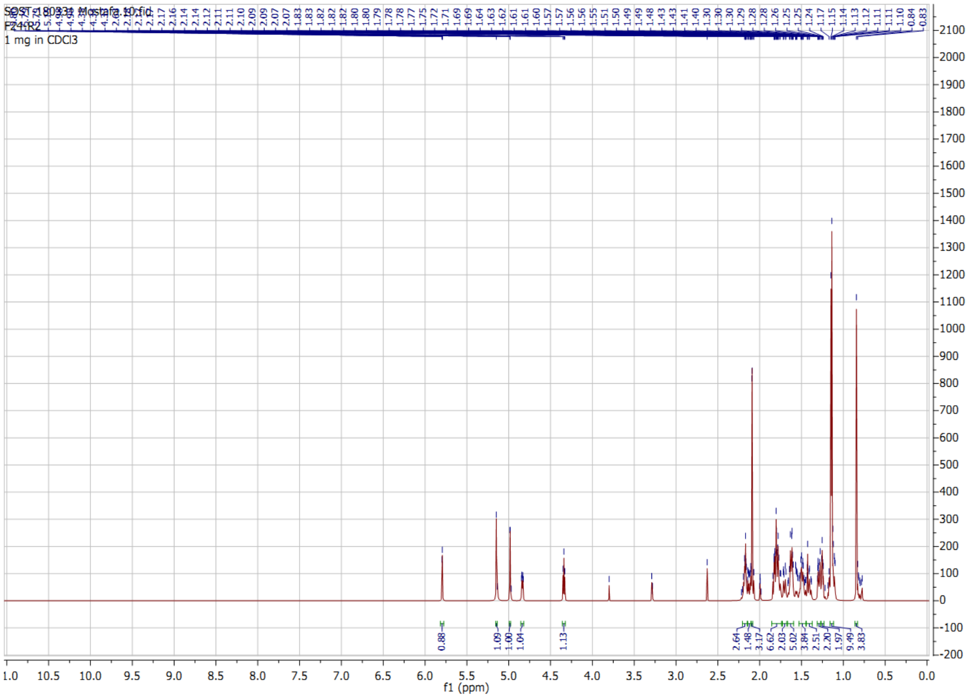

Figure S20. ^{1}H NMR spectrum of compound **3** (600 MHz, $CDCl_3$)

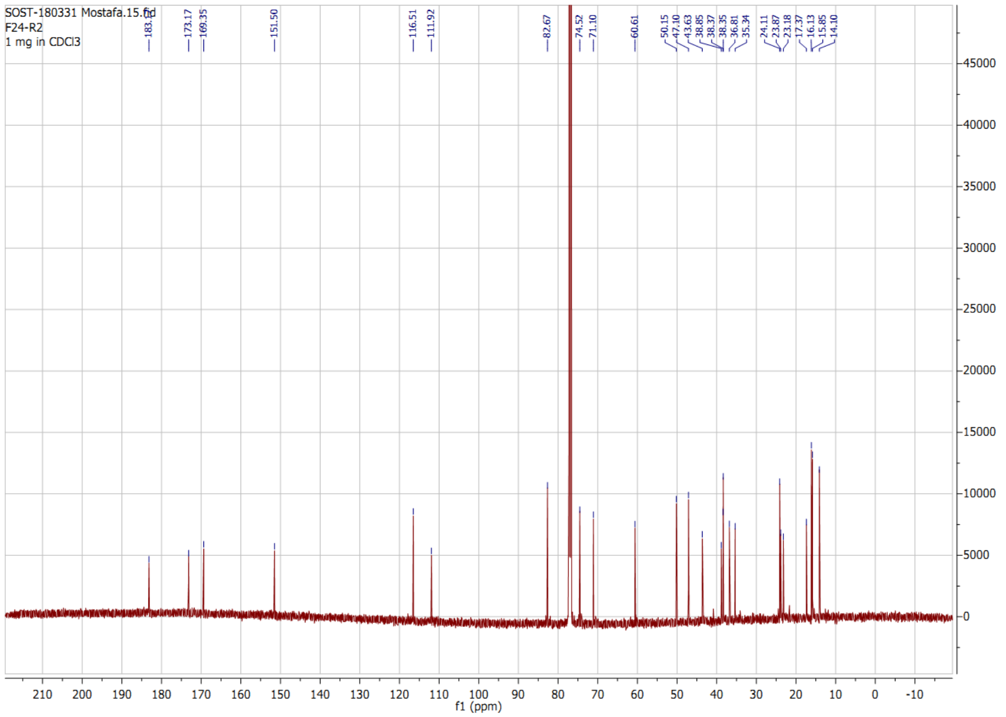

Figure S21. ^{13}C NMR spectrum of compound **3** (600 MHz, $CDCl_3$)

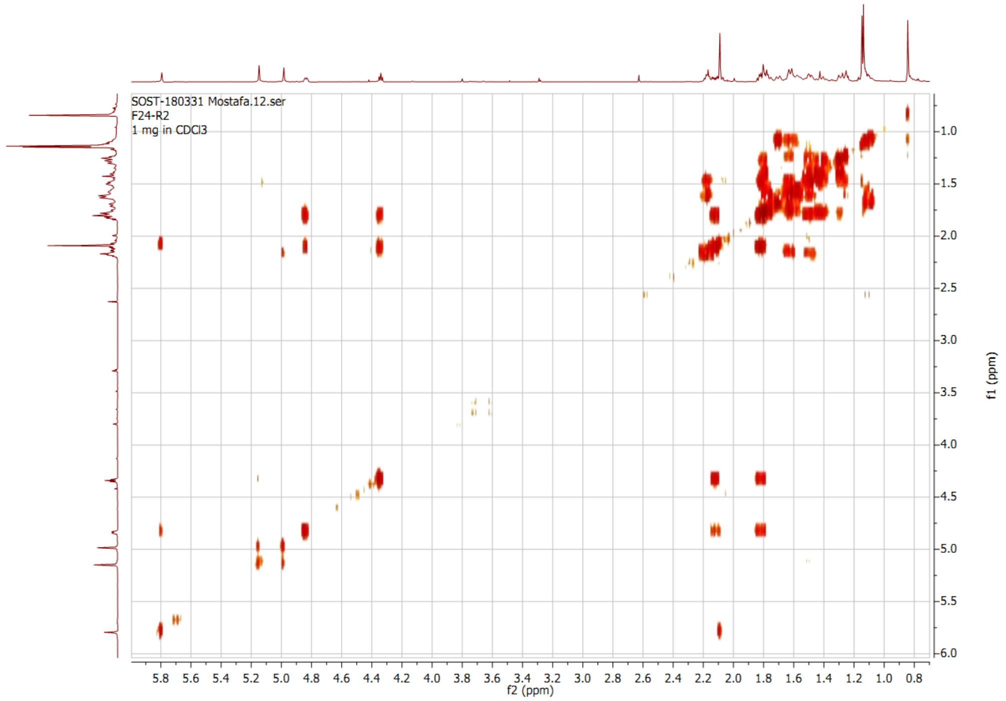

Figure S22. NOESY spectrum of compound **3** (600 MHz, $CDCl_3$)

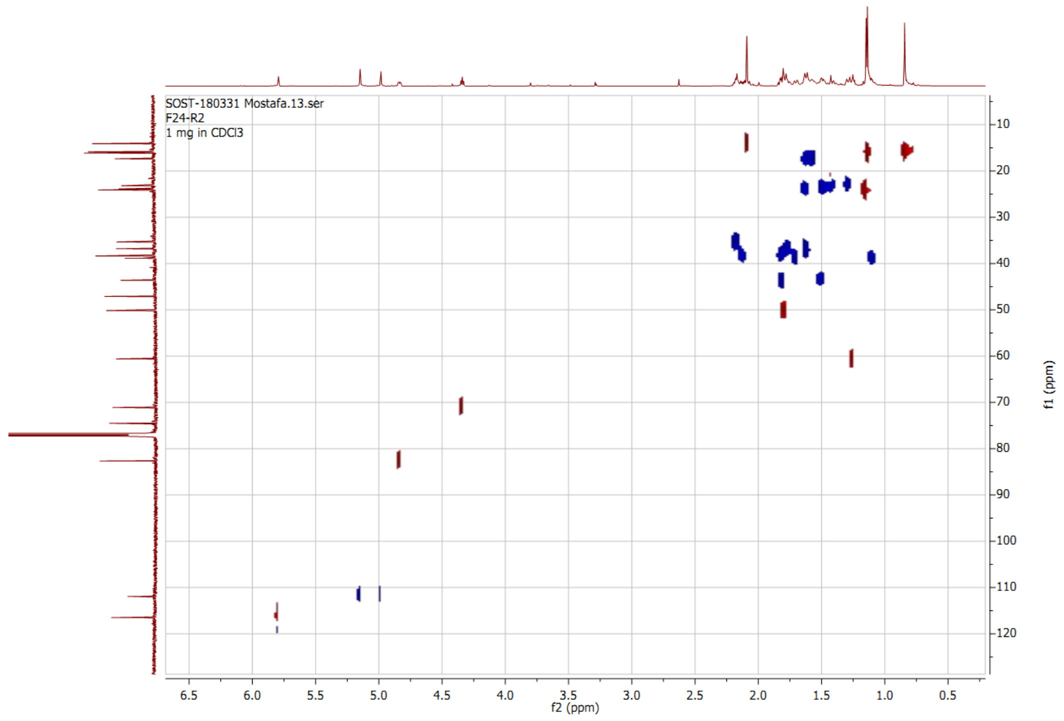

Figure S23. HSQC spectrum of compound **3** (600 MHz, $CDCl_3$)

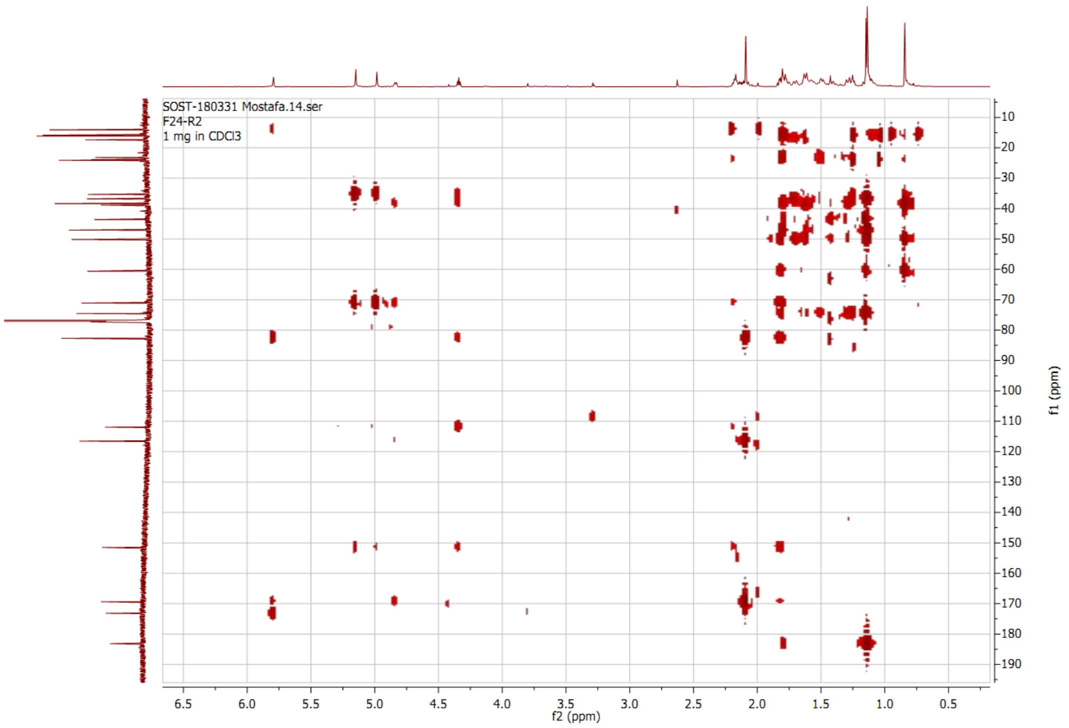

Figure S24. HMBC spectrum of compound **3** (600 MHz, $CDCl_3$)

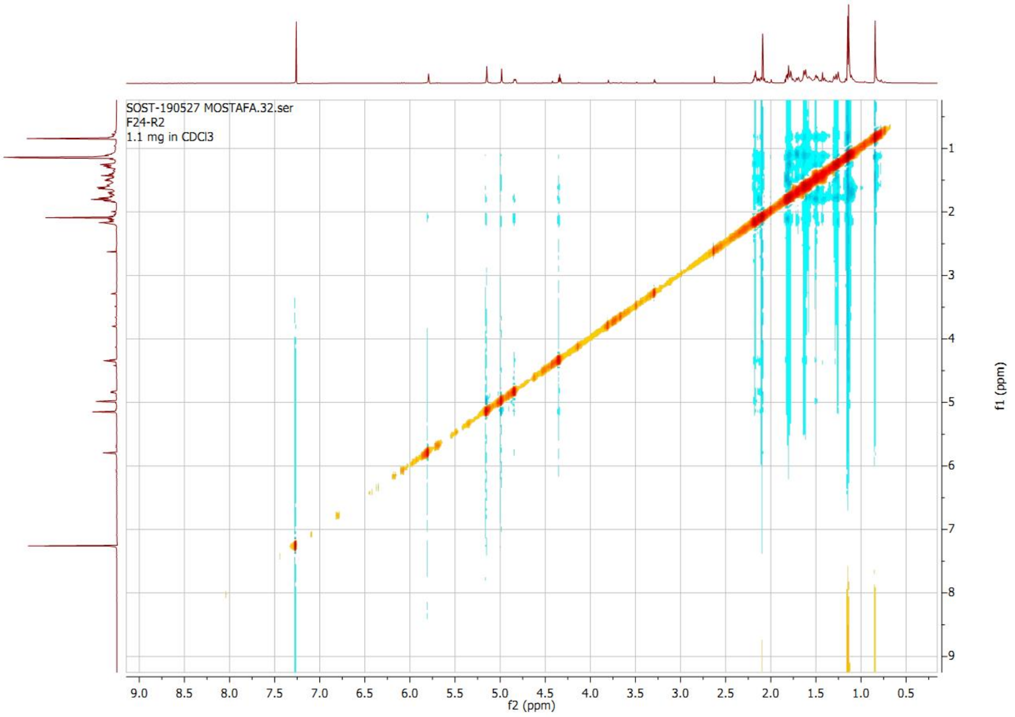

Figure S25. NOESY spectrum of compound **3** (600 MHz, $CDCl_3$)

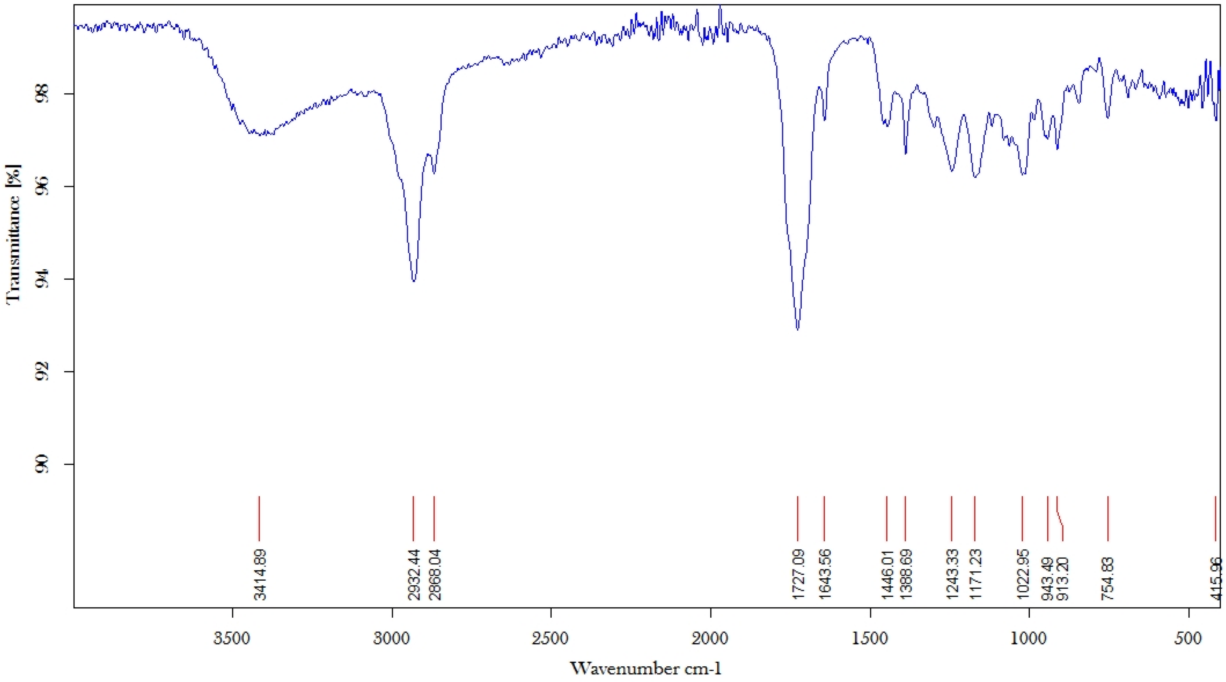

Figure S26. IR spectrum of compound **3**

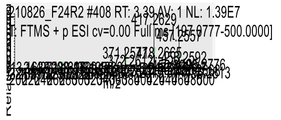

Figure S27. HRESIMS spectrum of compound **3**

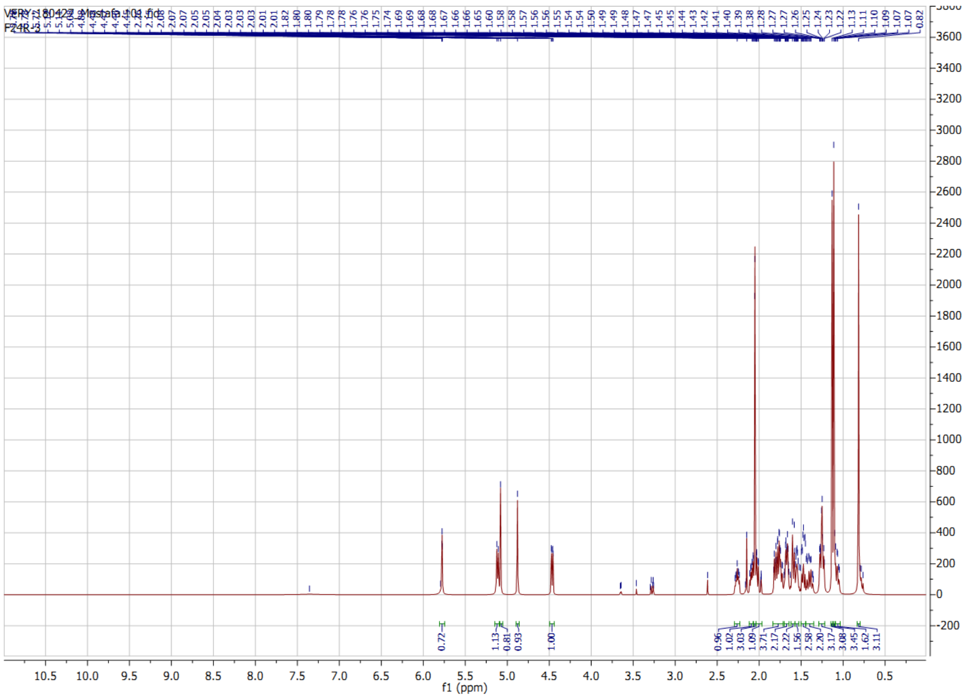

Figure S28. ^{1}H NMR spectrum of compound **4** (600 MHz, $CDCl_3$)

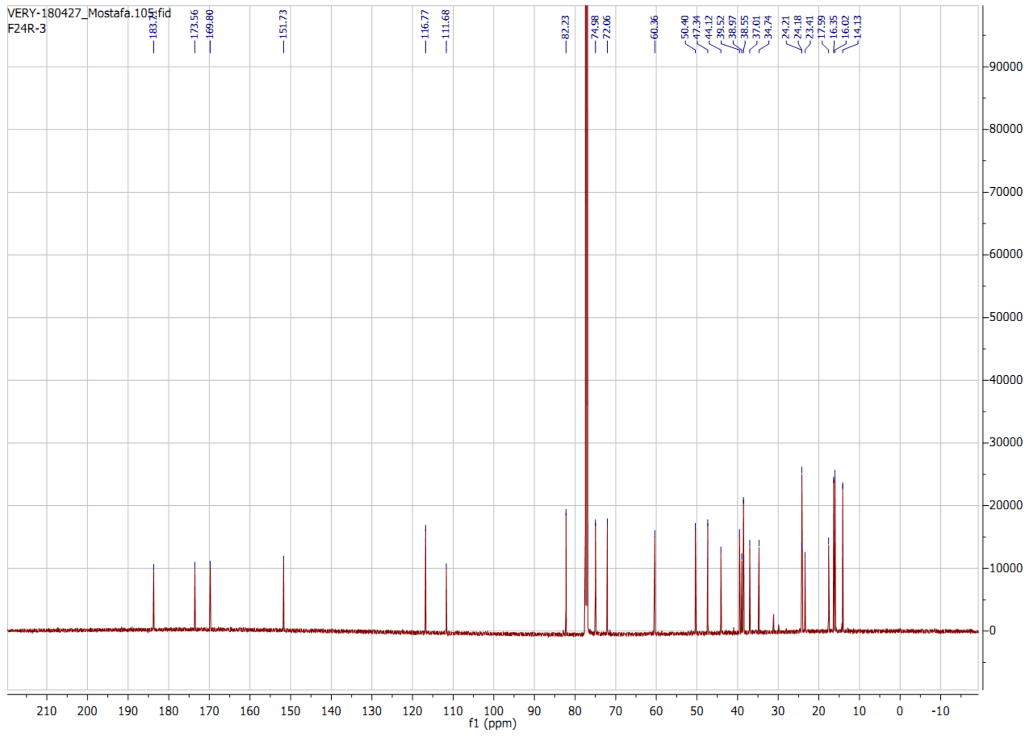

Figure S29. ^{13}C NMR spectrum of compound **4** (600 MHz, $CDCl_3$)

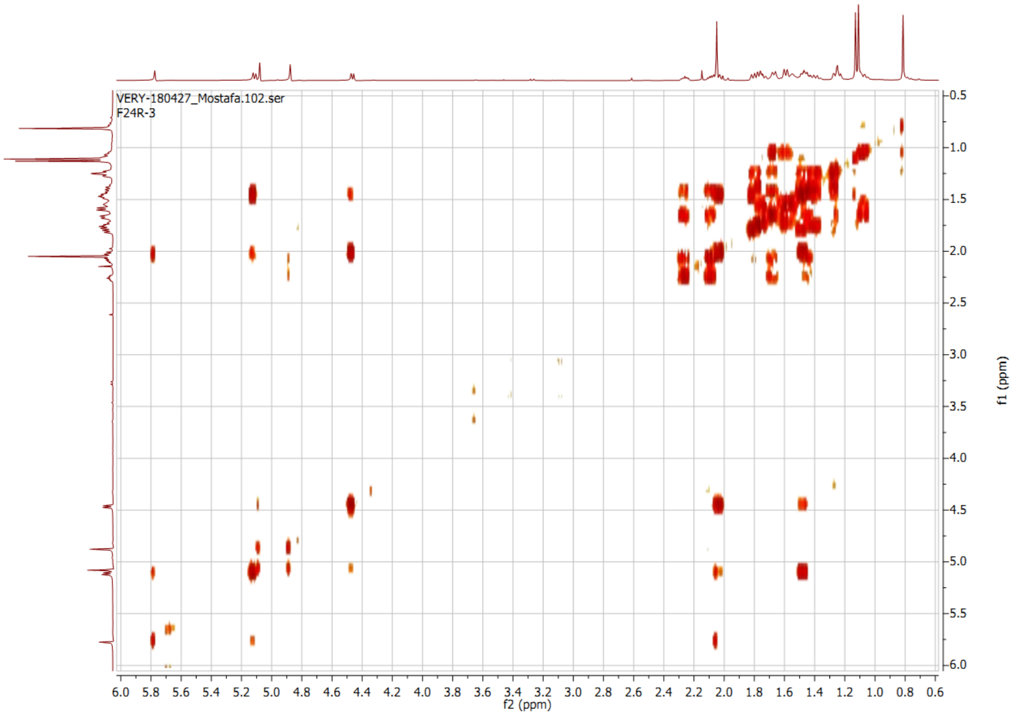

Figure S30. COSY spectrum of compound **4** (600 MHz, $CDCl_3$)

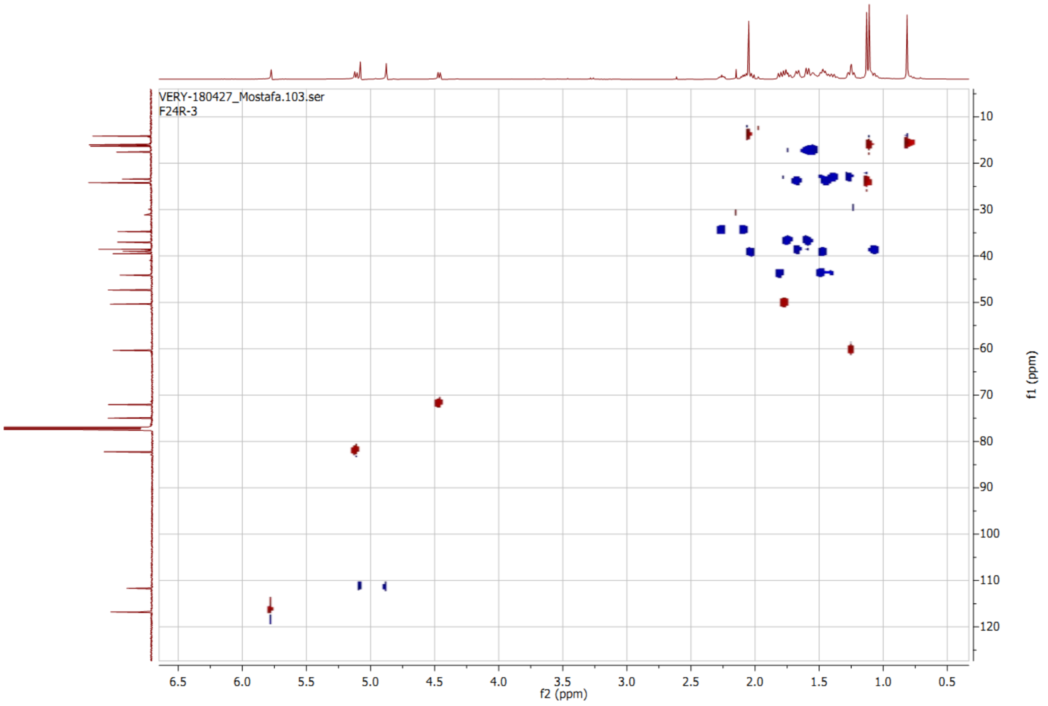

Figure S31. HSQC spectrum of compound **4** (600 MHz, $CDCl_3$)

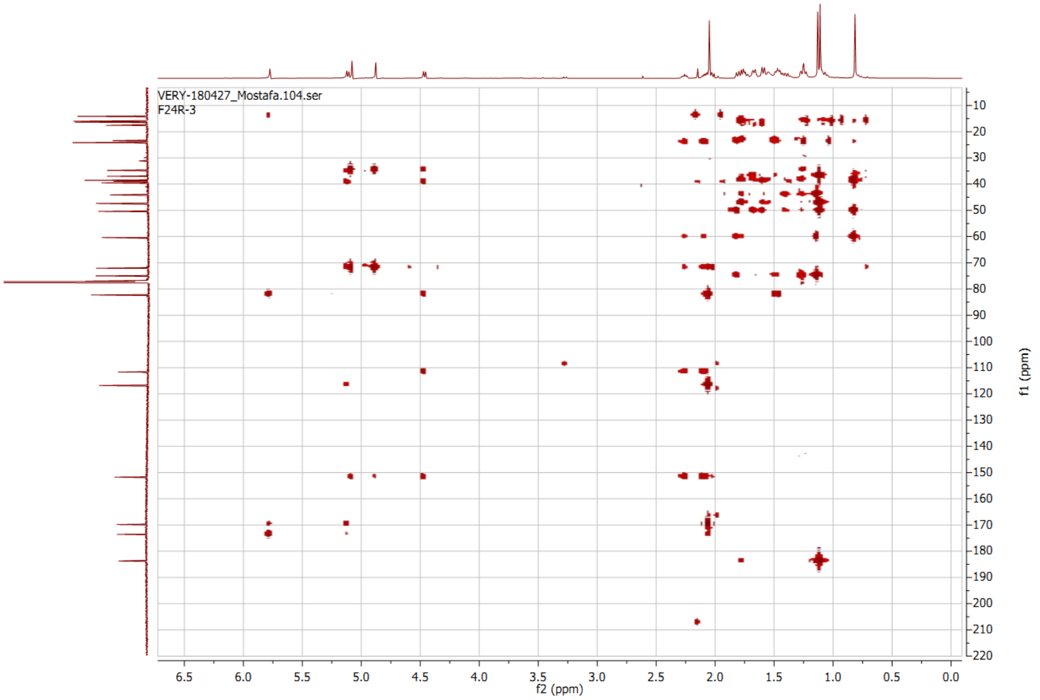

Figure S32. HMBC spectrum of compound **4** (600 MHz, $CDCl_3$)

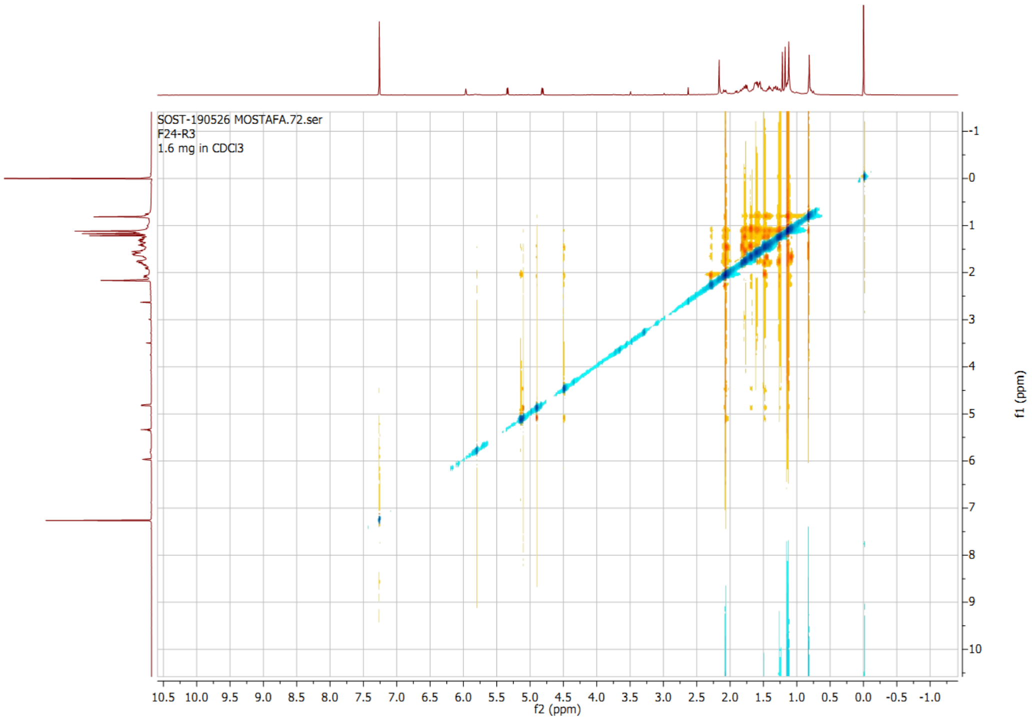

Figure S33. NOESY spectrum of compound **4** (600 MHz, $CDCl_3$)

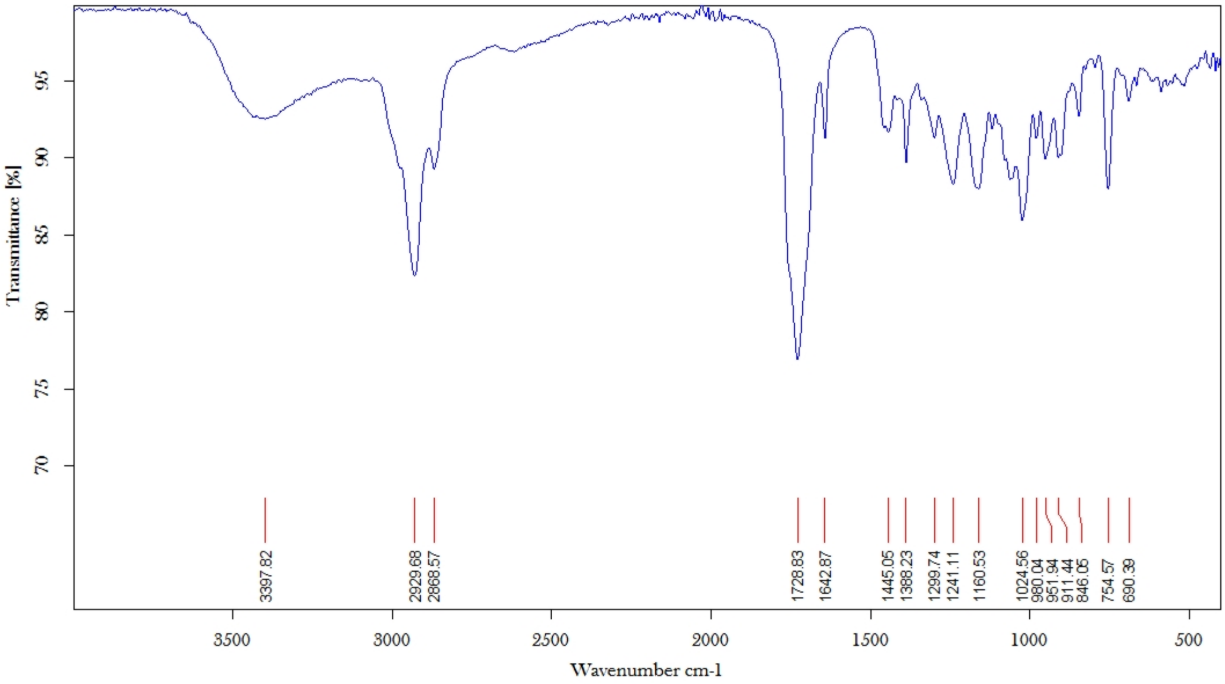

Figure S34. IR spectrum of compound **4**

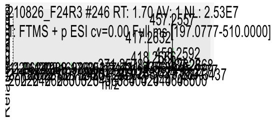

Figure S35. HRESIMS chromatogram of compound **4**

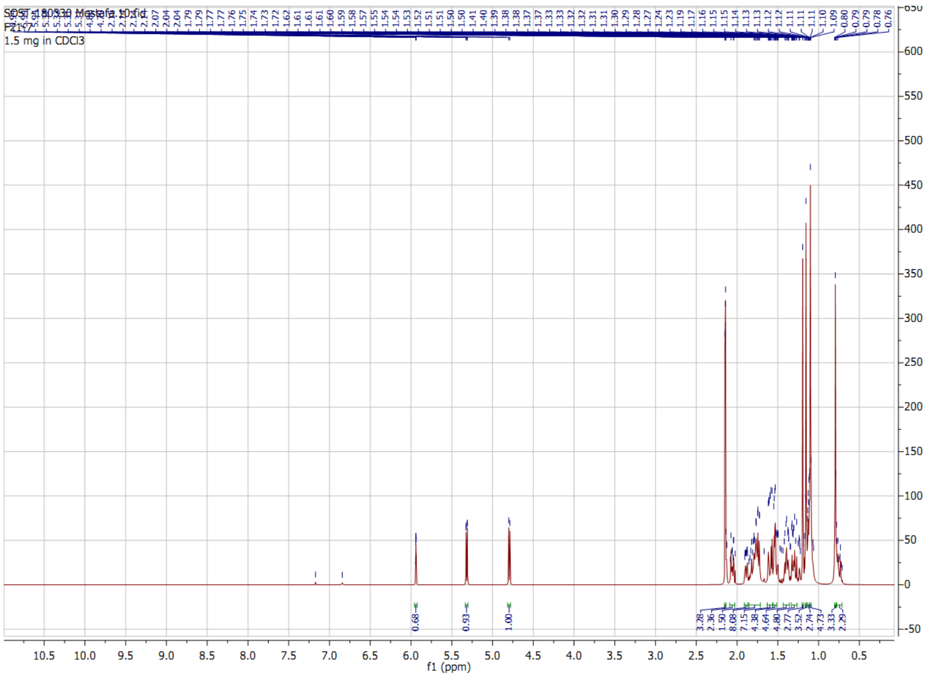

Figure S36. ^{1}H NMR spectrum of compound **5** (600 MHz, $CDCl_3$)

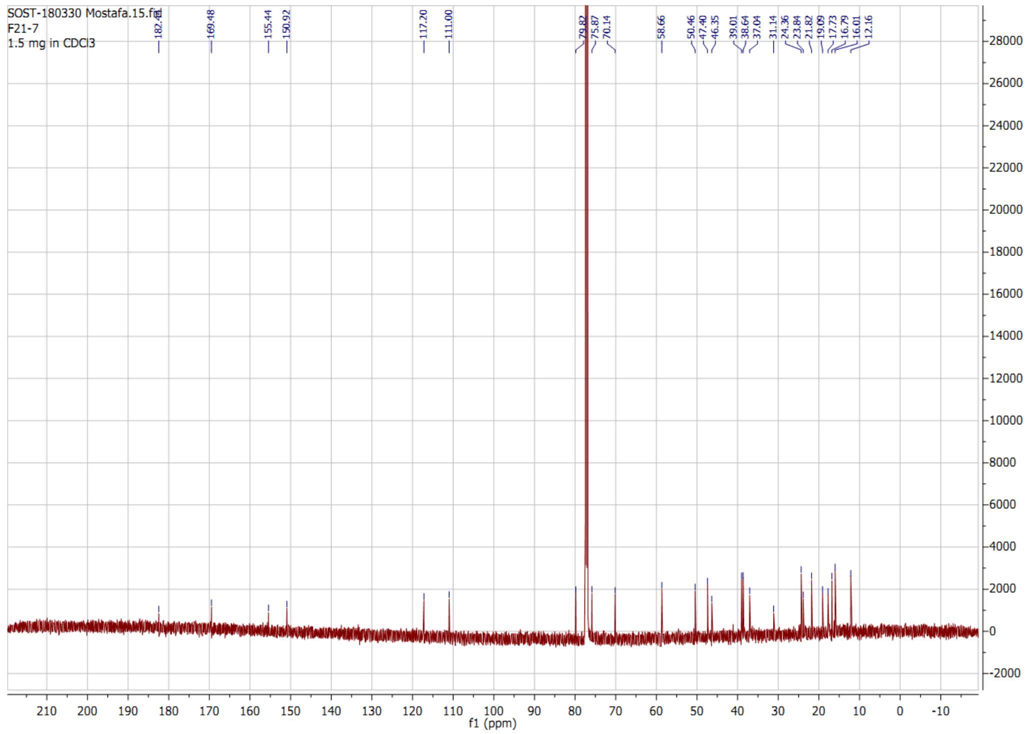

Figure S37. ^{13}C NMR spectrum of compound **5** (600 MHz, $CDCl_3$)

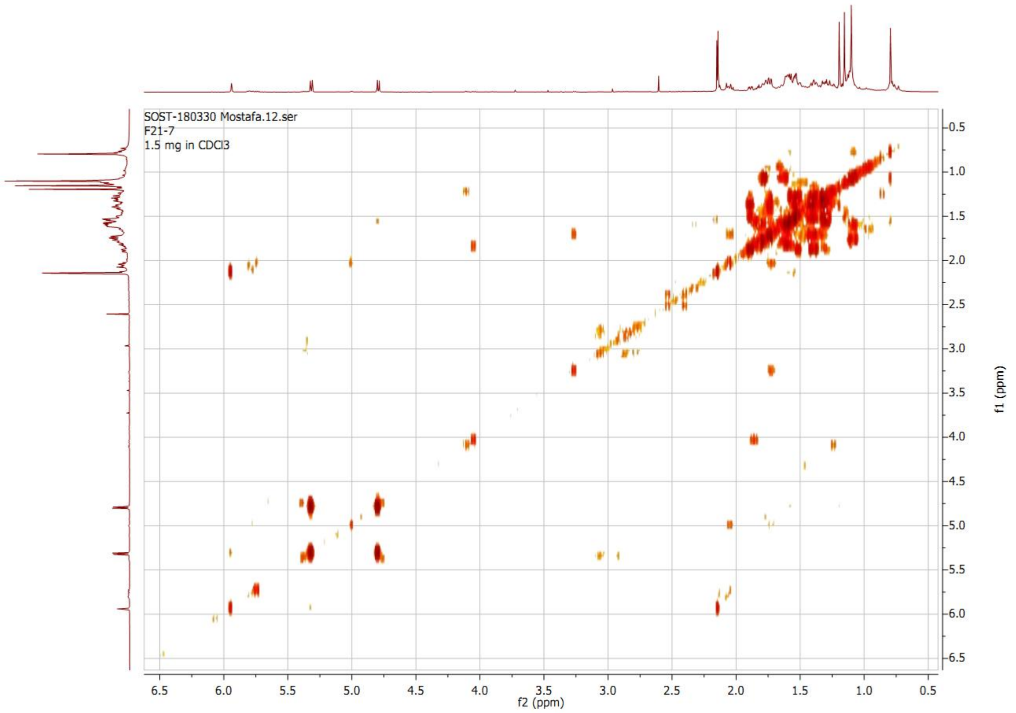

Figure S38. COSY spectrum of compound **5** (600 MHz, $CDCl_3$)

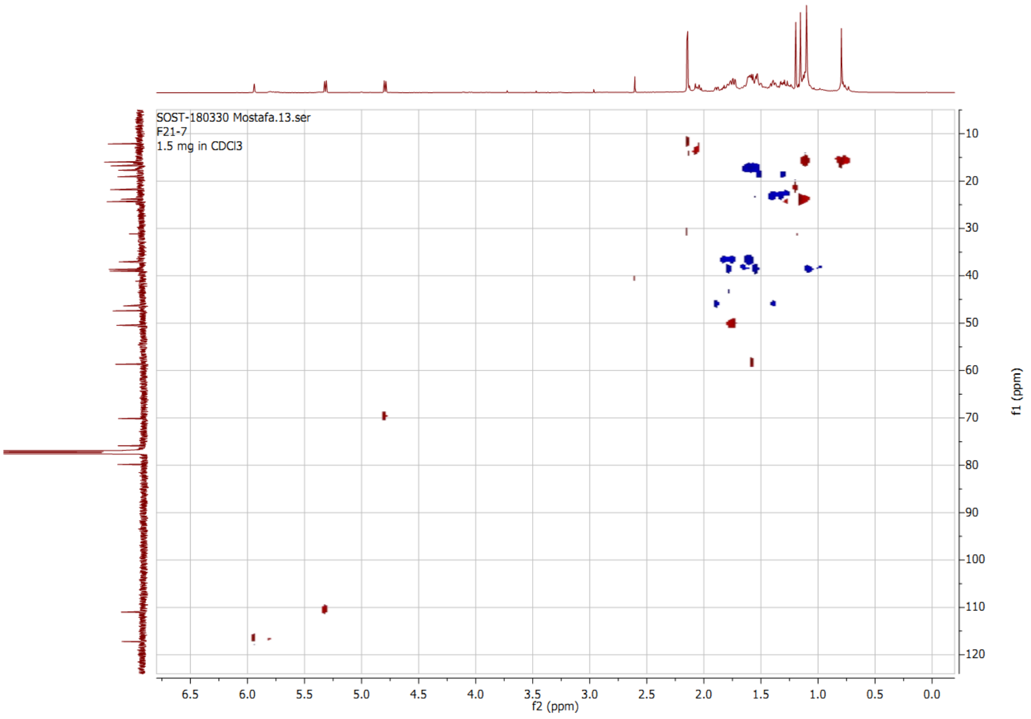

Figure S39. HSQC spectrum of compound **5** (600 MHz, $CDCl_3$)

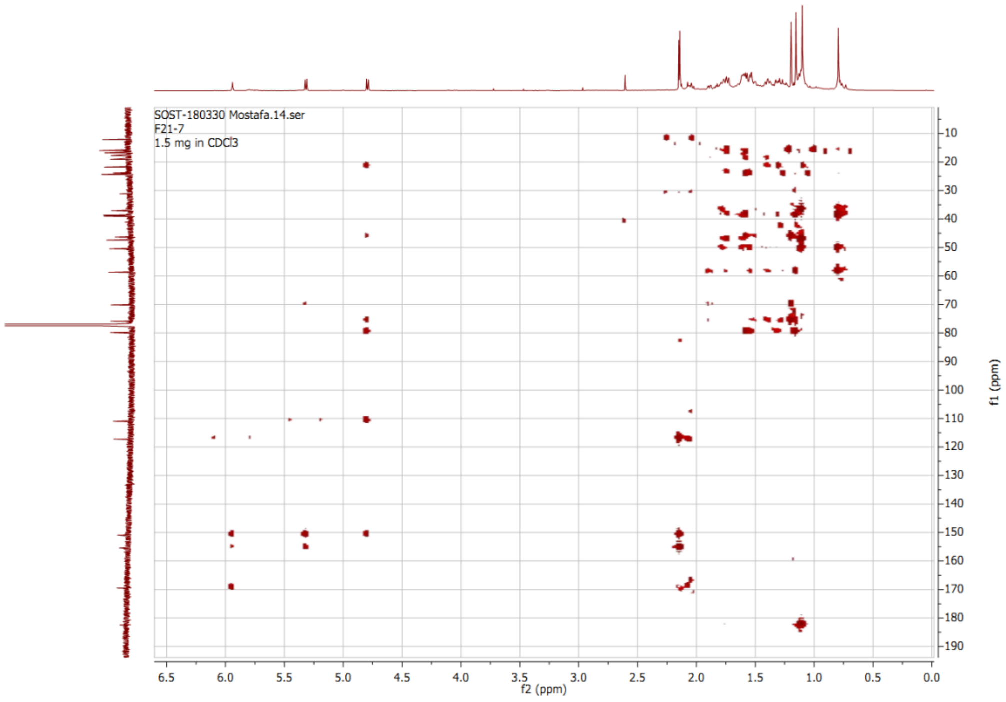

Figure S40. HMBC spectrum of compound **5** (600 MHz, $CDCl_3$)

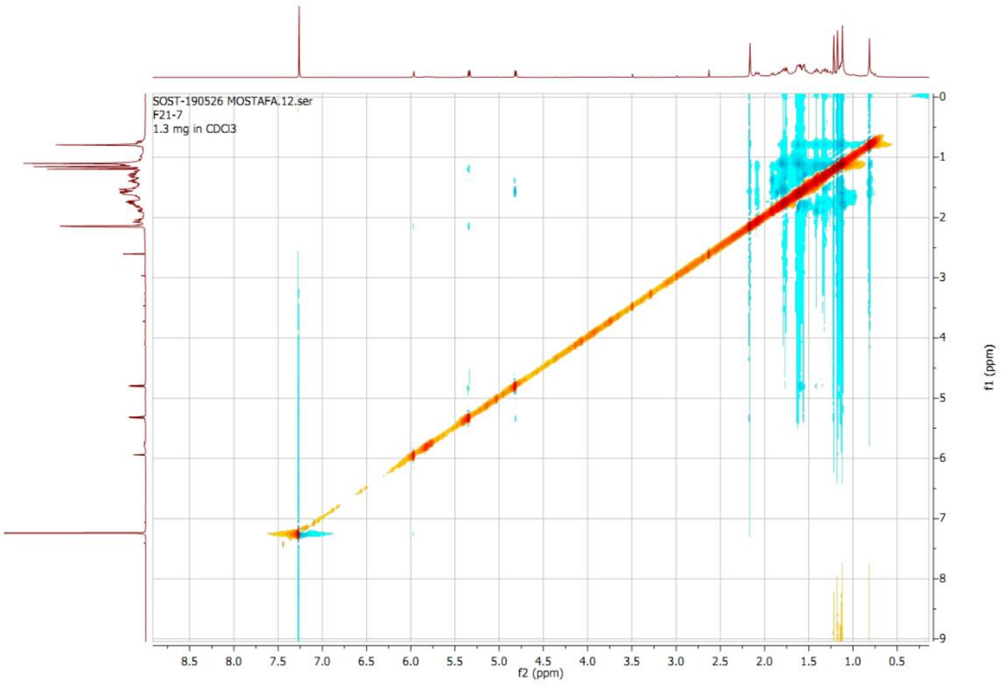

Figure S41. NOESY spectrum of compound **5** (600 MHz, $CDCl_3$)

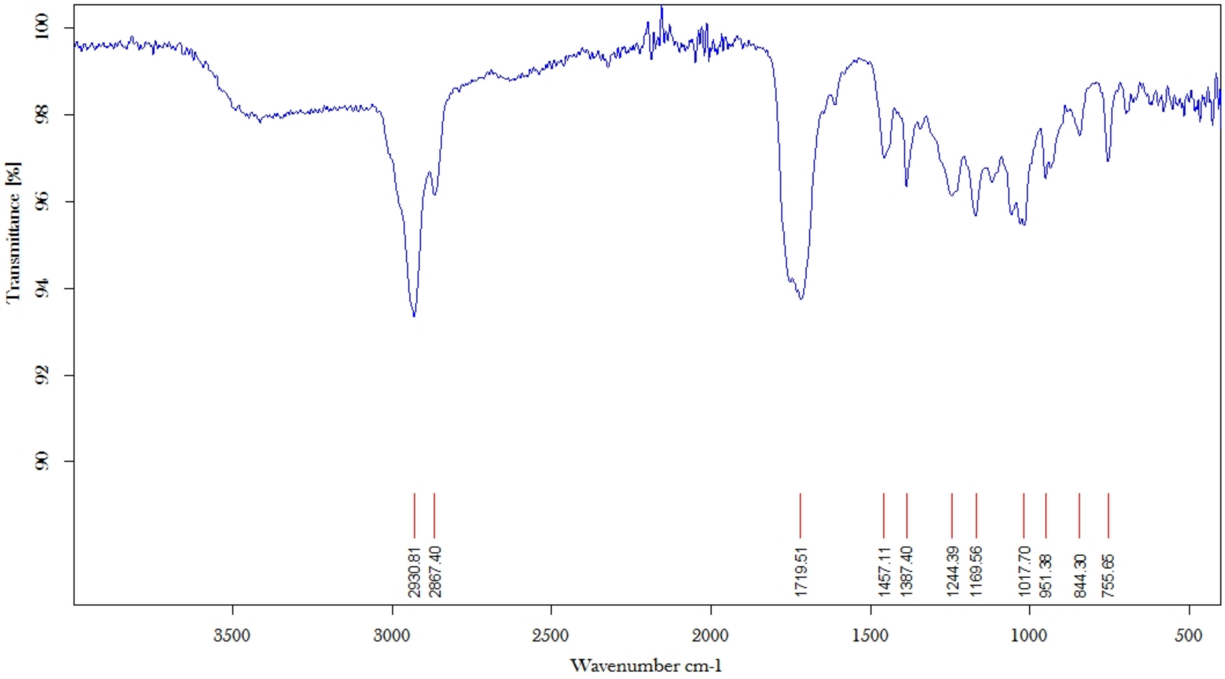

Figure S42. IR spectrum of compound **5**

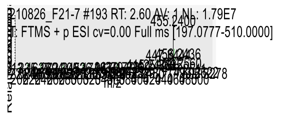

Figure S43. HRESIMS chromatogram of compound **5**

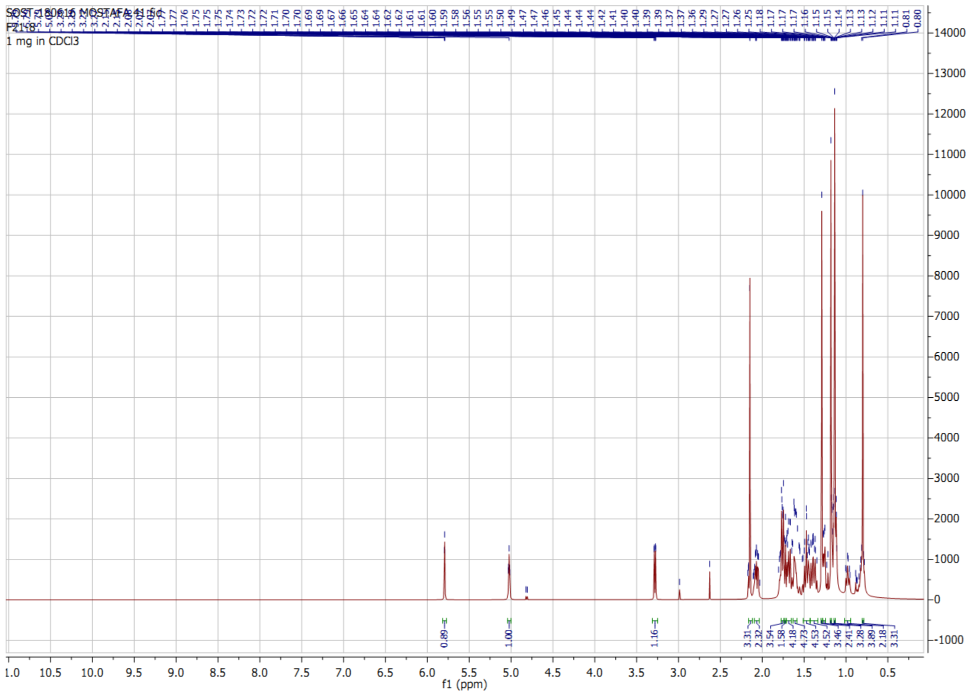

Figure S44. ^{1}H NMR spectrum of compound **6** (600 MHz, $CDCl_3$)

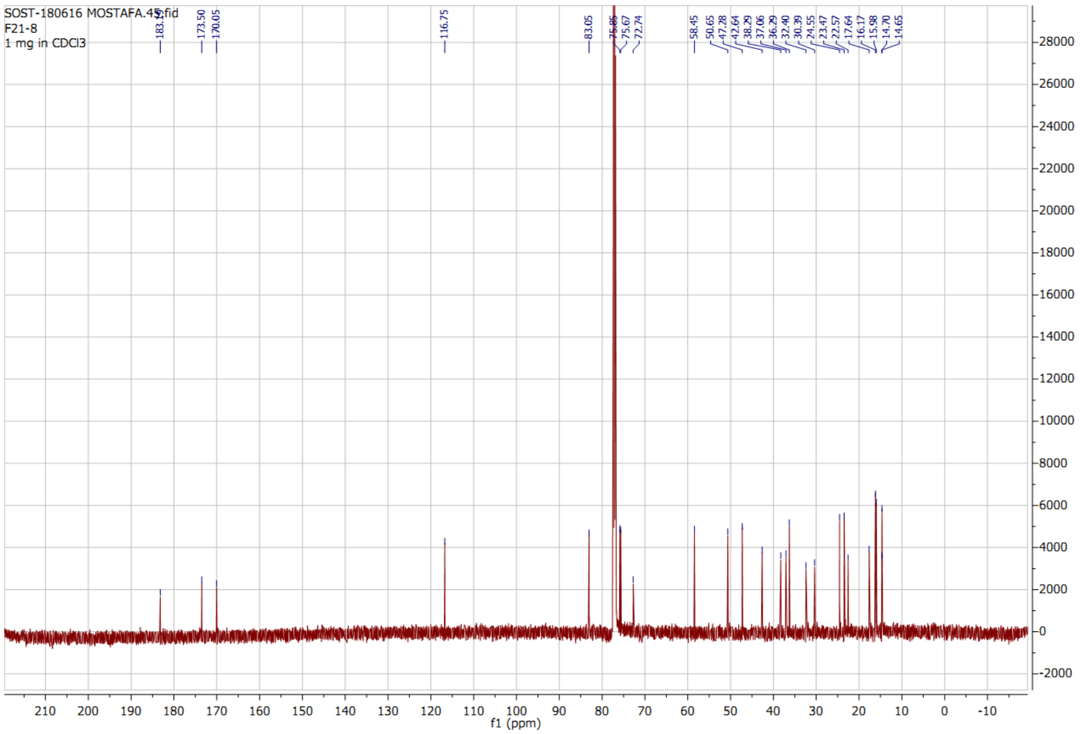

Figure S45. ^{13}C NMR spectrum of compound **6** (600 MHz, $CDCl_3$)

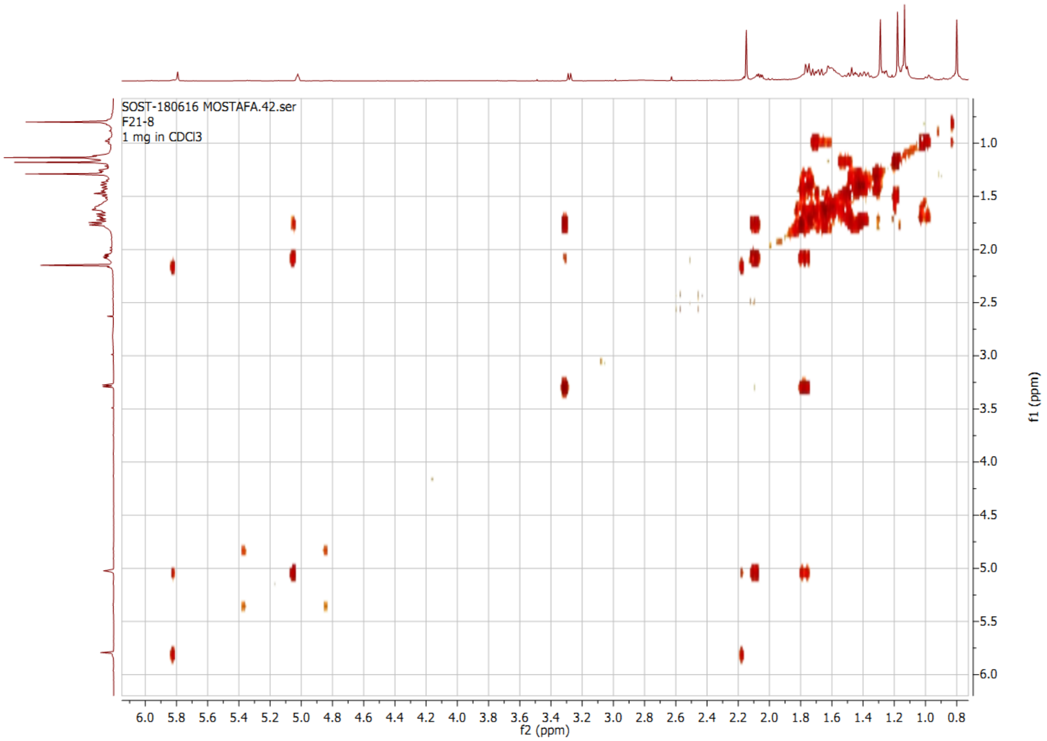

Figure S46. COSY spectrum of compound **6** (600 MHz, $CDCl_3$)

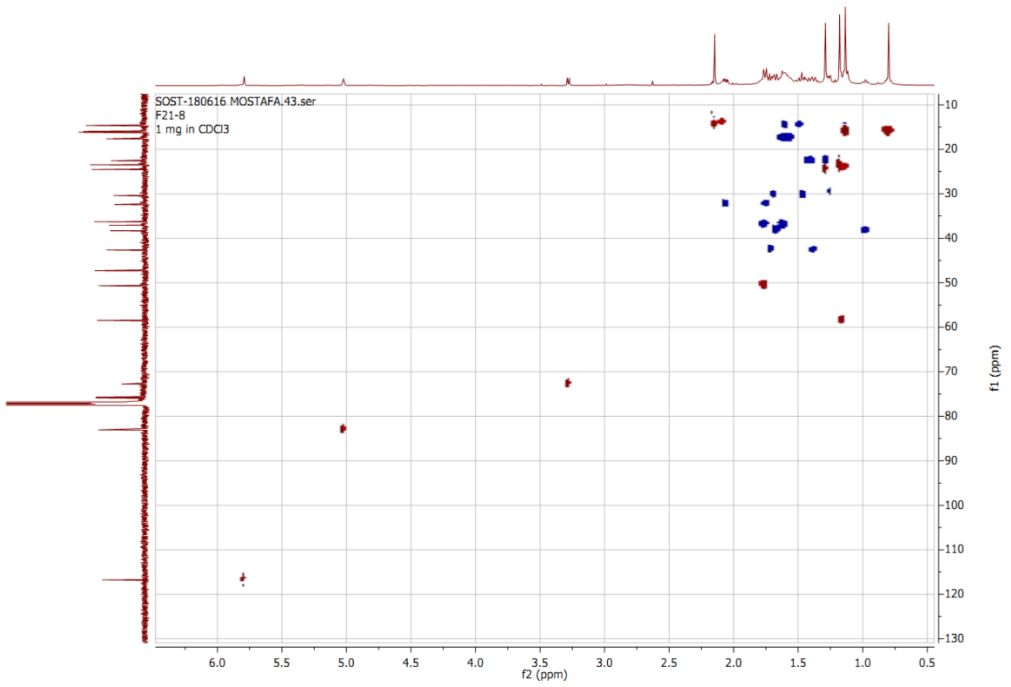

Figure S47. HSQC spectrum of compound **6** (600 MHz, $CDCl_3$)

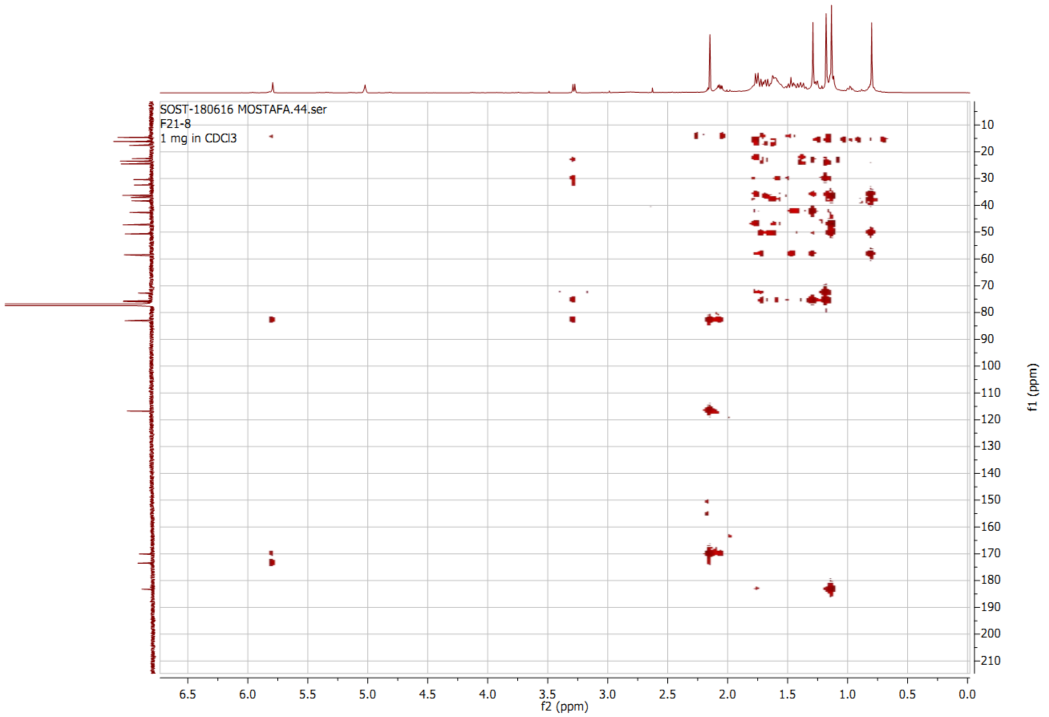

Figure S48. HMBC spectrum of compound **6** (600 MHz, $CDCl_3$)

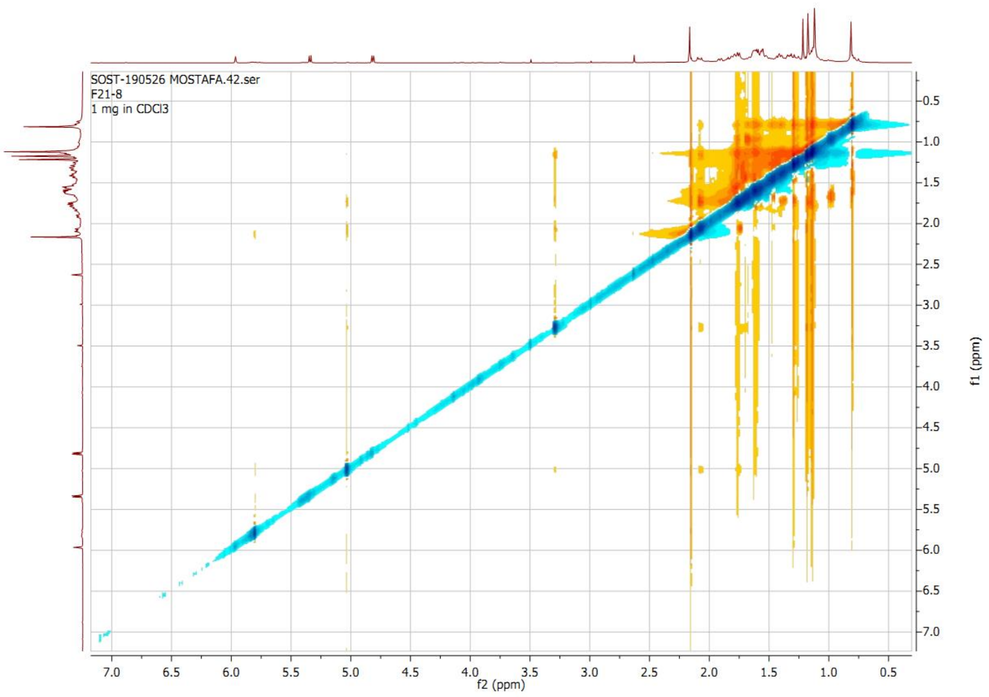

Figure S49. NOESY spectrum of compound **6** (600 MHz, $CDCl_3$)

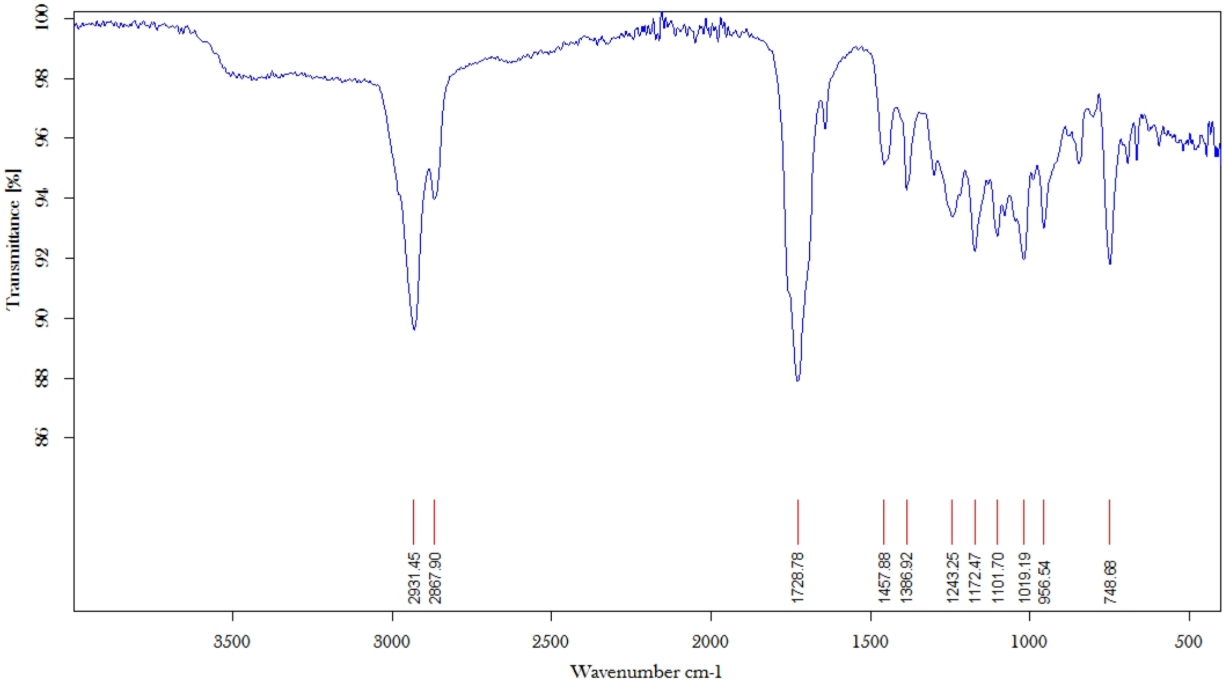

Figure S50. IR spectrum of compound **6**

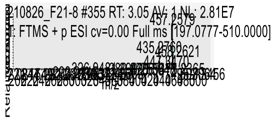

Figure S51. HRESIMS chromatogram of compound **6**

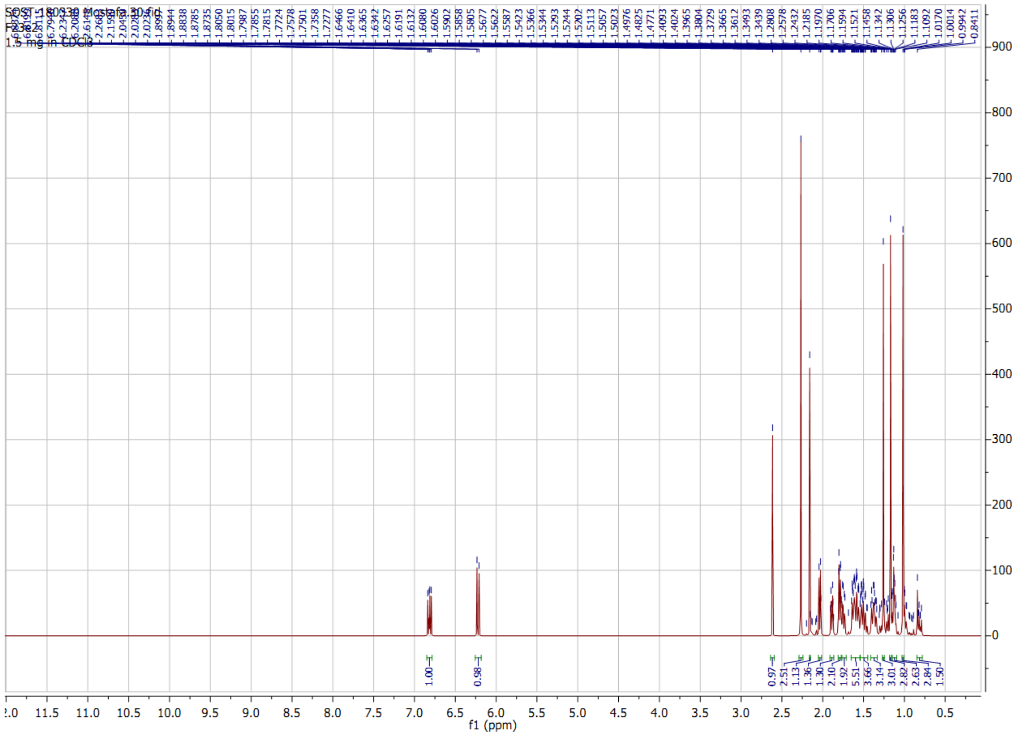

Figure S52. ^{1}H NMR spectrum of compound **7** (600 MHz, $CDCl_3$)

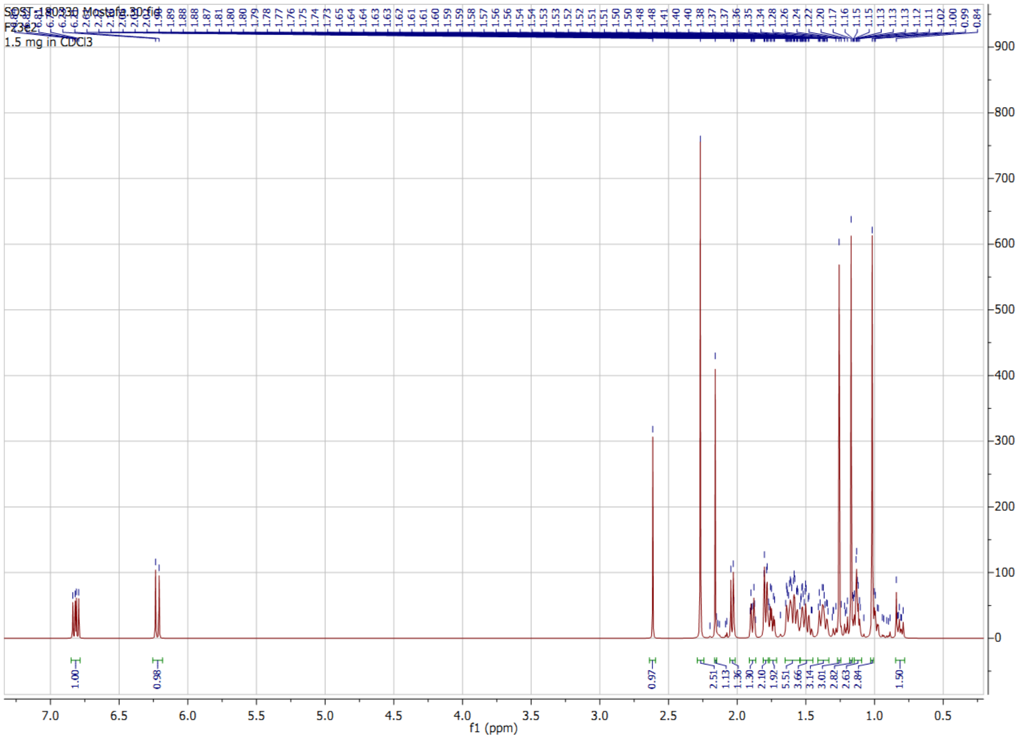

Figure S53. Expand of ^{1}H NMR spectrum of compound **7** (600 MHz, $CDCl_3$)

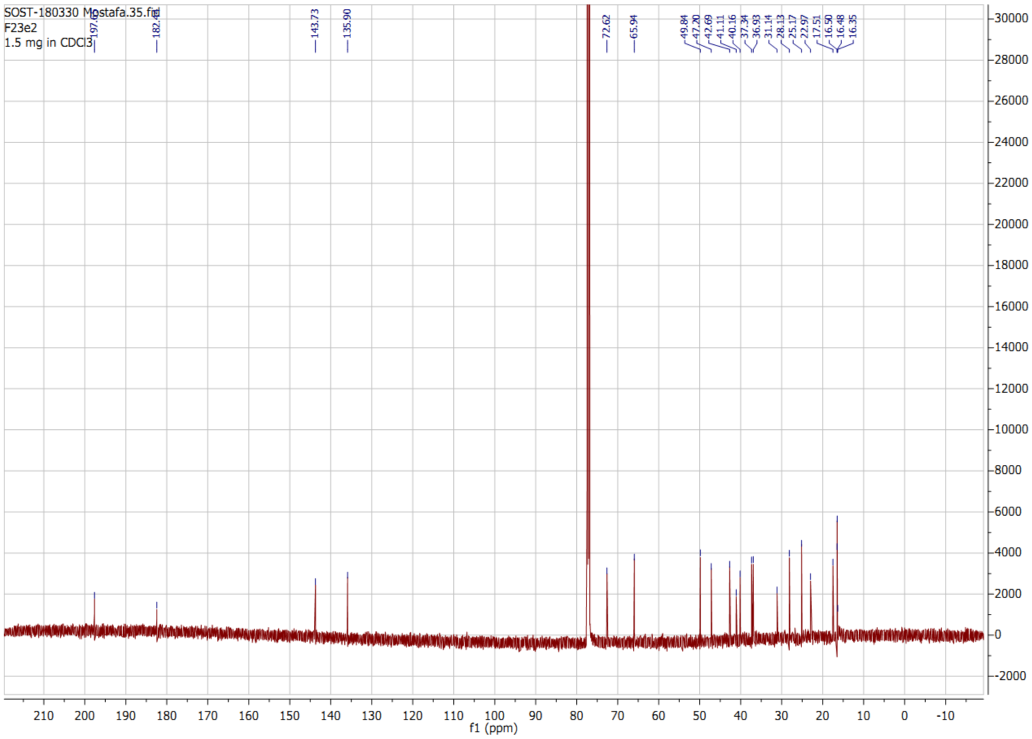

Figure S54. ^{13}C NMR spectrum of compound **7** (600 MHz, $CDCl_3$)

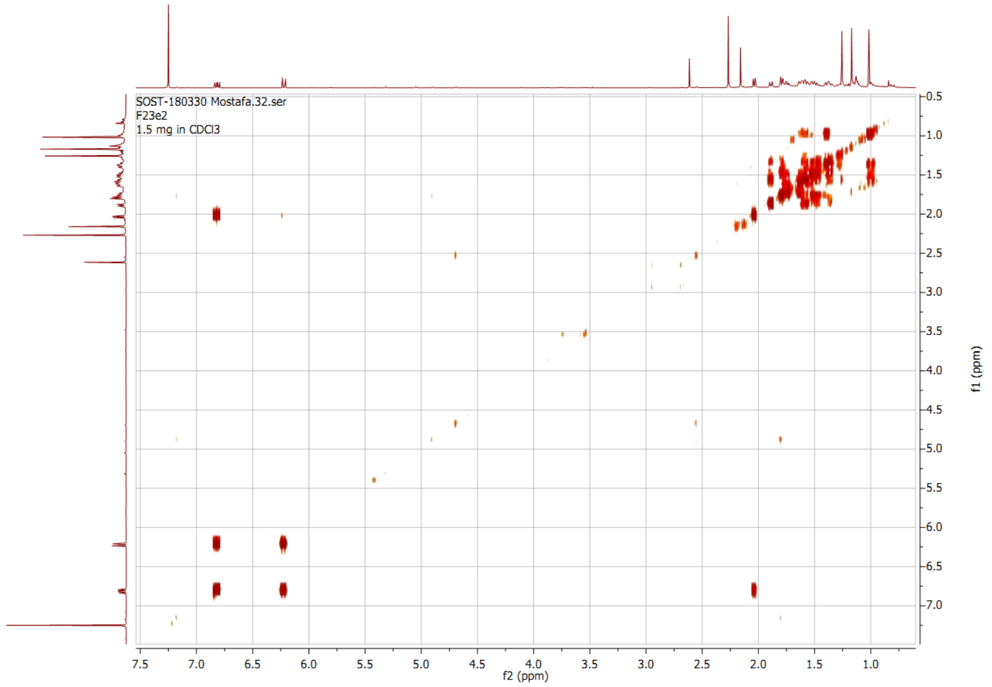

Figure S55. COSY spectrum of compound **7** (600 MHz, $CDCl_3$)

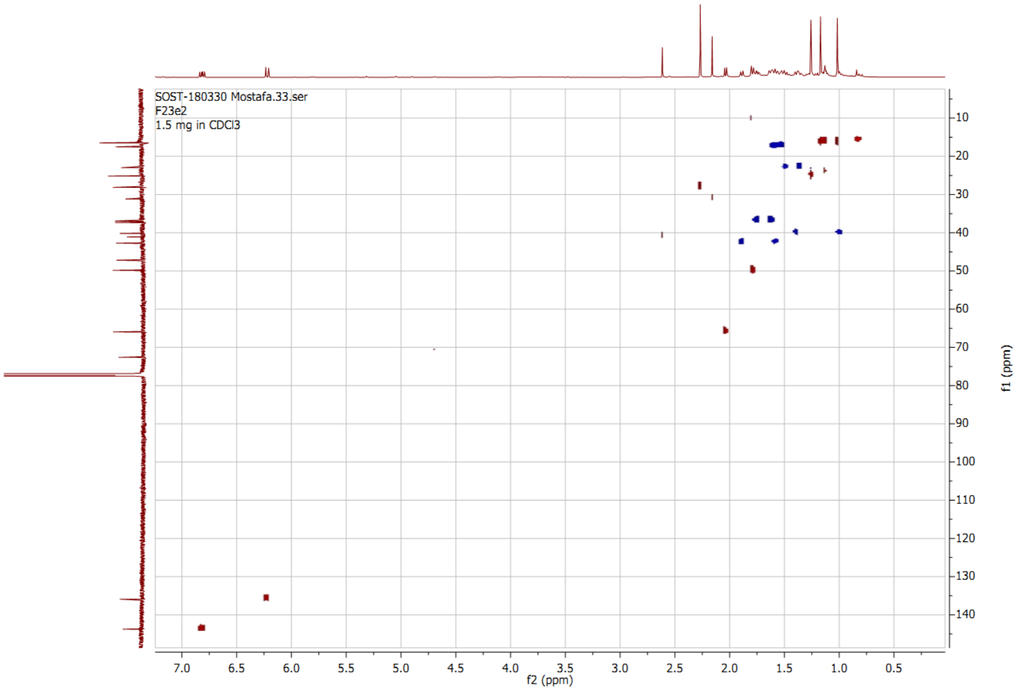

Figure S56. HSQC-DEPT spectrum of compound **7** (600 MHz, $CDCl_3$)

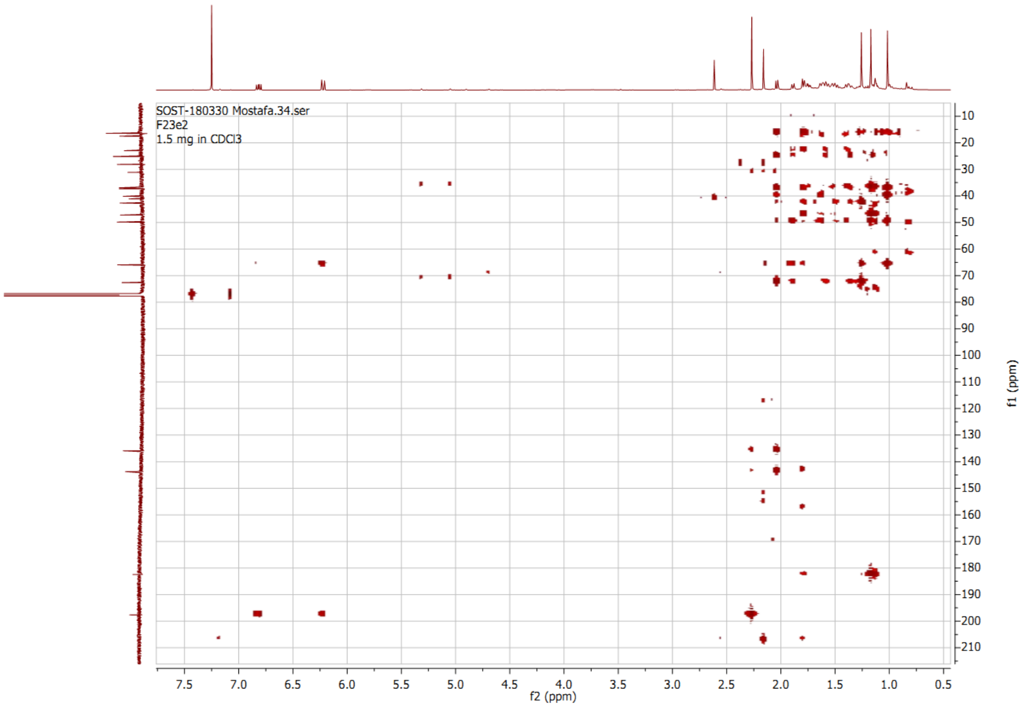

Figure S57. HMBC spectrum of compound **7** (600 MHz, $CDCl_3$)

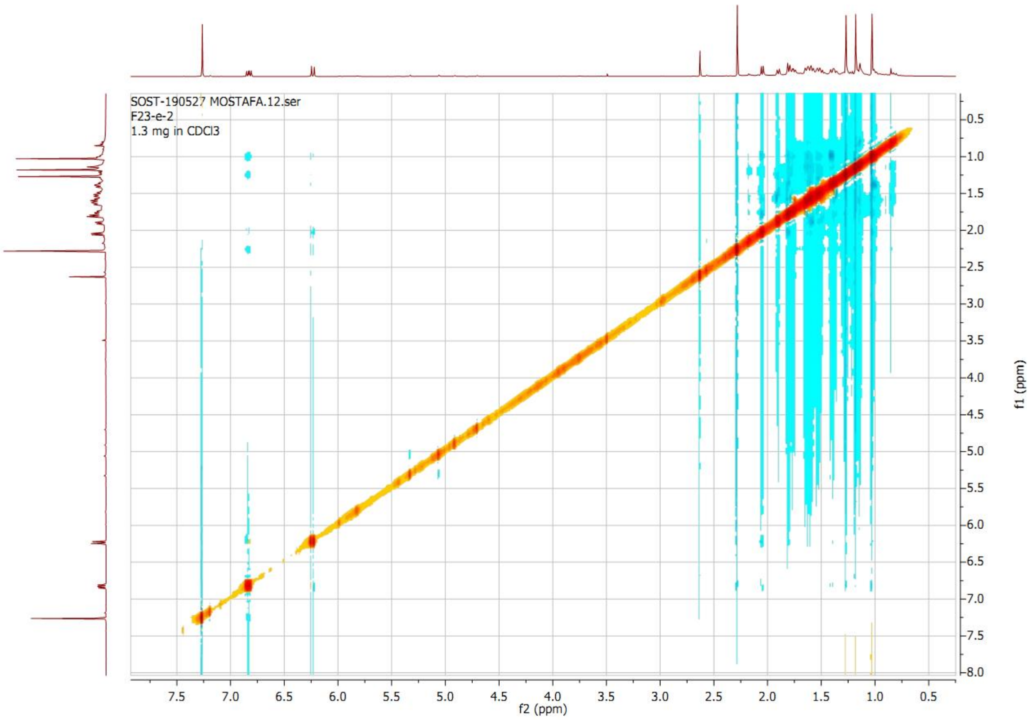

Figure S58. NOESY spectrum of compound **7** (600 MHz, $CDCl_3$)

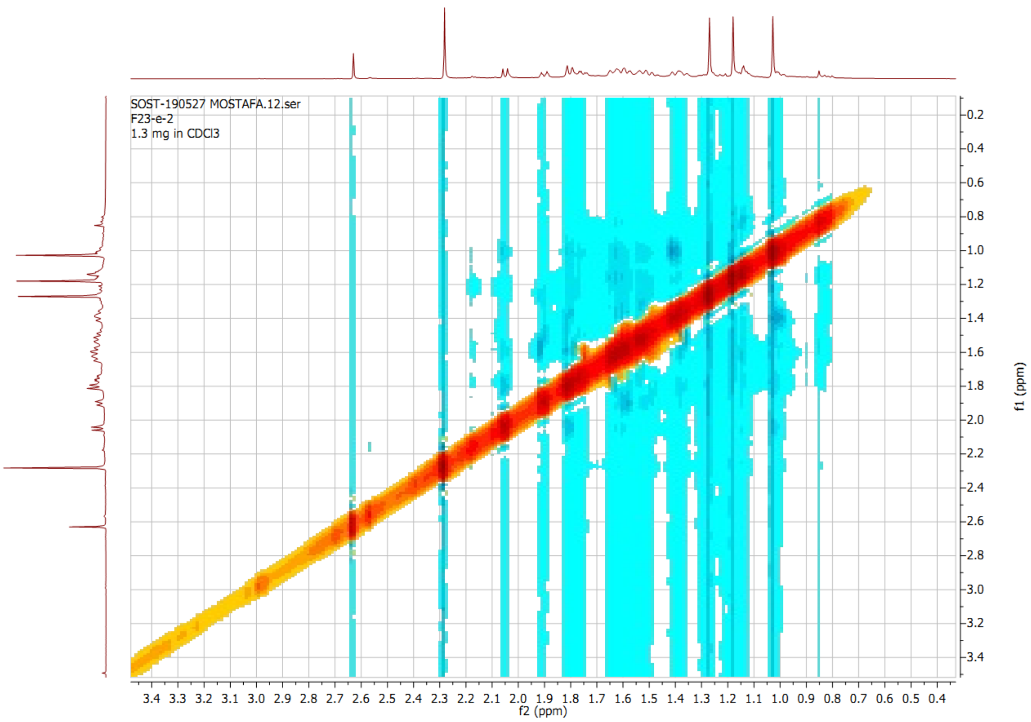

Figure S59. Expand of NOESY spectrum of compound **7** (600 MHz, $CDCl_3$)

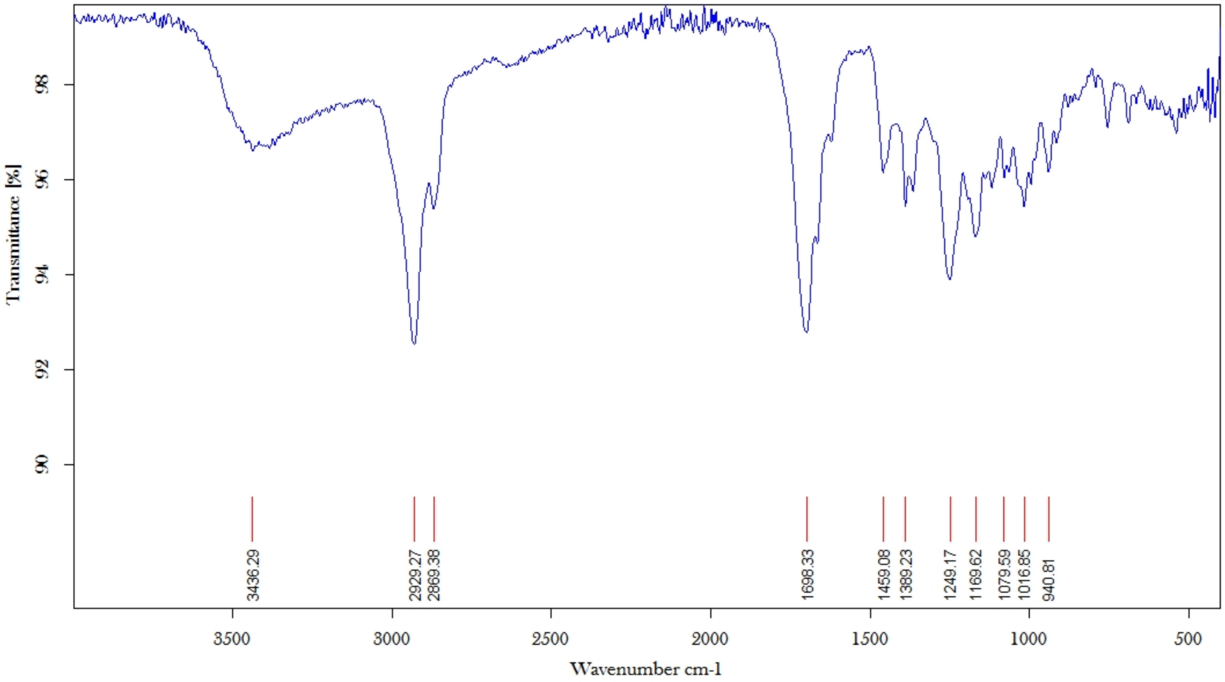

Figure S60. IR spectrum of compound **7**

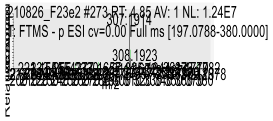

Figure S61. HRESIMS chromatogram of compound **7**

Table 1. Cartesian coordinates for the low-energy optimized conformer of **1** at B3LYP/6-31G (d,p) level of theory in MeOH

Atom type	X	Y	Z
C	2.593	3.857	0.897
C	1.97	2.88	-0.062
C	0.771	3.434	-0.809
C	-0.282	2.45	-1.362
C	-1.089	1.614	-0.326
C	-2.186	2.451	0.43
C	-3.195	3.191	-0.46
O	-1.511	3.442	1.251
C	-2.921	1.566	1.453
C	-3.443	0.251	0.874
C	-2.28	-0.547	0.266
C	-2.625	-2.06	0.015
C	-3.8	-2.307	-0.949
C	-3.016	-2.678	1.367
O	-2.064	-2.525	2.316
O	-4.055	-3.265	1.606
C	-1.342	-2.795	-0.474
C	-0.681	-2.091	-1.661
C	-0.364	-0.621	-1.351
C	-1.597	0.225	-0.917
C	-2.504	0.438	-2.155
C	2.437	1.625	-0.283

C	3.575	1.004	0.343
C	3.938	-0.286	0.138
C	5.044	-1.049	0.689
C	6.064	-0.504	1.636
C	4.954	-2.306	0.18
C	3.793	-2.377	-0.698
O	3.204	-1.113	-0.701
O	3.317	-3.283	-1.353
H	1.892	4.077	1.712
H	2.788	4.812	0.392
H	3.529	3.511	1.338
H	1.165	4.015	-1.659
H	0.29	4.17	-0.162
H	0.219	1.776	-2.061
H	-0.964	3.036	-1.988
H	-0.387	1.338	0.472
H	-3.833	2.512	-1.027
H	-2.702	3.856	-1.176
H	-3.842	3.803	0.176
H	-1.585	4.299	0.812
H	-3.731	2.151	1.902
H	-2.21	1.343	2.259
H	-4.226	0.441	0.131
H	-3.925	-0.322	1.675
H	-1.505	-0.58	1.043
H	-4.695	-1.76	-0.642
H	-4.055	-3.369	-0.96
H	-3.552	-2.009	-1.966
H	-2.4	-2.952	3.123
H	-1.592	-3.831	-0.73
H	-0.625	-2.836	0.353
H	-1.312	-2.174	-2.554
H	0.256	-2.606	-1.903
H	0.11	-0.18	-2.234
H	0.382	-0.597	-0.548
H	-2.514	-0.44	-2.803
H	-3.543	0.652	-1.899
H	-2.139	1.269	-2.765
H	1.909	1.002	-0.994
H	4.196	1.571	1.028
H	6.602	0.339	1.188
H	5.59	-0.135	2.551
H	6.791	-1.271	1.909

H	5.605	-3.148	0.368

Table 2. Cartesian coordinates for the low-energy optimized conformer of **2** at B3LYP/6-31G (d, p) level of theory in MeOH

Atom type	X	Y	Z
C	2.419	3.163	1.452
C	2.25	2.55	0.06
O	1.839	3.598	-0.836
C	3.602	2.084	-0.481
C	4.067	0.766	0.134
C	3.019	-0.329	-0.085
C	3.549	-1.771	0.187
C	4.035	-2.024	1.618
C	4.742	-2.006	-0.738
O	4.454	-1.803	-2.036
O	5.85	-2.35	-0.388
C	2.449	-2.785	-0.213
C	1.105	-2.48	0.442
C	0.637	-1.056	0.14
C	1.65	0.032	0.57
C	1.672	0.112	2.111
C	1.215	1.395	-0.065
C	-0.215	1.846	0.313
C	-1.122	2.021	-0.919
C	-2.51	2.491	-0.564
C	-2.682	3.976	-0.426
C	-3.548	1.661	-0.349
C	-3.502	0.219	-0.444
C	-4.542	-0.595	-0.207
O	-5.784	-0.104	0.161
C	-6.641	-1.173	0.332
C	-5.883	-2.39	0.055
C	-4.622	-2.048	-0.269
C	-3.479	-2.932	-0.631
O	-7.792	-1	0.655
H	1.463	3.447	1.901
H	3.025	4.067	1.355
H	2.923	2.493	2.148
H	1.137	4.102	-0.406
H	4.344	2.873	-0.322
H	3.498	1.958	-1.565

H	4.283	0.897	1.199
H	5.018	0.478	-0.328
H	2.808	-0.325	-1.162
H	3.214	-2.002	2.331
H	4.777	-1.285	1.928
H	4.51	-3.005	1.678
H	5.265	-1.981	-2.539
H	2.778	-3.798	0.043
H	2.325	-2.755	-1.3
H	1.163	-2.645	1.522
H	0.358	-3.19	0.072
H	-0.329	-0.886	0.628
H	0.465	-0.968	-0.94
H	0.913	0.809	2.474
H	1.452	-0.852	2.57
H	2.632	0.441	2.51
H	1.202	1.204	-1.146
H	-0.688	1.131	0.99
H	-0.195	2.788	0.87
H	-0.654	2.749	-1.59
H	-1.157	1.077	-1.47
H	-3.688	4.244	-0.098
H	-1.965	4.391	0.291
H	-2.485	4.474	-1.383
H	-4.509	2.089	-0.077
H	-2.573	-0.267	-0.72
H	-6.314	-3.379	0.115
H	-3.78	-3.979	-0.623
H	-3.1	-2.687	-1.627
H	-2.651	-2.803	0.073

Table 3. Cartesian coordinates for the low-energy optimized conformer of **3** at B3LYP/6-31G (d) level of theory in gas phase

Atom type	X	Y	Z
C	4.541215	-1.04897	-0.13362
C	3.278061	-0.12832	-0.29509
C	2.49012	0.207218	1.02024
C	2.156121	-1.14608	1.711022
C	3.38144	-2.04436	1.93519
C	4.119737	-2.34121	0.625949
C	3.531174	1.126659	-1.14702

C	2.201524	1.758065	-1.57123
C	1.296413	2.119334	-0.38292
C	1.134157	0.881028	0.56414
C	0.140935	1.135496	1.738789
C	-1.2433	0.441264	1.6167
C	-2.25068	1.134472	0.708006
C	-2.80382	2.309084	1.04532
C	-2.63786	0.457263	-0.6024
C	-3.49291	-0.805	-0.39822
O	-1.49724	0.034155	-1.33922
C	-4.85519	-0.53932	0.246494
C	-5.73825	-1.76333	0.373553
C	-6.86816	-1.5374	-0.30857
C	-6.82133	-0.19087	-0.91466
O	-5.61283	0.377814	-0.56247
C	-5.33685	-2.97082	1.157707
O	-7.63241	0.386043	-1.59431
C	3.261174	1.105385	2.021847
C	5.754889	-0.3826	0.540497
C	1.76624	3.429212	0.26971
C	4.993018	-1.47214	-1.54007
O	6.100284	-1.31913	-2.00912
O	4.004316	-2.0752	-2.25502
O	0.006744	2.389219	-0.9989
H	2.577359	-0.74269	-0.87658
H	1.435372	-1.69137	1.084396
H	1.661969	-0.97261	2.674529
H	3.055628	-2.99105	2.384774
H	4.064576	-1.58966	2.663106
H	5.008758	-2.95551	0.819425
H	3.461567	-2.9307	-0.02266
H	4.100223	0.865877	-2.04768
H	4.148812	1.854912	-0.60741
H	1.655169	1.046006	-2.20326
H	2.366701	2.654622	-2.18064
H	0.660334	0.132393	-0.08203
H	-0.02993	2.207416	1.893218
H	0.587145	0.78978	2.675879
H	-1.67491	0.389767	2.62488
H	-1.09134	-0.59125	1.284962
H	-3.52469	2.805241	0.399849
H	-2.57961	2.801921	1.98996
H	-3.23413	1.167979	-1.19181
H	-2.93774	-1.52575	0.215213
H	-3.64028	-1.26346	-1.38268
H	-0.93784	0.830649	-1.46786
H	-4.71306	-0.0636	1.227359

H	-7.71992	-2.19442	-0.43058
H	-5.07875	-2.70188	2.190345
H	-6.14055	-3.71157	1.186347
H	-4.44855	-3.4471	0.723318
H	2.568855	1.666955	2.657505
H	3.891662	0.518416	2.694279
H	3.913363	1.832201	1.532503
H	5.563537	-0.16444	1.591576
H	6.622174	-1.04595	0.481313
H	6.032423	0.549675	0.040854
H	2.808397	3.386288	0.592514
H	1.670869	4.233866	-0.46648
H	1.162449	3.702589	1.141903
H	4.404169	-2.30339	-3.11522
H	-0.60851	2.702296	-0.31107

Table 4. Cartesian coordinates for the low-energy optimized conformer of **4** at B3LYP/6-31G (d) level of theory in gas phase

Atom type	X	Y	Z
C	-4.75357	-0.07258	-0.12048
C	-3.20145	-0.33504	-0.13742
C	-2.29002	0.823631	0.402861
C	-2.68523	2.114719	-0.36948
C	-4.18652	2.431624	-0.31523
C	-5.03461	1.273468	-0.84734
C	-2.79758	-1.70319	0.438347
C	-1.35449	-2.03628	0.042646
C	-0.33184	-0.97219	0.491717
C	-0.80792	0.444007	0.017201
C	0.242913	1.544598	0.337801
C	1.204353	1.830906	-0.84306
C	2.459168	2.576823	-0.43794
C	2.671596	3.866637	-0.71725
C	3.483442	1.75832	0.337084
C	4.293691	0.825826	-0.57672
O	4.451047	2.547067	1.032672
C	4.952477	-0.31577	0.212466
C	5.716151	-1.30191	-0.64243
C	5.083283	-2.48397	-0.58863
C	3.898824	-2.35841	0.278673
O	3.905671	-1.10218	0.817508

C	6.943426	-0.92425	-1.4057
O	3.010577	-3.15211	0.514383
C	-2.41953	1.096228	1.923637
C	-5.40285	-0.09469	1.2762
C	-0.03628	-1.13027	1.995134
C	-5.35672	-1.16627	-1.01797
O	-5.11749	-1.29723	-2.20087
O	-6.19926	-2.0158	-0.37613
O	0.895928	-1.18017	-0.22665
H	-2.96906	-0.38629	-1.21081
H	-2.39151	1.997718	-1.42285
H	-2.12604	2.975497	0.015406
H	-4.3875	3.327348	-0.91691
H	-4.48862	2.688357	0.707983
H	-6.10322	1.514812	-0.76775
H	-4.81987	1.132434	-1.91256
H	-3.46258	-2.48712	0.055251
H	-2.91264	-1.72313	1.529273
H	-1.28897	-2.11143	-1.05018
H	-1.05836	-3.01514	0.443895
H	-0.82688	0.342817	-1.07785
H	0.829393	1.248931	1.211642
H	-0.24056	2.486065	0.617799
H	0.672064	2.402389	-1.61361
H	1.474197	0.867383	-1.287
H	3.584194	4.369592	-0.4107
H	1.942596	4.454942	-1.26889
H	2.940335	1.122899	1.050076
H	5.057838	1.426199	-1.0842
H	3.645459	0.379902	-1.33713
H	3.965108	3.123835	1.643267
H	5.567696	0.097144	1.02091
H	5.333267	-3.40359	-1.10188
H	7.366571	-1.78372	-1.93241
H	6.722646	-0.14298	-2.14413
H	7.710075	-0.51561	-0.73494
H	-2.58549	0.190171	2.510396
H	-1.51126	1.571537	2.308159
H	-3.2456	1.775914	2.148262
H	-5.06778	0.748637	1.881645
H	-6.49196	-0.03008	1.189559
H	-5.17395	-1.01479	1.820394
H	0.456796	-2.09735	2.153707

H	0.642918	-0.35952	2.368842
H	-0.93841	-1.12015	2.610837
H	-6.49382	-2.64992	-1.0578
H	1.356788	-1.95221	0.152367

Table 5. Cartesian coordinates for the low-energy optimized conformer of **5** at B3LYP/6-31G (d) level of theory in gas phase

Atom type	X	Y	Z
C	4.076471	1.068785	-0.16519
C	2.688514	0.434037	0.205335
C	2.430467	-1.0274	-0.30851
C	2.71068	-1.04616	-1.83866
C	4.082838	-0.4734	-2.2233
C	4.275988	0.955508	-1.70555
C	2.312394	0.591719	1.688219
C	0.825418	0.282757	1.903761
C	0.400986	-1.10687	1.388164
C	0.884187	-1.29473	-0.08465
C	0.365769	-2.58485	-0.7603
C	-1.10182	-2.9717	-0.49429
C	-2.16047	-1.86466	-0.63464
C	-1.85152	-0.70209	0.407667
C	-3.05298	-0.03631	1.026551
C	-3.9088	0.757599	0.368219
C	-5.07219	1.503562	0.843541
C	-5.59643	2.148834	-0.22212
C	-4.80455	1.838711	-1.41743
O	-3.77105	0.977872	-0.99743
C	-5.53682	1.513071	2.265124
O	-4.90888	2.17451	-2.56739
C	3.319208	-2.10678	0.362048
C	5.285253	0.489307	0.592844
C	0.830465	-2.20749	2.368694
C	4.015923	2.565274	0.176388
O	4.795443	3.168347	0.882429
O	2.970941	3.204212	-0.41869
O	-1.0576	-1.17054	1.487367
C	-3.55107	-2.48998	-0.44003
O	-2.04238	-1.34062	-1.9591
H	1.965112	1.045056	-0.35149

H	1.932267	-0.46151	-2.35013
H	2.63054	-2.07045	-2.22044
H	4.176826	-0.47333	-3.31667
H	4.88807	-1.12185	-1.85651
H	5.274662	1.326453	-1.97078
H	3.551606	1.61103	-2.2027
H	2.513981	1.616912	2.023553
H	2.931711	-0.05365	2.322521
H	0.230879	1.039115	1.372749
H	0.557965	0.362901	2.964749
H	0.417826	-0.47515	-0.6433
H	0.98271	-3.44866	-0.48177
H	0.48387	-2.46121	-1.84017
H	-1.21013	-3.4017	0.505121
H	-1.36905	-3.75952	-1.20951
H	-1.32406	0.064547	-0.17145
H	-3.20391	-0.19794	2.089276
H	-6.4573	2.802939	-0.25261
H	-5.7855	0.500032	2.603213
H	-4.75262	1.892228	2.931105
H	-6.42271	2.141857	2.384222
H	3.559935	-1.88391	1.403508
H	2.831278	-3.0855	0.342642
H	4.27002	-2.22574	-0.1634
H	6.180242	1.075625	0.36698
H	5.140556	0.533181	1.675901
H	5.476957	-0.54728	0.314376
H	0.220744	-2.11519	3.273174
H	0.669351	-3.20706	1.955224
H	1.879152	-2.12099	2.657348
H	3.046355	4.137679	-0.14526
H	-3.68475	-2.85647	0.584828
H	-4.34742	-1.76932	-0.64893
H	-3.67225	-3.33164	-1.12923
H	-2.68234	-0.61229	-2.04515

Table 6. Cartesian coordinates for the low-energy optimized conformer of **6** at B3LYP/6-31G (d) level of theory in gas phase

Atom type	X	Y	Z
C	4.82885	0.696783	-0.19078
C	3.297797	0.402691	-0.00676

C	2.880282	-1.11183	0.026002
C	3.457904	-1.78262	-1.25174
C	4.965703	-1.55378	-1.43652
C	5.325965	-0.06417	-1.45627
C	2.640009	1.210641	1.127356
C	1.108107	1.15333	1.027995
C	0.57089	-0.28818	1.023619
C	1.310837	-1.10757	-0.07335
C	0.615594	-2.46745	-0.25656
C	-0.83553	-2.23257	-0.70366
C	-1.62105	-1.29132	0.234818
C	-2.17055	-2.06058	1.448016
C	-2.77849	-0.62496	-0.56843
C	-3.73316	0.223236	0.292035
O	-2.23356	0.178319	-1.61102
C	-4.86642	0.821201	-0.54801
C	-5.79495	1.747914	0.205182
C	-7.03118	1.234267	0.158613
C	-7.01364	-0.02799	-0.60895
O	-5.71583	-0.23957	-1.0253
C	-5.32541	3.012597	0.849149
O	-7.91026	-0.79052	-0.87258
C	3.376682	-1.90843	1.258575
C	5.708908	0.375917	1.031437
C	0.618931	-0.84916	2.459069
C	4.990705	2.198725	-0.47116
O	5.702986	2.965546	0.140186
O	4.24213	2.625776	-1.52561
O	-0.82002	-0.14554	0.621809
H	2.8469	0.779899	-0.9348
H	2.932081	-1.38415	-2.13193
H	3.259863	-2.86102	-1.23241
H	5.285318	-2.01063	-2.38167
H	5.531245	-2.07049	-0.65149
H	6.411922	0.06351	-1.55343
H	4.872303	0.395589	-2.34192
H	2.953586	2.260304	1.072196
H	2.974792	0.85445	2.108903
H	0.792883	1.634116	0.092735
H	0.642044	1.716007	1.846539
H	1.098644	-0.54902	-0.99838
H	0.648068	-3.06286	0.664227
H	1.122077	-3.0632	-1.02279

H	-1.37531	-3.18491	-0.77902
H	-0.82631	-1.78785	-1.70444
H	-2.48721	-1.37912	2.241727
H	-3.03557	-2.66246	1.144372
H	-1.42936	-2.74668	1.866215
H	-3.35394	-1.41693	-1.05993
H	-3.15672	1.030739	0.759596
H	-4.18342	-0.37268	1.093168
H	-1.50406	0.659401	-1.17742
H	-4.43225	1.318227	-1.42494
H	-7.9404	1.634721	0.588934
H	-6.15852	3.571173	1.284543
H	-4.59822	2.80684	1.645082
H	-4.81987	3.658995	0.119952
H	3.382719	-1.32395	2.180685
H	2.743309	-2.78502	1.429137
H	4.392216	-2.2843	1.114607
H	5.755076	-0.69597	1.225635
H	6.726832	0.7367	0.860483
H	5.342254	0.873073	1.933887
H	-0.18597	-0.3817	3.034188
H	1.561857	-0.60858	2.95418
H	0.489405	-1.93194	2.511276
H	4.437484	3.577444	-1.61579

Functional	Solvent?		Basis Set		Type of Data	
mPW1PW91	PCM		6-31+G(d,p)		Unscaled Shifts	
	Isomer 1	Isomer 2	Isomer 3	Isomer 4	Isomer 5	Isomer 6
sDP4+ (H data)	0.76%	99.24%	-	-	-	-
sDP4+ (C data)	0.28%	99.72%	-	-	-	-
sDP4+ (all data)	0.00%	100.00%	-	-	-	-
uDP4+ (H data)	0.00%	100.00%	-	-	-	-
uDP4+ (C data)	69.64%	30.36%	-	-	-	-
uDP4+ (all data)	0.00%	100.00%	-	-	-	-
DP4+ (H data)	0.00%	100.00%	-	-	-	-
DP4+ (C data)	0.64%	99.36%	-	-	-	-
DP4+ (all data)	0.00%	100.00%	-	-	-	-

Figure S62. Result of DP4+ NMR chemical shift probability calculation for compound **3**.
Isomer 1 is: 4*R*,5*R*,8*R*,9*R*,10*S*,14*R*,16*R*   Isomer 2 is: 4*R*,5*R*,8*R*,9*R*,10*S*,14*S*,16*R*

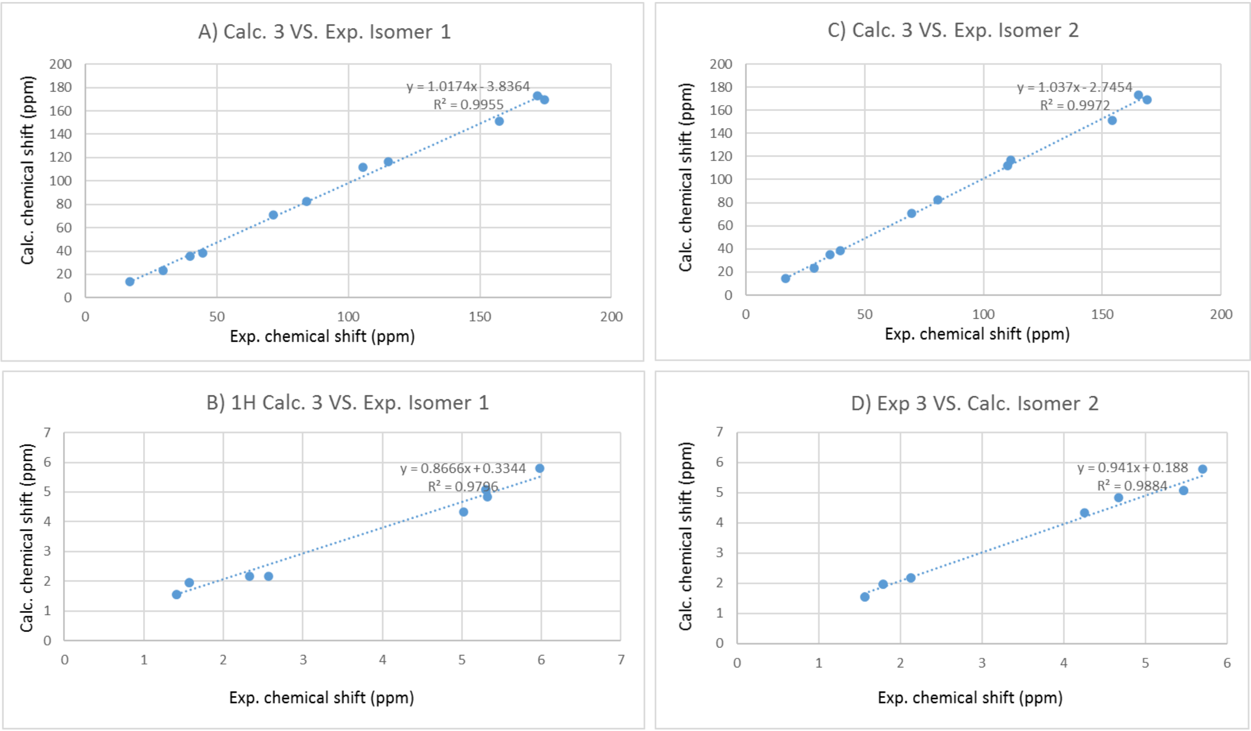

$r_{total} = 1 - \sqrt{(1 - r_C)(1 - r_H)}$

$r_{total\text{-}Isomer\ 1} = 0.990418768$ $\quad r_{total\text{-}Isomer\ 2} = 0.994300877$

Figure S63. Graphs of calculated chemical shifts versus experimental chemicals shifts and ^{1}H, ^{13}C, and total correlation coefficients for two possible isomers of compound **3**.

	A–B	C	D	E	F	G	H
1	Functional	Solvent?		Basis Set		Type of Data	
2	mPW1PW91	PCM		6-31+G(d,p)		Unscaled Shifts	
3							
4		Isomer 1	Isomer 2	Isomer 3	Isomer 4	Isomer 5	Isomer 6
5	sDP4+ (H data)	99.97%	0.03%	-	-	-	-
6	sDP4+ (C data)	0.51%	99.49%	-	-	-	-
7	sDP4+ (all data)	93.65%	6.35%	-	-	-	-
8	uDP4+ (H data)	75.53%	24.47%	-	-	-	-
9	uDP4+ (C data)	86.72%	13.28%	-	-	-	-
10	uDP4+ (all data)	95.27%	4.73%	-	-	-	-
11	DP4+ (H data)	99.99%	0.01%	-	-	-	-
12	DP4+ (C data)	3.24%	96.76%	-	-	-	-
13	DP4+ (all data)	99.66%	0.34%	-	-	-	-

Figure S64. Result of DP4+ NMR chemical shift probability calculation for compound **4**. Isomer 1 is: 4*R*,5*R*,8*R*,9*R*,10*S*,13*R*,14*R*,16*R* Isomer 2 is: 4*R*,5*R*,8*R*,9*R*,10*S*,13*R*,14*S*,16*R*

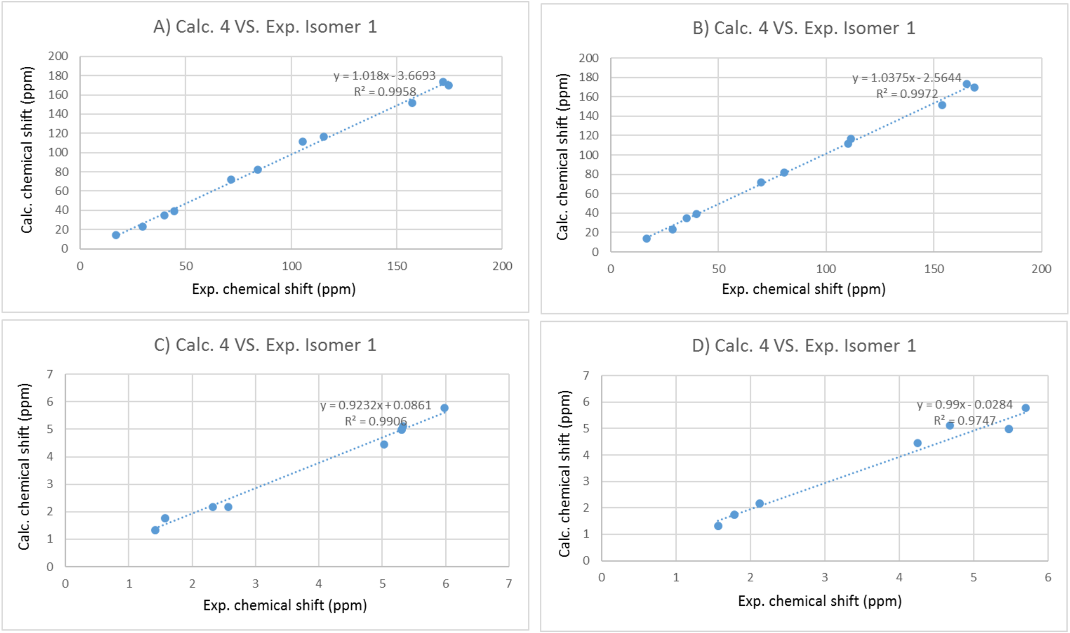

$r_{total} = 1 - \sqrt{(1 - r_C)(1 - r_H)}$

$r_{total\text{-}Isomer\ 1} = 0.993717$ $r_{total\text{-}Isomer\ 2} = 0.991583$

Figure S65. Graphs of calculated chemical shifts versus experimental chemicals shifts and ^{1}H, ^{13}C, and total correlation coefficients for two possible isomers of compound **4**.

1	Functional	Solvent?		Basis Set		Type of Data	
2	mPW1PW91	PCM		6-31+G(d,p)		Unscaled Shifts	
3							
4		Isomer 1	Isomer 2	Isomer 3	Isomer 4	Isomer 5	Isomer 6
5	sDP4+ (H data)	97.22%	2.78%	-	-	-	-
6	sDP4+ (C data)	0.03%	99.97%	-	-	-	-
7	sDP4+ (all data)	1.21%	98.79%	-	-	-	-
8	uDP4+ (H data)	62.53%	37.47%	-	-	-	-
9	uDP4+ (C data)	0.17%	99.83%	-	-	-	-
10	uDP4+ (all data)	0.29%	99.71%	-	-	-	-
11	DP4+ (H data)	98.32%	1.68%	-	-	-	-
12	DP4+ (C data)	0.00%	100.00%	-	-	-	-
13	DP4+ (all data)	0.00%	100.00%	-	-	-	-

Figure S66. Result of DP4+ NMR chemical shift probability calculation for compound **6**. Isomer 1 is: 4*R*,5*R*,8*R*,9*R*,10*S*,13*R*,14*S*,16*R* Isomer 2 is: 4*R*,5*R*,8*R*,9*R*,10*S*,13*R*,14*R*,16*R*
